# Utilization of a Branched Late-Stage Clickable Biotinylated Chassis on the Example of a Pittsburgh B Analogue

**DOI:** 10.1021/acs.orglett.4c02527

**Published:** 2024-07-25

**Authors:** T. Moritz Weber, Pelin Özdüzenciler, Gültekin Tamgüney, Jörg Pietruszka

**Affiliations:** † Mathematisch-Naturwissenschaftliche Fakultät, Institut für Bioorganische Chemie, Heinrich-Heine-Universität Düsseldorf im Forschungszentrum Jülich, 52428 Jülich, Germany; ‡ Institut für Biologische Informationsprozesse 7 (IBI-7: Strukturbiochemie), Forschungszentrum Jülich, 52428 Jülich, Germany; § Mathematisch-Naturwissenschaftliche Fakultät, Institut für Physikalische Biologie, Heinrich-Heine-Universität Düsseldorf, 40225 Düsseldorf, Germany; ∥ Institut für Bio- und Geowissenschaften 1 (IBG-1: Biotechnologie), Forschungszentrum Jülich, 52428 Jülich, Germany

## Abstract

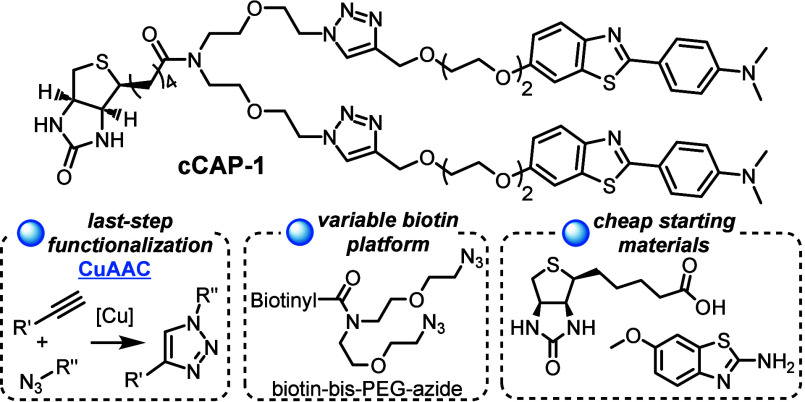

Biotinylation is probably the most frequent and practically useful modification of molecules to facilitate selective and highly affine binding to (strept)avidin for immobilization, enrichment, and purification for further (bio)chemical or (bio)physical investigations. We present a protecting-group-free synthesis of a branched biotin bis-azide that enables dual-payload late-stage functionalization with arbitrary alkynes via click chemistry. Utility of the chassis is briefly showcased on the example of a valuable Pittsburgh B analogue, which binds pathological protein aggregates, commonly found in neurodegenerative diseases.

The highly selective interaction between biotin and the protein (strept)avidin is the most stable noncovalent bond found in nature, characterized by a dissociation constant of 10^–15^ M.^1^ Thus, the interaction is among the most commonly exploited tools in chemistry, biology, and biophysics for surface immobilization, enrichment, and purification.^2−5^ The practical significance of terminally functionalized and activated biotins with flexible polyethylene glycol (PEG) linkers for this purpose is emphasized by the vast number of mostly linear and single-substituted commercial substances. A yet underrepresented group are biotins with bis- or tris-substituted multiarm linkers, displaying multiple units of targetable functional groups. Especially in the field of bioorthogonal click chemistry, symmetrical and unsymmetrical functionalization with non-natural substituents, such as organic azides, terminal alkynes, and cyclooctynes can be used for studies *in vivo* without interfering with the cell’s metabolites.^6,7^ Thus, reliable chemical access to reactive and structurally diverse biotinylated chassis is quite important to study the architecture,^8^ cellular localization,^2^ or interaction partners of molecules in an isolated or cellular context.^9^

A recent innovation in bis-functionalized biotinylated molecular probes was the synthesis of the capture molecule for amyloid precipitation (CAP-1, cf. Figure 1) that utilizes the interaction of biotin/streptavidin to selectively precipitate amyloid fibrils from complex biofluids by streptavidin-coated magnetic beads and employs a rarely met bifurcated amide linker to display two probes per biotin unit and increase the strength of interaction.^1,10^ The *N*-methylated probe is akin to Pittsburgh compound B (PiB, **1**) and is derived from the benzothiazole family. Benzothiazole-containing probes emerged in the late 1990s as potent molecules to selectively bind and detect biomarkers of progressive neurodegenerative diseases with no cure,^11^ such as Alzheimer’s (AD) or Parkinson’s disease (PD).^12−14^ In 2004, Pittsburgh compound B was introduced as a novel amyloid-imaging positron emission tomography (PET) tracer to visualize amyloid beta (Aβ) plaques in AD brain tissue.^15^ Furthermore, PiB was shown to bind Lewy body derived α-synuclein (α-syn) fibrils,^16^ which is a pathogenic hallmark of synucleinopathies (e.g., PD and multiple system atrophy), too.^17^ However, brain imaging as a diagnostic tool is expensive and is not ubiquitously available. Because amyloid fibrils are also present in cerebrospinal fluid (CSF), blood, stool, and other biofluids, methods for selectively enriching amyloid fibrils from these more readily accessible sources are being developed as a cheaper and more universal way to diagnose amyloid diseases and monitor their progression and the effectiveness of therapeutic interventions.^18−21^

**Figure 1:**
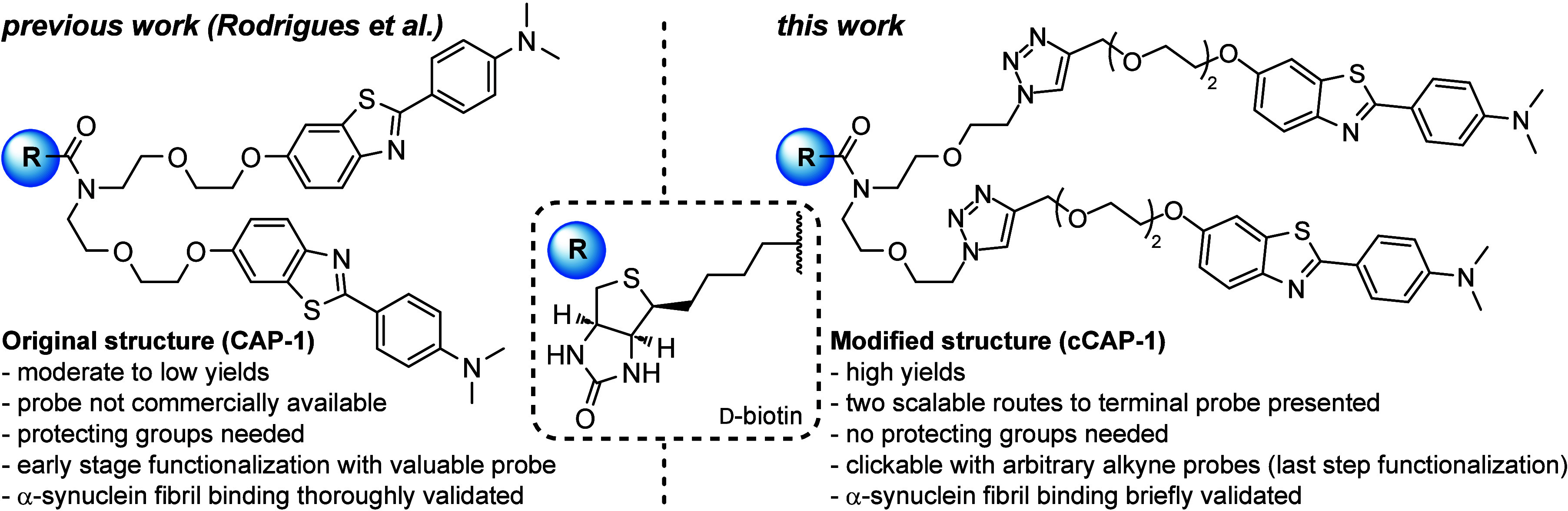
Same but different. A Y-shaped biotin anchor for benzothiazole-based structure specific precipitation of amyloid fibrils.

Inspired by the design of CAP-1, we intend to overcome two important limitations and cost drivers of the presented route by Rodrigues et al.:^10^ As the dual probe is attached to the Boc-protected linker in an early stage of the synthesis, followed by deprotection and late-stage amide coupling with plain d-biotin, the yield over three steps including the valuable benzothiazole probe is 10%, only. However, the *N*-methylated probe is not commercially available, and the Pittsburgh compound B is very pricey (CAS 566169-93-5). Adhering to this, the late-stage attachment of biotin to the probe-decorated linker does not allow simple exchange of the probe and requires a three-step procedure for every additional target molecule, which would prohibit a streamlined preparation of large biotinylated compound libraries for screenings and precludes bioorthogonal application. These downsides prompted us to redesign the CAP-1 molecule, retaining the same benzothiazole probe and the multiarm Y-shaped biotin for utilization of the highly selective biotin–streptavidin interaction for immobilization on streptavidin-functionalized surfaces. In contrast to the synthesis of CAP-1, we chose an approach that provides a symmetrical and ubiquitously usable bifurcated biotin-bis-PEG-azide, derived from a protecting-group-free synthesis. By independent transformations on the biotin and the benzothiazole moiety, both functional units are mergeable in a reliable and commutable last-step copper-catalyzed click reaction, and give rise to the biotin bis-azide chassis, suitable for bioorthogonal applications.

Starting from cheap d-biotin (**2**), the terminal acid was transformed into the corresponding *N*-hydroxy succinimide (NHS) ester for carbonyl activation. The reaction with disuccinimidyl carbonate (DSC) provided the NHS-activated biotin **3** in 95% yield without the need of chromatographic purification (Scheme 1).^22^ The symmetric amino alcohol linker **4** was synthesized in a one-step procedure from 2-(2-chloroethoxy)ethanol (**5**) and an excess of 2-(2-aminoethoxy)ethanol (**6**) in the presence of anhydrous sodium carbonate in refluxing toluene.^23^ Distillation of the residue gave amino alcohol **4** in 66% yield, ready for coupling with activated biotin. Due to the early stage amide coupling, the formation of the tertiary amine reduces the inherent nucleophilicity of the linker nitrogen and redundantizes an additional protecting group strategy, as employed by Rodrigues et al. or in the patented synthesis of biotin-bis-PEG_3_-azide by Esko et al.^10,24,25^ The reaction between the unprotected amino alcohol **4** and NHS-biotin **3** was carried out using triethylamine (TEA) as base, yielding 77% of the bis-hydroxy-functionalized biotin **7** as sticky oil, which was only soluble in DMSO, MeOH, or DMF, limiting the solvent options for further transformations. A direct approach from d-biotin to bis-hydroxybiotin **7** with *in situ* activation of biotin using *N*,*N*′-carbonylide imidazole (CDI) led to product formation in 34% yield. As the terminal hydroxyl groups cannot be transformed directly into the corresponding azide, activation via intermediate alkyl halide or (pseudo)halide was necessary. Typical transformations to the alkyl bromide (Appel reaction) with CBr_4_ and PPh_3_ or alkyl mesylate with mesyl chloride failed or produced several side products. While searching for alternative halogenating reagents, we stumbled over the neglected methyltriphen-oxyphosphonium iodide (MTPPI), which can be prepared in gram scale by the reaction of triphenyl phosphite and methyl iodide under solvent-free conditions.^26^ Conversion of bis-hydroxy-biotin **7** with MTPPI provided the desired bis-iodide-biotin **8** in a good yield of 71% as a colorless oil. Lastly, halide substitution with sodium azide gave access to the clickable Y-shaped bis-azide-biotin **9** (biotin-bis-PEG-azide) as a colorless glass in 87% yield. The apparent advantage of our presented protecting-group-free synthesis is easy access to all three biotin derivatives with branched linker architecture (biotin-bis-PEG_2_-alcohol **7**, -PEG-iodide **8**, and -PEG-azide **9**) with synthetically interesting functional groups, as they can be used for further functional group interconversions. This advantage is not given in the published synthesis of biotin-bis-PEG_3_-azide.^24,25^ As the azide moiety is attached to the biotin unit, click chemistry with alkyne-functionalized probes is not limited to copper-catalyzed CuAAC, but it can also be extended to the strain-promoted SPAAC reactions.

**Scheme 1:**
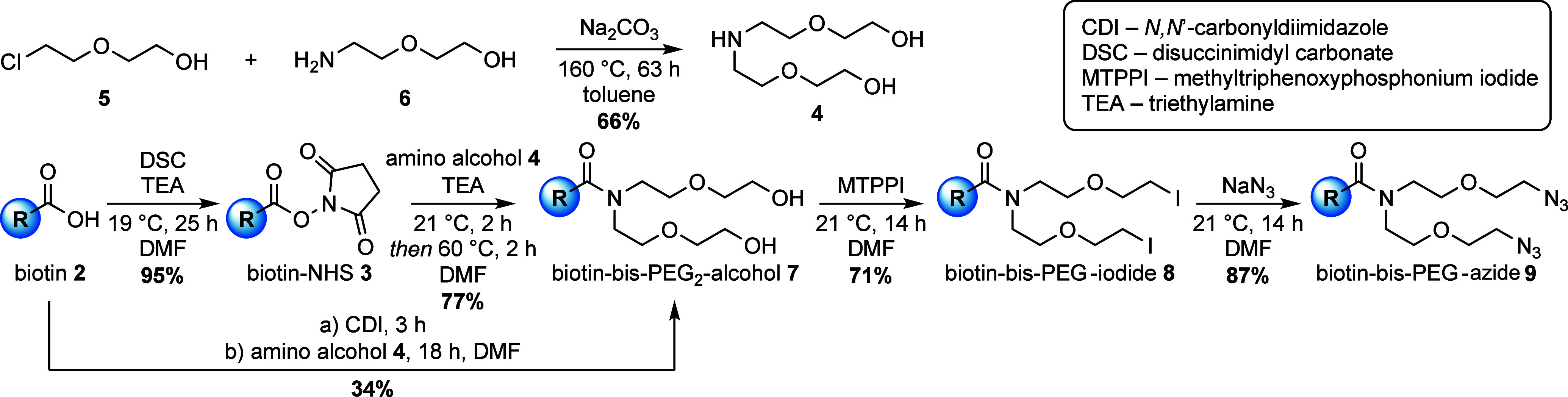
Synthesis of the Y-Shaped Bis-azide-Functionalized Biotin Linker **9** from d-Biotin (**2**)

With the terminal probe of CAP-1 not being commercially available, we sought two individual and reliable approaches, giving rise to *N*-methyl PiB **10** in good yields and with low purification effort. First, commercially available PiB **1** was *N*-methylated in an Eschweiler–Clarke reaction using a modified procedure of Kaur et al. for differently substituted benzothiazoles and of Reed et al. for anilines (Scheme 2).^27,28^ Therefore, PiB **1** was reacted with sodium cyanoborohydride and paraformaldehyde under acidic conditions to give *N*-methyl PiB **10** in 97% yield, with a simple extraction and washing of the solid product. Although this procedure is simple to perform and the product is contrivable in excellent yields, the high cost of PiB **1** is a significant disadvantage of this route.

**Scheme 2:**
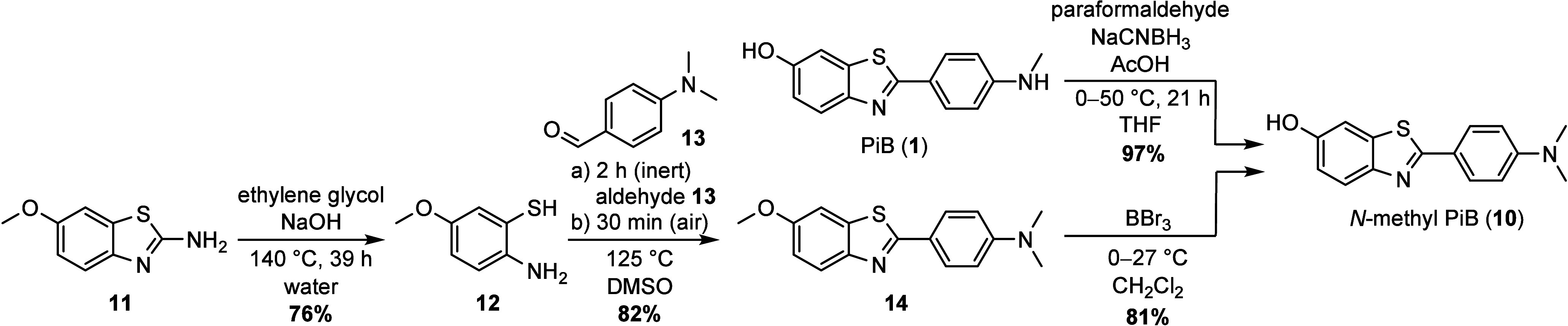
Synthesis of *N*-Methyl PiB **10** from Pittsburgh B (**1**) and 2-Amino-6-methyoxybenzothiazol (**11**)

Second, we aimed to synthesize *N*-methyl PiB **10** from cheap and available sources to offer alternative routes to the valuable probe. As a consequence, 6-methoxybenzothiazol-2-ylamine (**11**, CAS 1747-60-0) was chosen as the starting material and subjected to basic ring opening with NaOH in aqueous solution (Scheme 2). Although the obtained yields varied between 60 and 90%, careful handling under inert atmosphere including utilization of degassed solvent was a prerequisite for a reliable outcome of the reaction, as otherwise the oxygen-induced competing disulfide formation took place (black product instead of bright-yellow). Based on aminothiol **12**, the benzothiazole scaffold was built in an optimized two-step procedure, involving a condensation of 2-amino-5-methoxybenzenethiol (**12**) and 4-(dimethylamino)benzaldehyde (**13**), followed by an intramolecular cyclization and oxidation reaction. The best results in terms of yield and impurities were obtained when aminothiol **12** and aldehyde **13** were forced to form the dihydrobenzo[*d*]thiazole structure under inert atmosphere at 125 °C first, before brief oxidation under air furnished the oxidized benzothiazole **14** in 82% yield, while direct exposure to air led to more undesired side products. In general, we found that the more reactive aldehyde **13** is the superior coupling partner for aminothiol **12**, as the yield drops to 35% when the aldehyde is replaced by the corresponding acid as reported by Huang et al.^29^ Final demethylation of the methoxy group with boron tribromide yielded 81% of *N*-methyl PiB **10** as an amorphous solid in gram quantities without the need of chromatographic purification in any of the presented steps in Scheme 2.

Initially, we aimed to use bis-iodide-biotin **8** for the etherification reaction with PiB **1** or Me-PiB **10** to reproduce the original structure of CAP-1 or an engineered derivative thereof. However, both attempts were not productive and thus dismissed. Instead, click chemistry for last-step functionalization of bis-azide-biotin **9** was selected. The installation of a suitable alkyne tail for the conjunction of the biotin and Me-PiB moieties via click chemistry was based on a diethylene glycol acetylene linker. The precursor **15** was first brominated in quantitative yield in an Appel reaction by substitution of the terminal hydroxy group (Scheme 3). In the following step, the *N*-methylated PiB **10** was *O*-functionalized with the described brominated alkyne linker **16**, similar to the PiB-functionalization of Diner et al.^30^ Etherification of the dummy substrate PiB **1** with alkyne bromide **16** turned out to be challenging because many solvent/base combinations either led to no conversion, low conversion, or undesired side products. The best solution was a heterogenic reaction system of KOH in dry glyme at 80 °C, which was the only combination suitable to render near-full conversion and to present alkyne Me-PiB **17** in a yield of 52%. By utilizing the recently published Cu(I)/Cu(II) ligand TDETA [tris((dimethylethyl)triazolyl)amine; for structure see Supporting Information, Figure S1],^31^ the modified clicked capture molecule cCAP-1 **18** was synthesized in 88% yield from bis-azide-functionalized biotin **9** and the alkyne Me-PiB **17** in a copper-catalyzed azide–alkyne cycloaddition (CuAAC) under reducing conditions.

**Scheme 3:**
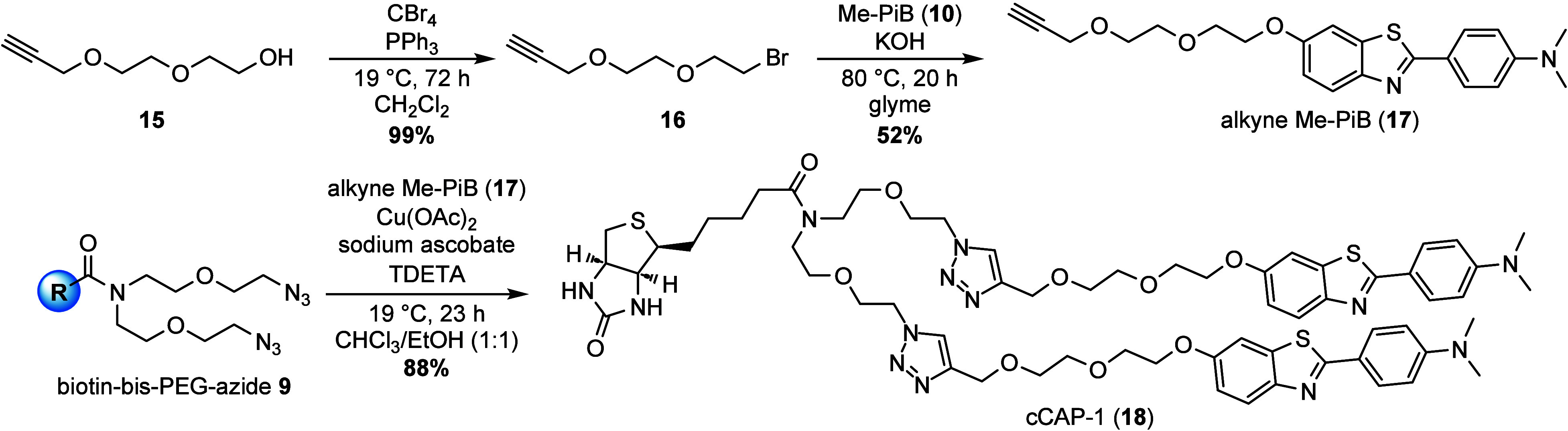
Synthesis of the Clicked Amyloid Capture Molecule cCAP-1 (**18**)

With the biotinylated dual-payload probe cCAP-1 **18** being available for further testing, we first characterized the intrinsic fluorescence in the presence or absence of α-syn fibrils, as fluorescence enhancement upon fibril binding is a common feature of benzothiazoles (e.g., thioflavin T and CAP-1) and can be utilized for imaging purposes of fibrillar protein aggregates or their quantification.^10,11,15,30,32,33^ Upon binding of the *in vitro* generated α-syn fibrils, a 2.8-fold increase of absorption and a 2.9-fold fluorescence enhancement were observed for cCAP-1 **18**, accompanied by a Stokes shift of 52 nm (Figure S2A, Supporting Information). Furthermore, we could show a linear increase of fluorescence of cCAP-1 **18** in a range from 0 to 200 nM in phophate-buffered saline (Figure S2B, Supporting Information), proving comparable fluorescence and solubility properties to the original CAP-1 structure.^10^ A highly specific and affine interplay between streptavidin and biotin or functional biotin moieties is a key prerequisite in several pull-down strategies aiming for purification or quantification of target molecules.^1,10,34−36^ We highlighted the interaction of streptavidin-coated polystyrene plates with preincubated cCAP-1 **18** and purified α-syn fibrils to selectively accumulate α-syn fibrils in comparison to a cCAP-1-free control *in vitro* (Figure S2C, Supporting Information).

In conclusion, we generated a reliable and high yielding synthesis toward branched biotinylated-bis-PEG-azide chassis **9** suitable for late-stage functionalization and rapid library preparation with arbitrary alkyne probes via (bioorthogonal) click chemistry (CuAAC and SPACC). On the example of and inspired by the previously reported compound CAP-1, we showcased the potential of the novel chassis by providing the clicked capture molecule for amyloid precipitation cCAP-1 **18** with dual probe loading as a synthetically more tractable and economic alternative for the detection of protein fibrils associated with neurodegenerative diseases, exemplified on α-synuclein. Furthermore, we were able to present two independent routes leading to the noncommercially available Me-PiB **10** probe, either starting from commercially available but yet expensive PiB **1**, or from cheap and available 6-methoxybenzothiazol-2-ylamine (**11**). Consequently, biochemical in-depth characterization of cCAP-1 will be needed to fully comprehend amyloid- and streptavidin-binding of biotinylated cCAP-1 in combination with complex biofluids and in comparison to well-characterized CAP-1.

## Data Availability

The data underlying this study are available in the published article and its Supporting Information.

## Supporting Information Available

Complete experimental procedures and compound characterization (PDF)
