# Supplementary material for: Utilization of a Branched Late-Stage Clickable Biotinylated Chassis on the Example of a Pittsburgh B Analogue
Source: Org Lett. 2024 Jul 25;26(31):6771–5. doi: 10.1021/acs.orglett.4c02527 (PMC11320650; doi:10.1021/acs.orglett.4c02527)
Supplement: Supplementary file 1 — ol4c02527_si_001.pdf [file ol4c02527_si_001.pdf]

Supporting Information

Utilization of a Branched Late-Stage Clickable Biotinylated Chassis on the Example of a Pittsburgh B Analog

T. Moritz Weber,[1] Pelin Özdüzenciler,[2,3] Gültekin Tamgüney,[2,3] Jörg Pietruszka[1,4]*

[1] Heinrich-Heine-Universität Düsseldorf im Forschungszentrum Jülich, Mathematisch-Naturwissenschaftliche Fakultät, Institut für Bioorganische Chemie, 52428 Jülich, Germany.

[2] Institut für Biologische Informationsprozesse 7 (IBI-7: Strukturbiochemie), Forschungszentrum Jülich, 52428 Jülich, Germany.

[3] Heinrich-Heine-Universität Düsseldorf, Mathematisch-Naturwissenschaftliche Fakultät, Institut für Physikalische Biologie, 40225 Düsseldorf, Germany.

[4] Institut für Bio- und Geowissenschaften 1 (IBG-1: Biotechnologie), Forschungszentrum Jülich, 52428 Jülich, Germany.

*E-mail: j.pietruszka@fz-juelich.de

Table of Contents

1. General information and instrumentation S2

2. Validation of α-synuclein binding of cCAP-1 ..... S3

3. Synthetic procedures S4

   2,2'-((Azanediylbis(ethane-2,1-diyl))bis(oxy))bis(ethan-1-ol) (**4**) S4

   2,5-Dioxopyrrolidin-1-yl 5-((3a*S*,4*S*,6a*R*)-2-oxohexahydro-1*H*-thieno[3,4-*d*]imidazol-4-yl)pentanoate (**3**) S5

   N,*N*-Bis(2-(2-hydroxyethoxy)ethyl)-5-((3a*S*,4*S*,6a*R*)-2-oxohexahydro-1*H*-thieno[3,4-*d*]imidazol-4-yl)pentanamide (**7**) S6

   N,*N*-Bis(2-(2-iodoethoxy)ethyl)-5-((3a*S*,4*S*,6a*R*)-2-oxohexahydro-1*H*-thieno[3,4-*d*]imidazol-4-yl) pentanamide (**8**) S7

   N,*N*-Bis(2-(2-azidoethoxy)ethyl)-5-((3a*S*,4*S*,6a*R*)-2-oxohexahydro-1*H*-thieno[3,4-*d*]imidazol-4-yl)pentanamide (**9**) S8

   2-Amino-5-methoxybenzenethiol (**12**) S9

   4-(6-Methoxybenzo[*d*]thiazol-2-yl)-*N*,*N*-dimethylaniline (**14**) S10

   2-(4-(Dimethylamino)phenyl)benzo[*d*]thiazol-6-ol (**10**) S11

   2-(4-(Dimethylamino)phenyl)benzo[*d*]thiazol-6-ol (**10**) S12

   3-(2-(2-Bromoethoxy)ethoxy)prop-1-yne (**16**) S13

   N,*N*-Dimethyl-4-(6-(2-(2-(prop-2-yn-1-yloxy)ethoxy)ethoxy)benzo[*d*]thiazol-2-yl)aniline (**17**) S14

   N,*N*-Bis(2-(2-(4-((2-(2-((2-(4-(dimethylamino)phenyl)benzo[*d*]thiazol-6-yl)oxy)ethoxy)ethoxy)methyl)-1*H*-1,2,3-triazol-1-yl)ethoxy)ethyl)-5-((3a*S*,4*S*,6a*R*)-2-oxohexahydro-1*H*-thieno[3,4-*d*]imidazol-4-yl)pentanamide (**18**, cCAP-1) S15

4. Biochemical procedures S16

5. NMR Spectra S17

6. References S56

1. General information and instrumentation

All commercial chemicals were purchased from Sigma Aldrich (Merck), Tokyo Chemical Industry (TCI), and ThermoScientific in purities >97%. 2-(4-Methylamino)phenyl)benzo[*d*]thiazol-6-ol (Pittsburgh Compound B, PiB, **1**) was acquired from BLDpharm in a purity of 95%. Purification of technical grade organic solvents from VWR or Carl Roth was achieved by distillation. Anhydrous toluene and THF were obtained from the MBraun MB-SPS 800 solvent purification system. According to the procedure of Burfield and Smithers, extra dry DMF was prepared by two-times sequential drying over activated 3 Å molecular sieve for ≥72 h.[1] ^{1}H, ^{13}C, DEPT135, ^{1}H-^{1}H-COSY, ^{1}H-^{13}C-HSQC, ^{1}H-^{13}C-HMBC, ^{1}H-^{1}H-ROESY, and ^{1}H-^{1}H-TOCSY NMR data were collected on a Bruker Avance/DRX 600 spectrometer. For this purpose, $CDCl_3$, DMSO-d_6, or MeOD were used as solvents and their respective chemical shifts ($CDCl_3$: ^{1}H – 7.26 ppm, ^{13}C – 77.16 ppm; DMSO-d_6: ^{1}H – 2.50 ppm, ^{13}C – 39.52 ppm, MeOD: ^{1}H – 3.31 ppm, ^{13}C – 49.00 ppm) used for referencing. NMR assignments are based on 1D and 2D correlation NMR spectra and the atom numbers in NMR assignments within the experimental section refer to the corresponding atom numbering of the structures within the NMR spectra (chapter 5. NMR spectra). Structural assignments were made with additional information from gCOSY, gHSQC, and gHMBC experiments. High-resolution mass spectrometry (HRMS) data were recorded on a Bruker maXis instrument (at CeMSA in Düsseldorf) with a standard collision energy of 8 eV. For melting point determination, the Büchi melting point apparatus B-540 was employed with a temperature increment of 2 °C/min. Thin layer chromatography (TLC) was performed using coated Macherey-Nagel POLYGRAM SIL G/UV_{254} or POLYGRAM ALOX N/UV_{254} plates (40 x 80 x 0.2 mm) and for standard preparative flash chromatography Macherey-Nagel silica gel 60 (40–63 µm, 230–400 mesh) or ACROS Organics neutral aluminium oxide (Brockmann I, 40–300 µm) was used. Visualization of different compounds on TLC plates was achieved using various staining methods, including excitation under UV light, $KMnO_4$ stain (7.5 g/L $KMnO_4$, 50 g/L K_2CO_3, 625 mg/L NaOH in water), or CAM stain (25 g/L phosphomolybdic acid, 10 g/L $Ce(SO_4)_2$ x 4 H_2O, 60 mL/L conc. H_2SO_4, fill up with water). The copper ligand TDETA (Figure S1) was synthesized as published earlier.[2]

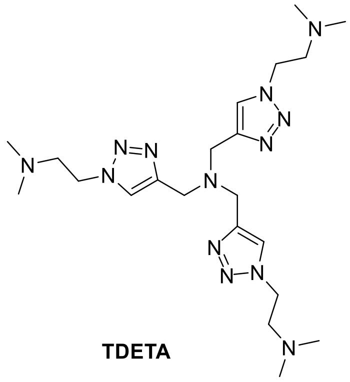

TDETA

Figure S1. Structure of the Cu(I)/Cu(II) ligand TDETA used for the CuAAC between biotin-bis-PEG-azide **9** and alkyne Me-PiB **17** (cf. Scheme 3 in the main article).[2]

2. Validation of α-synuclein binding of cCAP-1

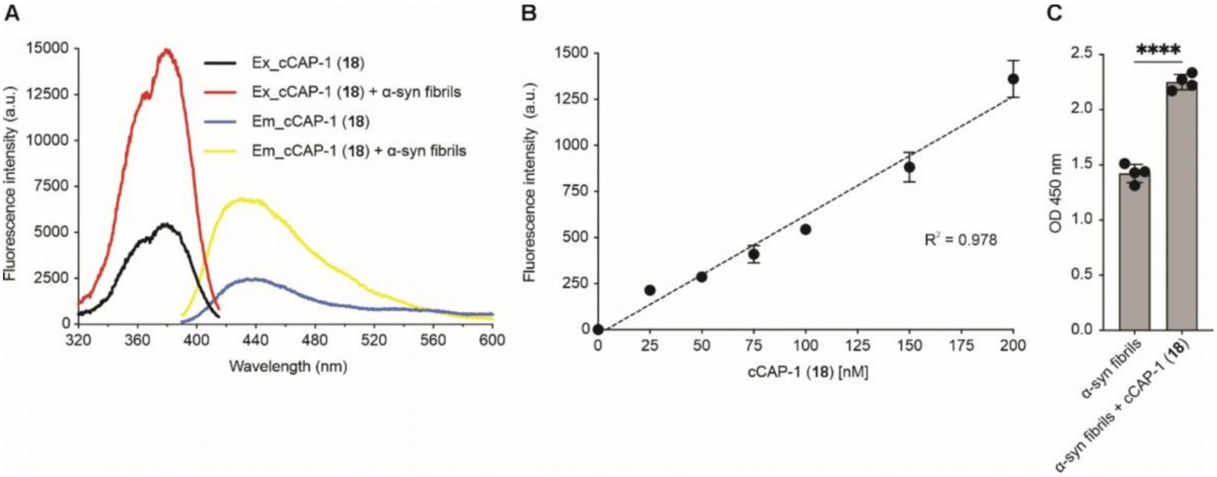

Figure S2. Spectral validation of cCAP-1 (**18**) and its streptavidin-binding for purification of α-syn fibrils. **A**. cCAP-1 binds to α-syn fibrils and allows their detection in aqueous solution via fluorescence measurements. Fluorescence excitation and emission spectra for 20 µM cCAP-1 were measured in the absence and presence of 10 µM (monomer equivalent) α-syn fibrils and demonstrate that cCAP-1 binds to α-syn fibrils and enables their detection in aqueous solution by showing increased absorption (2.8-fold) and enhanced fluorescence emission (2.9-fold) after excitation at 365 nm. Excitation maximum and emission maximum of cCAP-1 at 380 nm and 433.5 nm were slightly shifted in the presence of α-syn fibrils to 379 nm and 431 nm, respectively, giving a Stokes shift of 52 nm. **B**. cCAP-1 shows good solubility and binding to α-syn fibrils in aqueous solution. Fluorescence measurements with increasing concentrations (0–200 nM) of cCAP-1 in the presence of 10 µM (monomer equivalent) α-syn fibrils show a linear relationship for the binding of cCAP-1 to α-syn fibrils and good solubility in aqueous solution. The experiment was performed in triplicates. **C**. cCAP-1 allows improved purification of α-syn fibrils from aqueous solution. Indirect ELISA measurements showed that preincubation of 0.34 µM (monomer equivalent) α-syn fibrils with 10 µM cCAP-1 resulted in a 58% increase in binding of α-syn fibrils to streptavidin-coated 96-well polystyrene plates. The experiment was performed in quadruplicates. Error bars indicate standard deviation. ****P < 0.0001.

3. Synthetic procedures

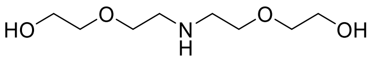

2,2'-((Azanediylbis(ethane-2,1-diyl))bis(oxy))bis(ethan-1-ol)[3] (4, CAS 54384-47-3)

In a 1 L three-necked flask under N_2 atmosphere, a solution of 2-(2-chloroethoxy)ethanol (**5**) (25.2 g, 202 mmol, 1.00 equiv.) in 50 mL of dry toluene (0.25 mL/mmol chloride) was added dropwise to a refluxing mixture of 2-(2-aminoethoxy)ethanol (**6**) (53.1 g, 505 mmol, 2.50 equiv.) and sodium carbonate (23.6 g, 222 mmol, 1.10 equiv.) in 500 mL of dry toluene (2.50 mL/mmol chloride). The mixture was stirred under reflux at 160 °C for 63 h using a heating mantle. A glass column filled with 50 mL activated 4 Å molecular sieve and a condenser were placed on top of the reaction flask. The molecular sieve was washed with toluene and the wash fraction added to the reaction vessel. The solid is removed by filtration and the filtrate dried with 60 g $MgSO_4$ to clear the solution. The solution is filtered again and the solvent removed under reduced pressure. The solids from first and second filtration (NaCl and $MgSO_4$) are merged and thoroughly washed with MTBE. After solvent evaporation, the crude product is obtained as thick pale-yellow oil. The residue was distilled under vacuum at 2.6 x 10^{-1} mbar (no Vigreux column and short bridge to prevent product from decomposition at high temperature, distillation bridge and flask wrapped in tin foil and paper towels for insulation) to provide the title compound **4** (25.8 g, 134 mmol, 66%) as a viscous pale-yellow oil.

R_f (CH_2Cl_2/MeOH 60:40): 0.14 (concentration dependent, $KMnO_4$ stain).

^{1}H NMR (600 MHz, $CDCl_3$) δ 3.70 – 3.66 (m, 4H, 1-H, 1'-H), 3.61 – 3.57 (m, 4H, 3-H, 3'-H), 3.58 – 3.54 (m, 4H, 2-H, 2'-H), 2.84 – 2.79 (m, 4H, 4-H, 4'-H).

^{13}C NMR (151 MHz, $CDCl_3$) δ 72.8 (C-2, C-2'), 69.9 (C-3, C-3'), 61.7 (C-1, C-1'), 49.1 (C-4, C-4').

Bp 170−173 °C (0.26 mbar).

FT-IR (cm^{-1}, neat): 3291, 2858, 1653, 1454, 1352, 1281, 1248, 1115, 1066, 945, 887, 836, 659, 527, 459.

HRMS $[M+H]^+$ calcd. for $C_8H_{20}NO_4$ 194.1387, found 194.1387.

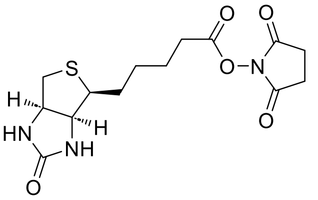

2,5-Dioxopyrrolidin-1-yl 5-((3a*S*,4*S*,6a*R*)-2-oxohexahydro-1*H*-thieno[3,4-*d*]imidazol-4-yl)pentanoate[4] (3, CAS 35013-72-0)

In a 100 mL Schlenk flask under N_2 atmosphere, D-(+)-biotin (**2**) (1.00 g, 4.09 mmol, 1.00 equiv.) and fresh disuccinimidyl carbonate (1.36 g, 5.32 mmol, 1.30 equiv.) were suspended in extra dry DMF (18.4 mL, 4.5 mL/mmol biotin). Triethylamine (2.28 mL, 16.4 mmol, 4.00 equiv.) was added and the resulting suspension was stirred at 19 °C, giving a clear solution after a few minutes. After approximately 60 min, a white solid started to precipitate and stirring was continued for a total reaction time of 25 h. The solvent was removed under reduced pressure and the resulting solid was triturated in diethyl ether. Filtration over a fritted glass funnel (pore size 4) and subsequent washing with EtOAc and Et_2O removed off-white impurities and left a fine white powder (repeated twice). Drying under high vacuum provided the biotin-NHS ester **3** (1.33 g, 3.90 mmol, 95%) as a white solid.

R_f (CH_2Cl_2/MeOH 90:10): 0.44 ($KMnO_4$ stain).

^{1}H NMR (600 MHz, DMSO-*d*$_6$) δ 6.42 (s, 1H, 1-H), 6.36 (s, 1H, 3-H), 4.31 (dd, *J* = 7.7, 5.1 Hz, 1H, 6a-H), 4.15 (ddd, *J* = 7.4, 4.5, 1.8 Hz, 1H, 3a-H), 3.11 (ddd, *J* = 8.3, 6.4, 4.4 Hz, 1H, 4-H), 2.86 – 2.81 (dd, 1H, 6"-H, overlap with 13-H and 14-H), 2.81 (s, 4H, 13-H, 14-H, overlap with 6"-H), 2.69 – 2.64 (m, 2H, 10-H), 2.58 (d, *J* = 12.4 Hz, 1H, 6‘-H), 1.69 – 1.60 (m, 3H, 7"-H, 9-H), 1.50 (tt, *J* = 13.9, 7.1 Hz, 1H, 7'-H), 1.42 (pt, *J* = 8.1, 3.7 Hz, 2H, 8-H).

^{13}C NMR (151 MHz, DMSO-*d*$_6$) δ 170.3 (C-12, C-15), 168.9 (C-11), 162.7 (C-2), 61.0 (C-3a), 59.2 (C-6a), 55.2 (C-4), 39.9 (C-6), 30.0 (C-10), 27.8 (C-7), 27.6 (C-8), 25.4 (C-13, C-14), 24.3 (C-9).

FT-IR (cm^{-1}, neat): 3346, 3230, 3066, 2942, 2918, 2875, 1818, 1788, 1745, 1728, 1697, 1465, 1369, 1278, 1209, 1167, 1110, 1070, 1061, 1048, 993, 917, 884, 860, 834, 813, 739, 701, 655, 606, 582, 549, 492, 486.

T_M 206.4–212.9 °C.

HRMS $[M+H]^+$ calcd. for $C_{14}H_{20}N_3O_5S$ 342.1118, found 342.1119.

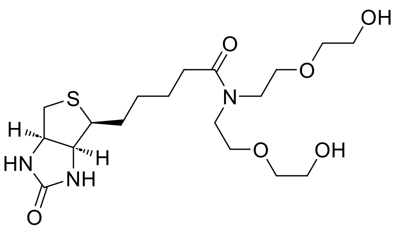

N,N-Bis(2-(2-hydroxyethoxy)ethyl)-5-((3aS,4S,6aR)-2-oxohexahydro-1H-thieno[3,4-d]imidazol-4-yl)pentanamide (7, CAS 2100306-75-8)

Under N_2 atmosphere, a 100 mL round bottom flask was charged with biotin-NHS ester **3** (600 mg, 1.76 mmol, 1.00 equiv.) and amino alcohol **4** (408 mg, 2.11 mmol, 1.20 equiv.). The flask was subjected to three cycles of vacuum/nitrogen backfill before addition of extra dry DMF (35.4 mL, 20.1 mL/mmol NHS ester). Stirring for 15 min rendered a colorless solution, to which triethylamine (735 µL, 5.27 mmol, 3.00 equiv.) was added and, after stirring for 2 h, the solution was heated to 60 °C for 2 h using a heating mantle. The reaction was concentrated *in vacuo* by rotary evaporation (water bath set to 60 °C) to leave a thick colorless oil. A short filter column of neutral aluminium oxide (8 cm) with CH_2Cl_2/MeOH (70:30, 400 mL) was used to efficiently remove released NHS (remains on the baseline of alox TLC plates). Combined fractions were then applied to a column of silica and the product eluted with CH_2Cl_2/MeOH (80:20) to yield the desired biotin-bis-PEG$_2$-alcohol **7** as a colorless sticky oil (570 mg, 1.36 mmol, 77%) after solvent evaporation, which is only soluble in MeOH, DMSO, and DMF.

R_f (silica, CH_2Cl_2/MeOH 70:30): 0.57 ($KMnO_4$ stain).
R_f (neutral aluminium oxide, CH_2Cl_2/MeOH 90:10): 0.23 ($KMnO_4$ stain).

^{1}H NMR (600 MHz, MeOD) δ 4.49 (ddd, *J* = 7.9, 5.0, 0.9 Hz, 1H, 6a-H), 4.31 (dd, *J* = 7.9, 4.4 Hz, 1H, 3a-H), 3.68 – 3.63 (m, 8H, 15-H, 16-H, 17-H, 19-H), 3.63 – 3.58 (m, 4H, 12-H, 13-H), 3.55 (dd, *J* = 5.5, 4.3 Hz, 2H, 18-H), 3.52 (dd, *J* = 5.5, 4.2 Hz, 2H, 14-H), 3.22 (ddd, *J* = 8.8, 6.0, 4.4 Hz, 1H, 4-H), 2.93 (dd, *J* = 12.7, 5.0 Hz, 1H, 6''-H), 2.71 (d, *J* = 12.7 Hz, 1H, 6'-H), 2.53 – 2.45 (m, 2H, 10-H), 1.75 (ddt, *J* = 13.0, 9.2, 6.3 Hz, 1H, 7''-H), 1.70 – 1.57 (m, 3H, 7'-H, 9-H), 1.50 – 1.42 (m, 2H, 8-H).

^{13}C NMR (151 MHz, MeOD) δ 176.2 (C-11), 166.1 (C-2), 73.8 (C-18), 73.6 (C-14), 70.3 (C-17), 70.1 (C-13), 63.3 (C-3a), 62.2 (C-19), 62.2 (C-15), 61.6 (C-6a), 57.1 (C-4), 50.0 (C-16), 47.3 (C-12), 41.1 (C-6), 33.7 (C-10), 29.9 (C-8), 29.6 (C-7), 26.4 (C-9).

FT-IR (cm^{-1}, neat): 3280, 2923, 2863, 1689, 1617, 1456, 1426, 1354, 1330, 1265, 1219, 1118, 1058, 886, 761, 725, 684, 595, 539.

HRMS $[M+H]^+$ calcd. for $C_{18}H_{34}N_3O_6S$ 420.2163, found 420.2166.

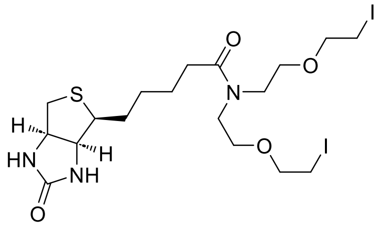

***N,N*-Bis(2-(2-iodoethoxy)ethyl)-5-((3a*S*,4*S*,6a*R*)-2-oxohexahydro-1*H*-thieno[3,4-*d*]imidazol-4-yl)pentanamide (8)**

In a 50 mL Schlenk flask under N_2 atmosphere, bis-hydroxy-functionalized biotin **7** (505 mg, 1.20 mmol, 1.00 equiv.) was dissolved in extra dry DMF (10.8 mL). In a separate Schlenk flask, a solution of methyltriphenoxyphosphonium iodide (2.18 g, 4.82 mmol, 4.00 equiv.) in extra dry DMF (19.3 mL, 250 mM) was added to the biotin mixture and the solution was stirred at 21 °C for 14 h to give a clear colorless solution. Then isopropyl alcohol (921 µL, 12.0 mmol, 10.0 equiv.) was added to quench excess of halogenating reagent. The solution was stirred at 21 °C for 1 h, the reaction mixture transferred with MeOH to a round bottom flask and then the solvent removed by rotary evaporation (water bath set to 60 °C) to give a colorless oil. The residue was purified on silica with CH_2Cl_2/MeOH (98:2, then 93:7) to yield the biotin-bis-PEG-iodide **8** as a colorless viscous oil (544 mg, 850 µmol, 71%).

R_f (CH_2Cl_2/MeOH 90:10): 0.49 ($KMnO_4$ stain and weak UV activity).

^{1}H NMR (600 MHz, $CDCl_3$) δ 5.72 (s, 1H, 3-H), 5.29 (s, 1H, 1-H), 4.51 (dd, *J* = 7.8, 4.9 Hz, 1H, 6a-H), 4.32 (ddd, *J* = 7.8, 4.6, 1.5 Hz, 1H, 3a-H), 3.70 (dq, *J* = 12.3, 6.3, 5.8 Hz, 4H, 14-H, 18-H), 3.66 – 3.60 (m, 6H, 16-H, 13-H, 17-H), 3.57 (q, *J* = 5.0 Hz, 2H, 12-H), 3.24 (t, *J* = 6.4 Hz, 4H, 15-H, 19-H), 3.18 (td, *J* = 7.4, 4.5 Hz, 1H, 4-H), 2.91 (dd, *J* = 12.8, 5.0 Hz, 1H, 6"-H), 2.74 (d, *J* = 12.8 Hz, 1H, 6'-H), 2.50 – 2.38 (m, 2H, 10-H), 1.75 (ddd, *J* = 13.5, 6.8, 2.0 Hz, 1H, 7"-H), 1.68 (hept, *J* = 7.2 Hz, 3H, 7'-H, 9-H), 1.51 – 1.43 (m, 2H, 8-H).

^{13}C NMR (151 MHz, $CDCl_3$) δ 173.7 (C-11), 163.5 (C-2), 71.8 (C-18), 71.5 (C-14), 69.5 (C-17), 69.1 (C-13), 62.0 (C-3a), 60.3 (C-6a), 55.5 (C-4), 49.2 (C-16), 46.5 (C-12), 40.8 (C-6), 32.7 (C-10), 28.5 (C-8), 28.5 (C-7), 25.2 (C-9), 3.7 (C-19), 3.1 (C-15).

FT-IR (cm^{-1}, neat): 3384, 3283, 2921, 2861, 2405, 1684, 1629, 1446, 1422, 1355, 1337, 1314, 1299, 1263, 1229, 1171, 1120, 993, 933, 864, 760, 721, 681, 663, 612, 592, 565, 535, 521.

HRMS $[M+H]^+$ calcd. for $C_{18}H_{32}I_2N_3O_4S$ 640.0197, found 640.0207.

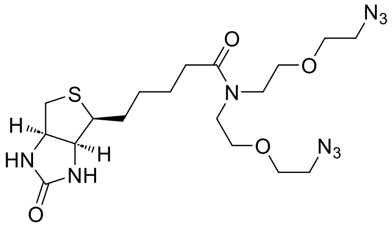

***N*,*N*-Bis(2-(2-azidoethoxy)ethyl)-5-((3a*S*,4*S*,6a*R*)-2-oxohexahydro-1*H*-thieno[3,4-*d*]imidazol-4-yl)pentanamide (9)**

In a 25 mL round bottom flask under N_2 atmosphere, bis-iodinated biotin **8** (349 mg, 546 µmol, 1.00 equiv.) was dissolved in extra dry DMF (3.27 mL, 6.0 mL/mmol bis-iodide). Sodium azide (142 mg, 2.18 mmol, 4.00 equiv.) was added in a single batch and the pale-yellow suspension stirred for 2 h at 19 °C. The solution had become colorless and a white solid had precipitated. Heating at 50 °C was then continued for 14 h using a heating mantle. The reaction was diluted with ice water (50 mL) and extracted with EtOAc (5 x 30 mL). Merged organic phases were washed with cold water (2 x 50 mL) and brine (1 x 50 mL), dried over $MgSO_4$, and filtered over degreased cotton wool. After solvent evaporation, the colorless oil was purified on silica with CH_2Cl_2/MeOH (95:5) to yield the biotin-bis-PEG-azide **9** (222 mg, 472 µmol, 87%) as a hard colorless glass.

R_f (CH_2Cl_2/MeOH 90:10): 0.44 (CAM stain).

^{1}H NMR (600 MHz, $CDCl_3$) δ 5.52 (s, 1H, 3-H), 5.04 (s, 1H, 1-H), 4.44 (ddt, *J* = 7.7, 5.0, 1.2 Hz, 1H, 6a-H), 4.25 (ddd, *J* = 7.8, 4.6, 1.5 Hz, 1H, 3a-H), 3.60 – 3.54 (m, 10H, 13-H, 14-H, 16-H, 17-H, 18-H), 3.52 (dd, *J* = 5.5, 4.0 Hz, 2H, 12-H), 3.34 – 3.25 (m, 4H, 15-H, 19-H), 3.11 (td, *J* = 7.4, 4.6 Hz, 1H, 4-H), 2.85 (dd, *J* = 12.8, 5.0 Hz, 1H, 6''-H), 2.67 (d, *J* = 12.8 Hz, 1H, 6'-H), 2.36 (td, *J* = 7.3, 2.0 Hz, 2H, 10-H), 1.71 – 1.63 (m, 1H, 7''-H), 1.65 – 1.57 (m, 3H, 7'-H, 9-H), 1.41 (dtt, *J* = 13.7, 8.9, 4.9 Hz, 2H, 8-H).

^{13}C NMR (151 MHz, $CDCl_3$) δ 173.6 (C-11), 163.4 (C-2), 70.3, 70.0, 69.9, 69.6, 61.9 (C-3a), 60.2 (C-6a), 55.4 (C-4), 50.9 (C-15, C-19), 49.1 (C-16), 46.5 (C-12), 40.7 (C-6), 32.6 (C-10), 28.4 (C-8), 28.4 (C-7), 25.1 (C-9).

FT-IR (cm^{-1}, neat): 3317, 3240, 2930, 2869, 2103 (N_3), 1704, 1667, 1621, 1459, 1424, 1367, 1344, 1287, 1272, 1249, 1235, 1201, 1164, 1116, 1063, 1009, 937, 898, 859, 846, 813, 727, 643, 620, 598, 541, 502, 491, 456.

HRMS $[M+H]^+$ calcd. for $C_{18}H_{32}N_9O_4S$ 470.2293, found 470.2301.

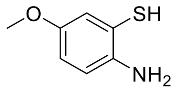

2-Amino-5-methoxybenzenethiol (12, CAS 6274-29-9)

In a 1 L round bottom flask, 2-amino-6-methoxy-benzothiazole (**11**) (35.0 g, 194 mmol, 1.00 equiv.; 149 USD or 160 EUR per 500 grams at TCI) is suspended in 485 mL 40% NaOH in water (194 g, 4.85 mol, 25.0 equiv.), ethylene glycol (43.4 mL, 0.78 mmol, 4.00 equiv.) is added and the mixture subsequently degassed by freeze-pump-thaw (2 cycles). The suspension is refluxed at 140 °C for 39 h using a heating mantle (remains a white-grey suspension). While cooling to 0 °C in an ice bath, the reaction mixture turned purple and was diluted with ice water (1 L) in a 5 L Erlenmeyer flask to give a red-brown solution. Acidification with concentrated HCl to pH 2–3 shifts the color from red-brown to green and then yellow, before a bright-yellow solid precipitates (total HCl amount is 440 mL). Neutralization with 20% K_2CO_3 (pH 5–6) is followed by repeated extraction with MTBE (8 x 200 mL) during which the aqueous phase remained turbid and a green solid precipitated. Merged organic phases are washed with brine (500 mL) and dried over $MgSO_4$. After solvent evaporation, the product **12** is obtained as an orange oil and crystallizes at room temperature to give a yellow solid (23.0 g, 148 mmol, 76%).

R_f (PE/EtOAc 20:80): 0.66 (UV and $KMnO_4$ stain).

^1H NMR (600 MHz, $CDCl_3$) δ 6.80 (dd, *J* = 8.8, 2.9 Hz, 1H, 6-H), 6.71 – 6.66 (m, 2H, 3-H, 4-H), 4.06 (s, 2H, 8-H), 3.60 (s, 3H, 10-H).

^{13}C NMR (151 MHz, $CDCl_3$) δ 152.0 (C-5), 142.8 (C-2), 120.2 (C-3), 119.5 (C-6), 119.4 (C-1), 116.8 (C-4), 55.9 (C-10).

FT-IR (cm^{-1}, neat): 3277, 2909, 2884, 2866, 2850, 2803, 1605, 1563, 1557, 1528, 1490, 1449, 1427, 1409, 1360, 1348, 1320, 1287, 1259, 1220, 1187, 1169, 1136, 1120, 1097, 1065, 1040, 1004, 966, 942, 882, 844, 835, 816, 752, 724, 702, 687, 678, 653, 626, 609, 590, 563, 554, 540, 514, 485, 475.

T_M 70.3–73.8 °C.

HRMS $[M+H]^+$ calcd. for $C_7H_{10}NOS$ 156.0478, found 156.0477.

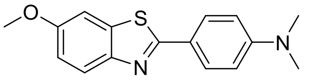

4-(6-Methoxybenzo[*d*]thiazol-2-yl)-*N,N*-dimethylaniline (14, CAS 10205-71-7)

In a 100 mL round bottom flask under N_2 atmosphere, 4-(dimethylamino)benzaldehyde (**13**) (3.30 g, 2.12 mmol, 1.00 equiv.) and 2-aminothiophenol (**12**) (4.46 g, 28.8 mmol, 1.30 equiv.) in 44.2 mL DMSO (2.0 mL/mmol aldehyde) were stirred at room temperature until fully dissolved. The deep orange solution was stirred at 125 °C for 2 h using a heating mantle to form a black-yellow colored solution. The septum was removed and stirring under air continued for 30 min while heating. The dark brown solution was diluted with water (150 mL) to precipitate a greenish solid. Further dilution with water and suction filtration provided a solid, which was further washed with water and distilled EtOH and finally dried under vacuum to give the product **14** (5.19 g, 18.2 mmol, 82%) as an amorphous green solid. The product was used without further purification.

R_f (CH_2Cl_2/THF 90:10): 0.73 (UV).

^{1}H NMR (600 MHz, $CDCl_3$) δ 7.92 (d, *J* = 8.5 Hz, 2H, 9-H, 13-H), 7.88 (d, *J* = 8.9 Hz, 1H, 7-H), 7.31 (d, *J* = 2.5 Hz, 1H, 4-H), 7.04 (dd, *J* = 8.9, 2.5 Hz, 1H, 5-H), 6.74 (d, *J* = 8.6 Hz, 2H, 10-H, 12-H), 3.88 (s, 3H, 16-H), 3.05 (s, 6H, 14-H, 15-H).

^{13}C NMR (151 MHz, $CDCl_3$) δ 166.7 (C-2), 157.3 (C-6), 152.1 (C-11), 148.7 (C-3a), 135.7 (C-7a), 128.7 (C-9, C-13), 122.8 (C-7), 121.6 (C-8), 115.1 (C-5), 111.9 (C-10, C-12), 104.5 (C-4), 55.9 (C-16), 40.4 (C-14, C-15).

FT-IR (cm^{-1}, neat): 3011, 2966, 2935, 2906, 2865, 2834, 2805, 1604, 1557, 1530, 1487, 1461, 1444, 1429, 1411, 1366, 1318, 1284, 1260, 1241, 1222, 1187, 1167, 1116, 1057, 1022, 1005, 967, 958, 945, 891, 832, 814, 806, 751, 732, 722, 694, 686, 626, 591, 547, 510, 484.

T_M 176.5–178.0 °C.

HRMS $[M+H]^+$ calcd. for $C_{16}H_{17}N_2OS$ 285.1056, found 285.1058.

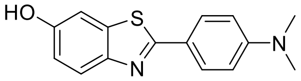

2-(4-(Dimethylamino)phenyl)benzo[*d*]thiazol-6-ol (10, CAS 566169-94-6)

<u>Procedure A from commercially available Pittsburgh Compound B (1):</u> In a 100 mL Schlenk flask under N_2 atmosphere, Pittsburgh B (**1**) (250 mg, 975 µmol, 1.00 equiv.; 985 USD or 1025 EUR per gram at BLDpharm) and paraformaldehyde (158 mg, 5.27 mmol, 5.40 equiv.) were three times vacuum/N_2 backfilled. The compounds were suspended in dry THF (24.9 mL, 25.5 mL/mmol PiB) and stirred for 15 min at 19 °C to give a milky beige suspension. The solution was cooled to 0 °C and sodium cyanoborohydride (331 mg, 5.27 mmol, 5.40 equiv.) added in one portion. At 0 °C, glacial acetic acid (301 µL, 5.27 mmol, 5.40 equiv.) was added dropwise to the THF solution. Stirring was continued for 25 min at 0 °C, then 25 min at 19 °C and finally for 13 h at 50 °C using a heating mantle. The reaction was quenched with ice water (20 mL) and subsequently extracted with CH_2Cl_2 (3 x 50 mL). THF (stabilizer-free) was added during the extractive workup to assist phase separation and the transfer of the bright yellow fluorescent solution into the organic phase. Organic phases were combined and washed with saturated $NaHCO_3$ (2 x 50 mL) to give a colorless extract, followed by brine (1 x 50 mL). Drying over $MgSO_4$ and filtration over degreased cotton wool provided an ivory extract. The solvent was removed under reduced pressure and a fine bright yellow solid obtained. The solid was suspended in diisopropyl ether/EtOAc (1:1) and filtered with a fritted funnel (pore size 4). The yellow powder was washed with diisopropyl ether /EtOAc (1:1) and *i*PrOH. The recovered Me-PiB **10** was obtained as a bright yellow powder and the procedure repeated once to yield the title compound (255 mg, 942 µmol, 97%).

R_f (CH_2Cl_2/THF 90:10): 0.59 (UV and CAM stain).

^{1}H NMR (600 MHz, DMSO-d_6) δ 9.71 (s, 1H, OH), 7.80 (d, *J* = 8.4 Hz, 2H, 9, 13-H), 7.72 (d, *J* = 8.7 Hz, 1H, 4-H), 7.33 (d, *J* = 2.5 Hz, 1H, 7-H), 6.92 (dd, *J* = 8.7, 2.4 Hz, 1H, 5-H), 6.80 (d, *J* = 8.4 Hz, 2H, 10-H, 12-H), 3.00 (s, 6H, 14-H, 15-H).

^{13}C NMR (151 MHz, DMSO-d_6) δ 164.2 (C-2), 155.0 (C-6), 151.8 (C-11), 147.4 (C-3a), 135.2 (C-7a), 127.9 (C-9, C-13), 122.4 (C-4), 120.6 (C-8), 115.5 (C-5), 111.8 (C-10, C-12), 106.7 (C-7), 39.7 (C-14, C-15).

FT-IR (cm^{-1}, neat): 3065, 3012, 2892, 2804, <u>2762, 2675, 2594, 2547, 2458,</u> 1605, 1558, 1474, 1427, 1410, 1346, 1278, 1240, 1207, 1194, 1168, 1126, 1052, 1004, 972, 946, 906, 838, 807, 710, 697, 625, 589, 546, 509, 503. [The underlined peaks belong to the characteristic very broad vibrational OH stretching (3100–2400 cm^{-1}) and are the only signals, which significantly differ from compound **14**].

T_M 287.3–294.9 °C (Decomposition).

HRMS $[M+H]^+$ calcd. for $C_{15}H_{15}N_2OS$ 271.0900, found 271.0903.

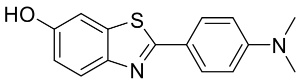

2-(4-(Dimethylamino)phenyl)benzo[*d*]thiazol-6-ol (10, CAS 566169-94-6)

<u>Procedure B[5,6] from compound 14:</u> In a 500 mL Schlenk flask under N_2 atmosphere, methoxy-protected Me-PiB **14** (5.00 g, 17.6 mmol, 1.00 equiv.) is dissolved in dry CH_2Cl_2 (175 mL, 10 mL/mmol PiB derivative) and the brown-purple solution cooled to 0 °C. BBr_3 (5.01 mL, 52.8 mmol, 3.00 equiv.) is slowly added at 0 °C. After completed addition, a greenish solid precipitates and stirring is continued for 30 min at 0 °C. The suspension is allowed to warm to 27 °C and stirring continued for 17 h during which time, a dark film had solidified on the surface of the flask. The reaction is cooled to 0 °C and quenched by the addition of water (150 mL) to give an orange suspension. The solid was scraped from the surface and the pH adjusted with 3 M NaOH to approx. 12–14. The resulting green slurry is filtered and the green solid washed with water and Et_2O (wash fraction is orange due to HBr) to give the demethylated Me-PiB **10** (3.86 g, 14.3 mmol, 81%) as a green amorphous solid after solvent removal and drying under high vacuum.

R_f (CH_2Cl_2/THF 90:10): 0.52 (UV and CAM stain).

^{1}H NMR (600 MHz, DMSO-d_6) δ 9.73 (s, 1H, OH), 7.79 (d, J = 8.9 Hz, 2H, 9-H, 13-H), 7.73 (d, J = 8.7 Hz, 1H, 4-H), 7.33 (d, J = 2.5 Hz, 1H, 7-H), 6.92 (dd, J = 8.7, 2.5 Hz, 1H, 5-H), 6.79 (d, J = 8.8 Hz, 2H, 10-H, 12-H), 2.99 (s, 5H, 14-H, 15-H).

^{13}C NMR (151 MHz, DMSO-d_6) δ 164.2 (C-2), 155.0 (C-6), 151.7 (C-11), 147.3 (C-3a), 135.2 (C-7a), 127.9 (C-9, C-13), 122.4 (C-4), 120.6 (C-8), 115.5 (C-5), 111.9 (C-10, C-12), 106.7 (C-7), 39.7 (C-14, C-15).

FT-IR (cm^{-1}, neat): 3067, 3014, 2893, 2808, <u>2767, 2678, 2595, 2552, 2460,</u> 1606, 1558, 1476, 1427, 1411, 1347, 1279, 1240, 1207, 1195, 1170, 1126, 1053, 1005, 972, 946, 907, 836, 808, 755, 710, 698, 626, 589, 547, 510, 503. The underlined peaks belong to the characteristic very broad vibrational OH stretching (3100–2400 cm^{-1}) and are the only signals, which significantly differ from compound **14**].

T_M 286.9–291.9 °C.

HRMS [M+H]$^+$ calcd. for $C_{15}H_{15}N_2OS$ 271.0900, found 271.0902.

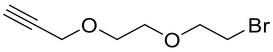

3-(2-(2-Bromoethoxy)ethoxy)prop-1-yne (16, CAS 1287660-82-5)

In a 100 mL Schlenk flask under N_2 atmosphere, a solution of alcohol **15** (1.07 g, 7.42 mmol, 1.00 equiv.) and carbon tetrabromide (2.95 g, 8.91 mmol, 1.20 equiv.) in dry CH_2Cl_2 (61.8 mL, 120 mM) was prepared. A solution of PPh_3 (2.34 g, 8.91 mmol, 1.20 equiv.) in dry CH_2Cl_2 (10 mL) was added to the pale yellow CBr_4 solution at ambient temperature and the reaction mixture, which had quickly turned yellow, was stirred for 72 h at 19 °C. The solvent was removed under reduced pressure to yield the crude product as brown oil. The oil was dissolved in CH_2Cl_2 and immobilized on celite (PPh_3 and PPh_3=O stick to celite) by solvent removal. Purification was then performed by chromatography on silica with PE/EtOAc (80:20) to give the brominated alkyne **16** (1.52 g, 7.34 mmol, 99%) as a colorless liquid with characteristic odor in near-quantitative yield.

R_f (PE/EE 80:20): 0.39 ($KMnO_4$ stain).

^1H NMR (600 MHz, $CDCl_3$) δ 4.22 (d, J = 2.4 Hz, 2H, 3-H), 3.82 (t, J = 6.4 Hz, 2H, 6-H), 3.74 – 3.67 (m, 4H, 4-H, 5-H), 3.48 (t, J = 6.4 Hz, 2H, 7-H), 2.44 (t, J = 2.3 Hz, 1H, 1-H).

^{13}C NMR (151 MHz, $CDCl_3$) δ 79.7 (C-2), 74.8 (C-1), 71.4 (C-6), 70.5 (C-5), 69.2 (C-4), 58.6 (C-3), 30.3 (C-7).

FT-IR (cm^{-1}, neat): 3291, 3019, 2866, 2115, 1459, 1442, 1423, 1352, 1277, 1246, 1227, 1186, 1100, 1041, 1008, 952, 920, 881, 841, 668, 573, 512.

HRMS $[M+Na]^+$ calcd. for $C_7H_{11}BrNaO_2$ 228.9835, found 228.9829.

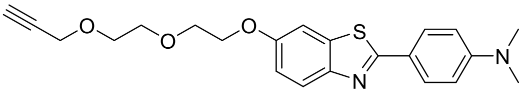

***N*,*N*-Dimethyl-4-(6-(2-(2-(prop-2-yn-1-yloxy)ethoxy)ethoxy)benzo[*d*]thiazol-2-yl)aniline (17)**

To a 100 mL flask under N_2 atmosphere, Me-PiB **10** (86.5 mg, 320 µmol, 1.00 equiv.), alkyne bromide **16** (99.4 mg, 480 µmol, 1.50 equiv.), and ground potassium hydroxide (53.9 mg, 960 µmol, 3.00 equiv.) are added and suspended in dry glyme (43.2 mL, 0.5 mL/mg PiB). The reaction is vigorously stirred at 80 °C for 20 h using a heating mantle, giving a brown dispersion. After cooling to room temperature, the solvent was removed and the green-brown residue diluted with water (150 mL) and CH_2Cl_2 (400 mL). As the organic phase was very turbid, EtOH was added to clear the organic phase and allow phase separation. Phases were separated and the aqueous phase was extracted with CH_2Cl_2 (3 x 150 mL, EtOH added if needed). Merged organic phases were washed with saturated $NaHCO_3$ (250 mL) and dried over $MgSO_4$. After filtration and subsequent evaporation of solvent, the crude product was yielded as an olive green solid. Purification on silica with PE/CH_2Cl_2/MeOH (40:59:1) provided a beige-brown solid that contained aliphatic impurities of unknown origin. The solid was suspended in *i*PrOH and applied to a pipet filled silica column. The column was thoroughly washed with *i*PrOH, giving a yellow eluate that contained minor product and all undesired aliphatic impurities. The product remained as beige solid on top of the silica and was eluted stepwise with CH_2Cl_2/MeOH (1:1), CH_2Cl_2, and then MeOH. The process was repeated with the *i*PrOH fraction to increase the yield. Although near-full conversion was reported from TLC, the title compound was obtained as an amorphous beige-yellow solid (66.5 mg, 168 µmol, 52%) in a moderate yield.

R_f (CH_2Cl_2/THF 90:10): 0.68 (UV and CAM stain).

^{1}H NMR (600 MHz, $CDCl_3$) δ 7.90 (d, J = 9.0 Hz, 2H, 9-H, 13-H), 7.85 (d, *J* = 8.9 Hz, 1H, 4-H), 7.34 (d, *J* = 2.5 Hz, 1H, 7-H), 7.06 (dd, *J* = 8.9, 2.5 Hz, 1H, 5-H), 6.74 (d, *J* = 9.0 Hz, 2H, 10-H, 12-H), 4.22 (d, *J* = 2.4 Hz, 2H, 20-H), 4.22 – 4.18 (m, 2H, 16-H), 3.90 (dd, *J* = 5.6, 4.0 Hz, 2H, 17-H), 3.80 – 3.76 (m, 2H, 18-H), 3.76 – 3.72 (m, 2H, 19-H), 3.05 (s, 6H, 14-H, 15-H), 2.43 (t, *J* = 2.4 Hz, 1H, 22-H).

^{13}C NMR (151 MHz, $CDCl_3$) δ 166.7 (C-2), 156.4 (C-6), 152.1 (C-11), 149.3 (C-3a), 135.9 (C-7a), 128.7 (C-9, C-13), 122.9 (C-4), 121.8 (C-8), 115.6 (C-5), 111.9 (C-10, C-12), 105.6 (C-7), 79.8 (C-21), 74.7 (C-22), 70.8 (C-18), 70.0 (C-17), 69.3 (C-19), 68.3 (C-16), 58.6 (C-20), 40.3 (C-14, C-15).

FT-IR (cm^{-1}, neat): 3277, 2909, 2884, 2866, 2850, 2803, 1605, 1563, 1557, 1528, 1490, 1449, 1427, 1409, 1360, 1348, 1320, 1287, 1259, 1220, 1187, 1169, 1136, 1120, 1097, 1065, 1040, 1004, 966, 942, 8 82, 835, 816, 752, 724, 702, 687, 678, 653, 626, 609, 590, 554, 540, 514, 485, 475.

T_M 116.5–120.6 °C.

HRMS [M+H]$^+$ calcd. for $C_{22}H_{25}N_2O_3S$ 397.1580, found 397.1584.

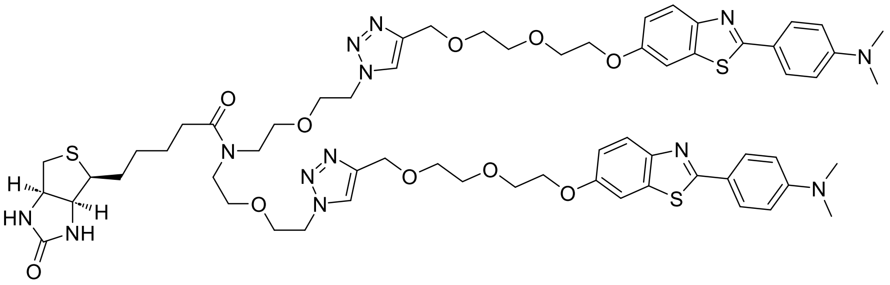

***N*,*N*-Bis(2-(2-(4-((2-(2-((2-(4-(dimethylamino)phenyl)benzo[*d*]thiazol-6-yl)oxy)ethoxy)ethoxy)methyl)-1*H*-1,2,3-triazol-1-yl)ethoxy)ethyl)-5-((3a*S*,4*S*,6a*R*)-2-oxohexahydro-1*H*-thieno[3,4-*d*]imidazol-4-yl)pentanamide (18, cCAP-1)**

To a 100 mL round bottom flask under N_2 atmosphere, biotin bis-azide **9** (34.1 mg, 72.6 µmol, 1.00 equiv.), alkynylated Me-PiB **16** (59.0 mg, 149 µmol, 2.05 equiv.), and TDETA[2] (10.3 mg, 21.8 µmol, 0.30 equiv.) are added. The compounds are dissolved in 25.4 mL chloroform (0.35 mL/µmol biotin). $Cu(OAc)_2$ (2.18 mL, 3.96 mg, 21.8 µmol, 0.30 equiv., 10 mM in EtOH) and additional 23.2 mL of EtOH are added to give finally a 1:1 mixture of chloroform/EtOH. An aqueous solution of sodium ascorbate (182 µL, 7.19 mg, 36.3 µmol, 0.50 equiv., 200 mM in water) is then added and the pale-yellow reaction stirred at 19 °C for 23 h. The reaction was concentrated *in vacuo* and the slimy yellow residue taken up in CH_2Cl_2/MeOH (95:5), before applied to a silica column. The column was washed with CH_2Cl_2/MeOH (95:5) and the product then eluted with CH_2Cl_2/MeOH (90:10). A yellow oil is obtained after solvent removal that slowly transformed into a pale-yellow foam as which the desired bis-click product cCAP-1 **18** (80.7 mg, 63.9 µmol, 88%) is finally obtained after extensive drying.

R_f (CH_2Cl_2/MeOH 80:20): 0.76 (UV and yellow spot with CAM stain).

^{1}H NMR (600 MHz, $CDCl_3$) δ 7.88 (d, *J* = 8.8 Hz, 4H, 31-H, 35-H, 57-H, 61-H), 7.83 (d, *J* = 8.9 Hz, 2H, 25-H, 51-H), 7.60 (s, 1H, 42-H), 7.57 (s, 1H, 16-H), 7.35 – 7.29 (m, 2H, 28-H, 54-H), 7.03 (dt, *J* = 8.9, 2.3 Hz, 2H, 24-H, 50-H), 6.72 (d, *J* = 8.8 Hz, 4H, 32-H, 34-H, 58-H, 60-H), 5.78 (s, 1H, 3-H), 4.88 (s, 1H, 1-H), 4.68 (d, *J* = 7.2 Hz, 4H, 18-H, 44-H), 4.46 – 4.39 (m, 5H, 6a-H, 15-H, 41-H), 4.26 (t, *J* = 6.1 Hz, 1H, 3a-H), 4.19 – 4.14 (m, 4H, 22-H, 48-H), 3.86 (q, *J* = 4.1 Hz, 4H, 21-H, 47-H), 3.78 – 3.68 (m, 12H, 14-H, 19-H, 20-H, 40-H, 45-H, 46-H), 3.49 (t, *J* = 5.4 Hz, 2H, 13-H), 3.38 (t, *J* = 5.2 Hz, 4H, 12-H, 39-H), 3.29 (t, *J* = 5.6 Hz, 2H, 38-H), 3.09 (td, *J* = 7.4, 4.6 Hz, 1H, 4-H), 3.03 (s, 12H, 36-H, 37-H, 62-H, 63-H), 2.84 (dd, *J* = 12.8, 5.0 Hz, 1H, 6"-H), 2.66 (d, *J* = 12.8 Hz, 1H, 6'-H), 2.24 (td, *J* = 7.4, 3.1 Hz, 2H, 10-H), 1.68 (ddd, *J* = 16.8, 13.0, 6.7 Hz, 1H, 7'-H), 1.63 – 1.50 (m, 3H, 7"-H, 9-H), 1.38 (tq, *J* = 15.6, 7.5, 7.0 Hz, 2H, 8-H).

^{13}C NMR (151 MHz, $CDCl_3$) δ 173.5 (C-11), 166.78 (C-29 or C-55), 166.75 (C-29 or C-55), 163.2 (C-2), 156.38 (C-23 or C-49), 156.36 (C-23 or C-49), 152.1 (C-33, C-59), 149.23 (C-26 or C-52), 149.21 (C-26 or C-52), 145.3 (C-43), 145.1 (C-17), 135.9 (C-27, C-53), 128.6 (C-31, C-35, C-57, C-61), 123.6 (C-42), 123.5 (C-16), 122.84 (C-25 or C-51), 122.83 (C-25 or C-51), 121.65 (C-30 or C-56), 121.63 (C-30 or C-56), 115.6 (C-50), 115.6 (C-24), 111.9 (C-32, C-34, C-58, C-60), 105.6 (C-28, C-54), 70.9 (C-20, C-46), 70.0 (C-21, C-47), 69.9 (C-19, C-45), 69.5 (C-13), 69.5 (C-40), 69.3 (C-39), 69.1 (C-14), 68.3 (C-22, C-48) 64.8 (C-44), 64.8 (C-18), 61.9 (C-3a), 60.1 (C-6a), 55.5 (C-4), 50.2 (C-15, C-41), 48.6 (C-38), 46.0 (C-12), 40.7 (C-6), 40.3 (C-36, C-37, C-62, C-63), 32.4 (C-10), 28.3 (C-7, C-8), 24.9 (C-9).

FT-IR (cm^{-1}, neat): 3241, 3214, 3135, 3090, 3079, 2906, 2865, 2817, 1701, 1633, 1604, 1560, 1532, 1491, 1445, 1430, 1362, 1313, 1285, 1263, 1223, 1188, 1169, 1122, 1064, 1049, 1007, 966, 941, 884, 817, 772, 751, 725, 712, 702, 687, 628, 607, 589, 556, 517, 484, 461.

T_M 79.5–90.3 °C (the yellow flakes start rapid shrinking at 65 °C and show a broad melting point).

HRMS $[M+H]^+$ calcd. for $C_{62}H_{80}N_{13}O_{10}S_3$ 1262.5308, found 1262.5293.

4. Biochemical procedures

Preparation of α-syn monomers and fibrils

N-terminally acetylated human wild-type α-syn was expressed in *E. coli* BL21 (DE3) carrying the pT7 vector for codon-optimized α-syn and the pNatB vector for the N-terminal acetyltransferase B complex from *Schizosaccharomyces pombe*. Bacteria were cultured in 120 mL lysogeny broth (LB) medium with 100 µg/mL ampicillin and 34 µg/mL chloramphenicol at 37 °C and 120 rpm overnight. The next day, the optical density was measured at 600 nm and the culture was diluted to an optical density of 0.1 in 1 L of LB medium. The culture was then incubated with 100 µg/mL ampicillin and 34 µg/mL chloramphenicol at 37 °C until the optical density reached 1.0–1.2. Expression was induced with 1 mM of IPTG. After 4.5 h, the cells were pelleted at 5000 × g and 4 °C. The pellets were resuspended in 20 mM Tris-HCl (pH 8.0) containing a protease inhibitor (Roche) and were boiled for 2 × 15 min, then centrifuged at 20,000 × g and 4 °C for 30 min. Ammonium precipitation was performed with 0.45 g/mL of $(NH_4)_2SO_4$ crystals, which were added to the supernatant over 5 min and stirred for 15 min. The centrifugation was repeated and the pellet was resuspended in 50 mL of 20 mM Tris-HCl (pH 8.0). α-Syn was purified using the HiPrep QFF 16/10 anion exchange chromatography column and a linear gradient from 20 mM Tris-HCl (pH 8.0) binding buffer to 1 M NaCl in 20 mM Tris-HCl (pH 8.0) elution buffer on an ÄKTA pure chromatography system (GE Healthcare). The ammonium precipitation was repeated and the pellet was resuspended in 50 mM Tris-HCl (pH 7.2) and purified using a HiLoad 16/60 Superdex 75 pg size exclusion column (Cytiva) over 1.5 column volumes. NaCl was added to obtain 50 mM Tris-HCl and 150 mM NaCl. The protein was concentrated to 5 mg/mL using a vivaspin concentrator (Sartorius). The α-syn monomer was fibrillized by incubation at 37 °C and 1000 rpm on a Thermomixer (Eppendorf) for 7 d. Fibrils were sonicated by four 15 s sonification steps with 2 min pauses between each step using a Sonoplus Mini20 (Bandelin) and an MS 1.5 microtip.

Fluorescence measurements

All fluorescence measurements were done in phosphate-buffered saline (PBS) at pH 7.4 and room temperature using 96-well clear bottom plates (Thermo Fisher Scientific) and a CLARIOstar microplate reader (BMG Labtech). For the acquisition of fluorescence spectra, fluorescence excitation (320–415 nm) was measured at an emission wavelength of 440 nm and emission spectra (390–740 nm) at an excitation wavelength of 365 nm. We used 100 µL substrate volumes with 20 µM cCAP-1 (**18**) in the absence or presence of 10 µM (monomer equivalent) α-syn fibrils. For dilution experiments with cCAP-1 (**18**) (0–200 nM) in the presence of 10 µM (monomer equivalent) α-syn fibrils, emission spectra (380–600 nm) were measured at an excitation wavelength of 355 nm in 100 µL.

ELISA

Streptavidin-coated maximum capacity microplates (Biotez) were blocked overnight at 4 °C with 200 µL of blocking buffer consisting of 5% (w/v) non-fat dry milk in PBS. The plates were then washed five times with PBS with 0.1% Tween20 (PBST) and once with PBS. Wells were loaded either with 100 µL of 0.34 µM (5 µg/mL, monomer equivalent) α-syn fibrils only (control) or with 100 µL of 0.34 µM α-syn fibrils that had been previously incubated with 10 µM cCAP-1 (**18**) for 10 min at room temperature. The plates were then incubated for 2 h at room temperature and washed five times with PBST and once with PBS. For detection of α-syn fibrils, the mouse monoclonal anti-α-synuclein Syn211 antibody (Santa Cruz Biotechnology) was diluted 1:1000 in blocking buffer and 100 µL of this solution was added to each well and incubated overnight at 4 °C. Following five washes with PBST and one with PBS, the wells were incubated for 2 h with gentle agitation at room temperature with 100 µL of an anti-mouse horseradish peroxidase-linked secondary antibody (Invitrogen) at a 1:5000 dilution in blocking buffer. After five washes with PBST and one with PBS, the wells were incubated with 100 µL TMB substrate solution (Pierce TMB Substrate Kit, Thermo Fisher Scientific). The color reaction was stopped after 50 min with 2 M sulfuric acid and the absorbance measured at 450 nm with a CLARIOstar microplate reader (BMG Labtech). Statistical analysis was performed using Student's t-test.

5. NMR Spectra

2,2'-((Azanediylbis(ethane-2,1-diyl))bis(oxy))bis(ethan-1-ol) (**4**)

^{1}H NMR (600 MHz, $CDCl_3$)

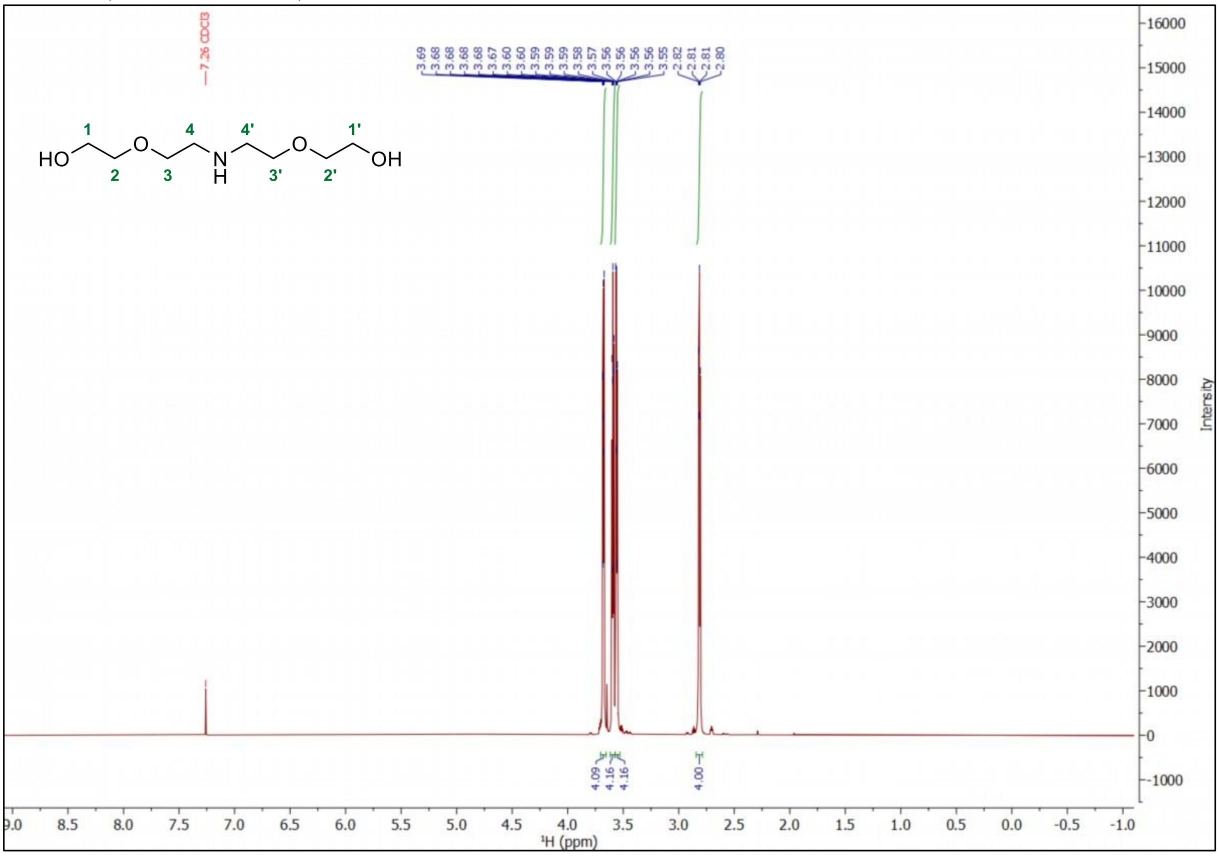

^{13}C NMR (151 MHz, $CDCl_3$)

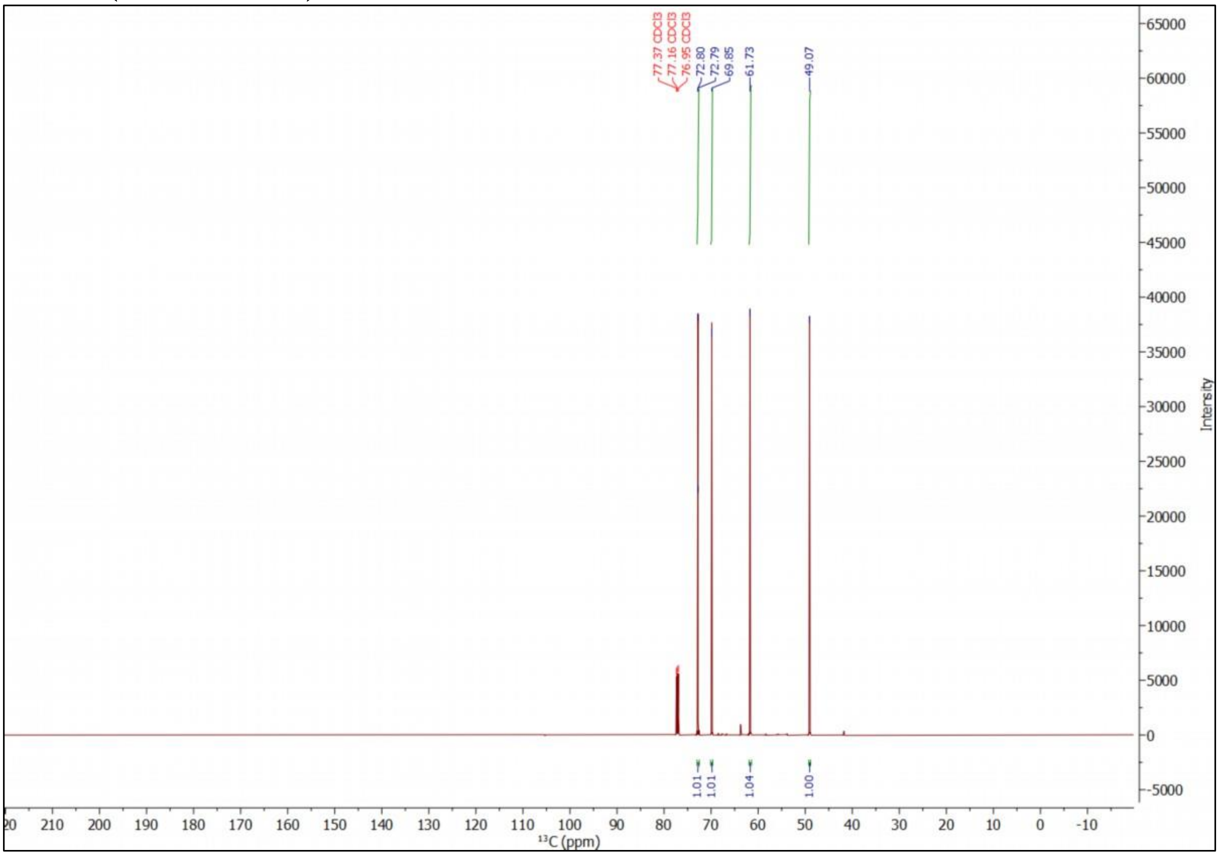

DEPT135 NMR ($CDCl_3$, **4**)

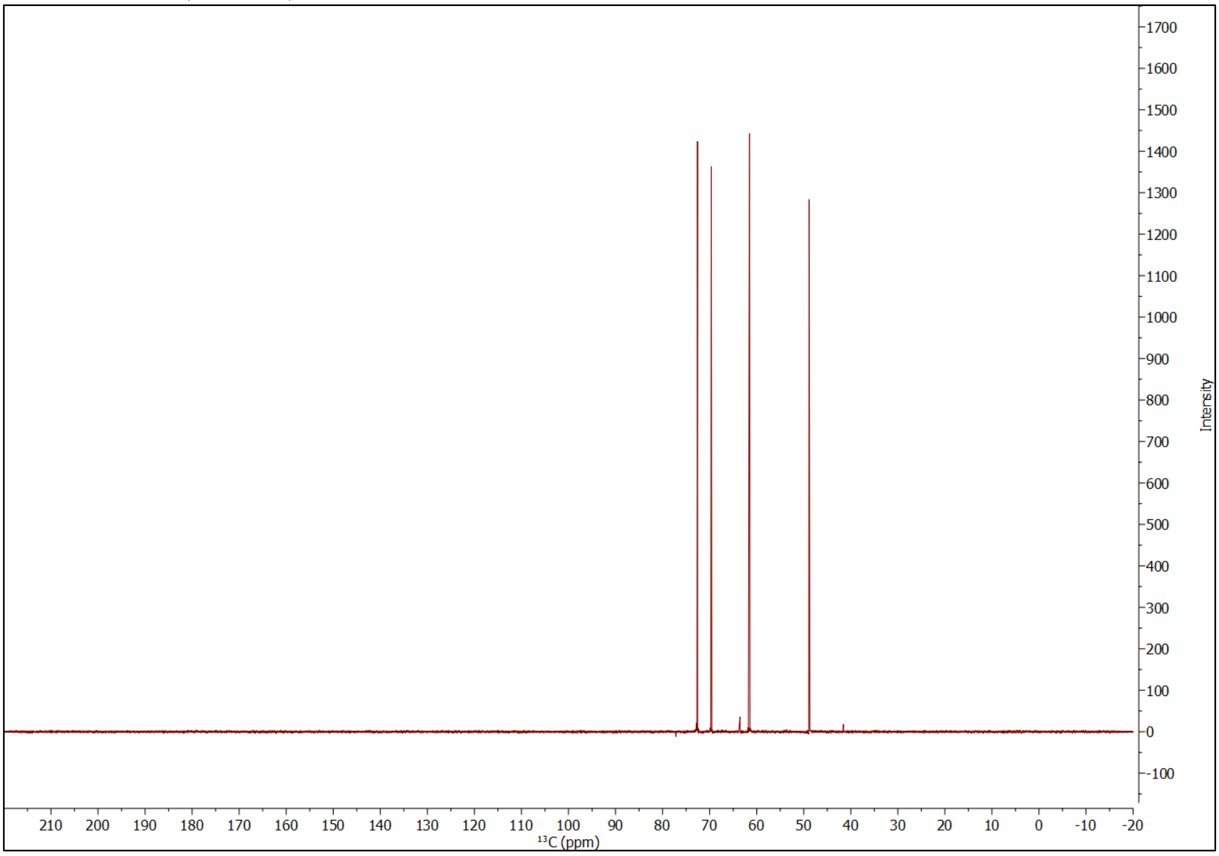

^1H-^1H-COSY NMR ($CDCl_3$, **4**)

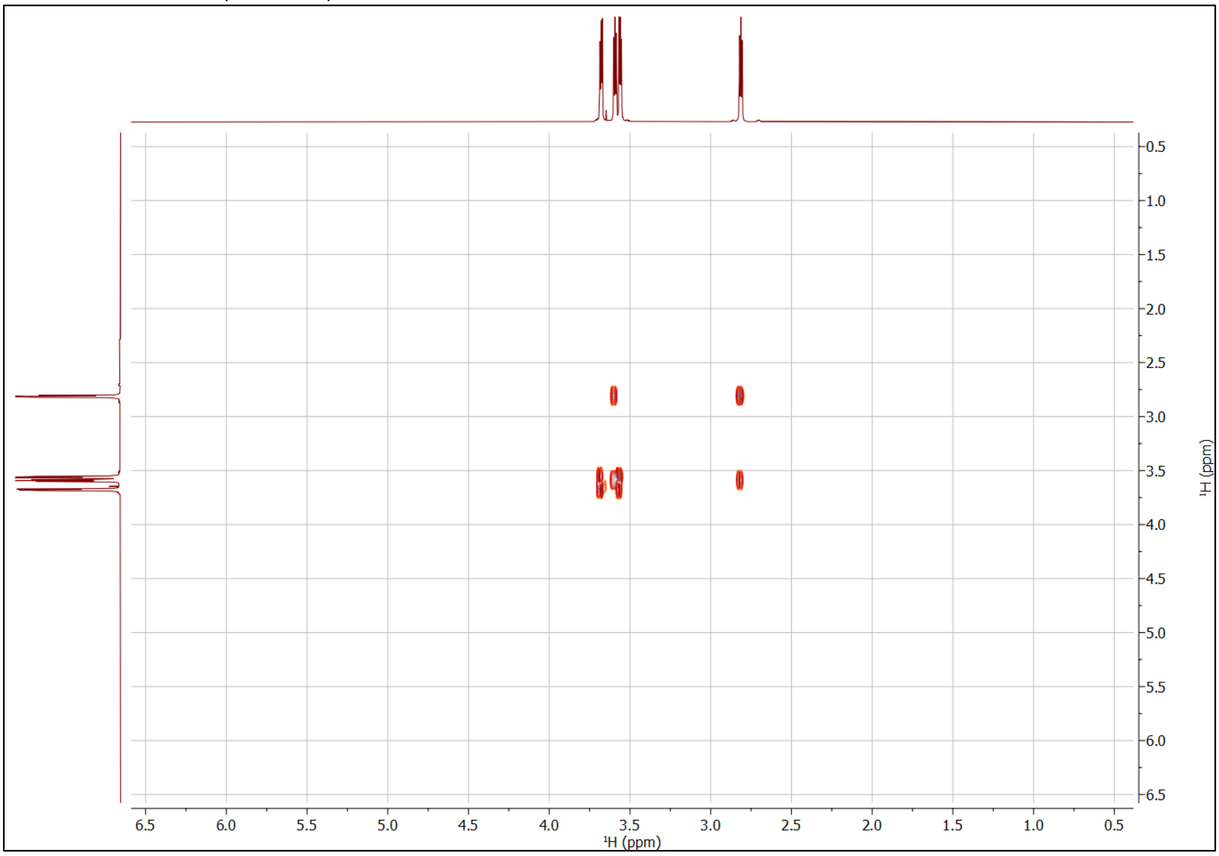

^{1}H-^{13}C-HSQC NMR ($CDCl_3$, **4**)

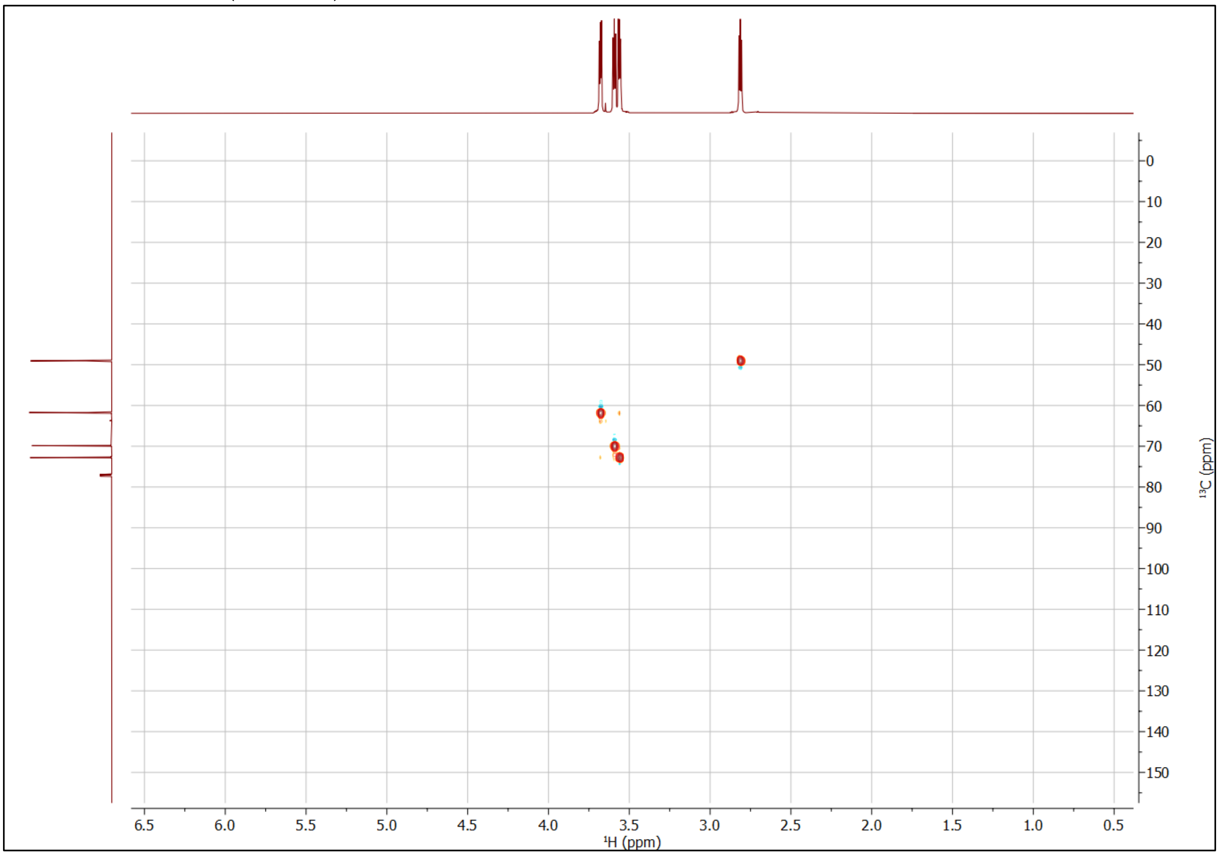

^{1}H-^{13}C-HMBC NMR ($CDCl_3$, **4**)

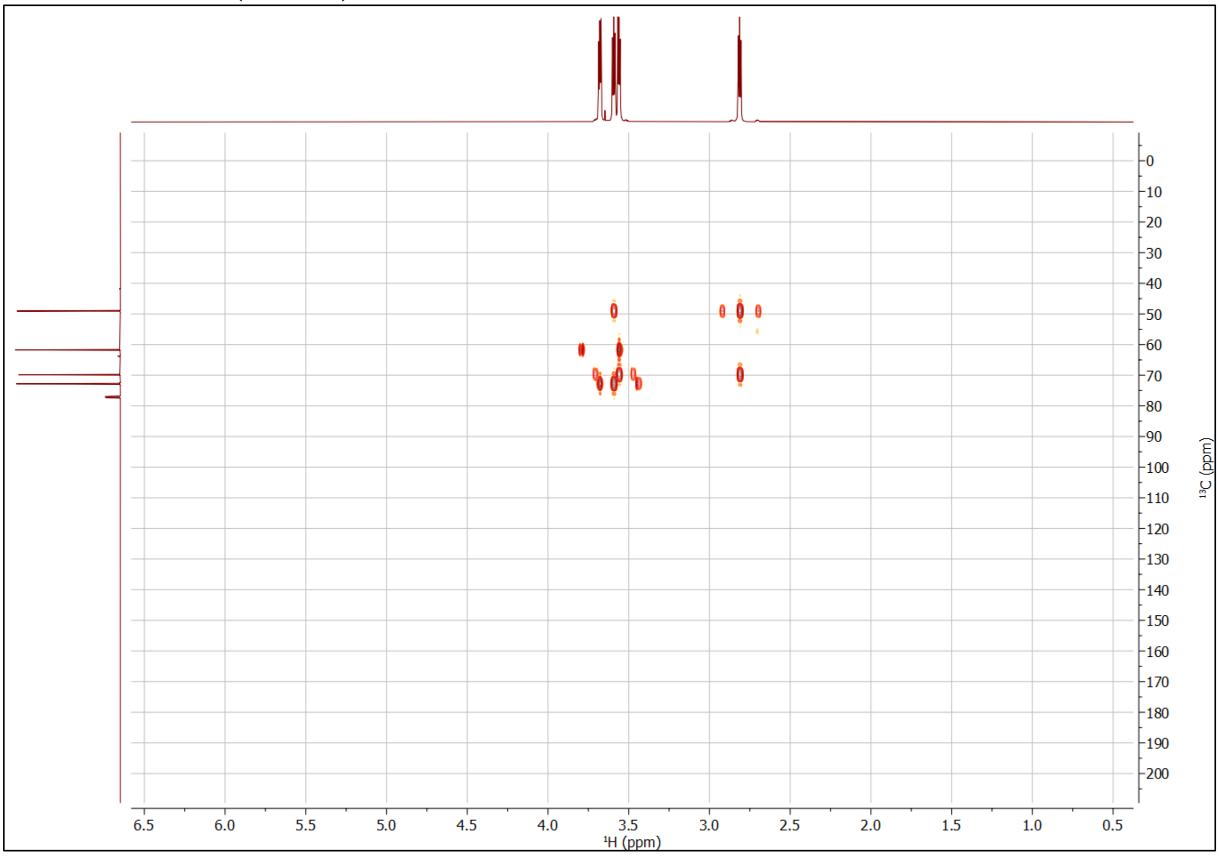

2,5-Dioxopyrrolidin-1-yl 5-((3a*S*,4*S*,6a*R*)-2-oxohexahydro-1*H*-thieno[3,4-*d*]imidazol-4-yl)pentanoate (**3**)

^{1}H NMR (600 MHz, DMSO-d_6)

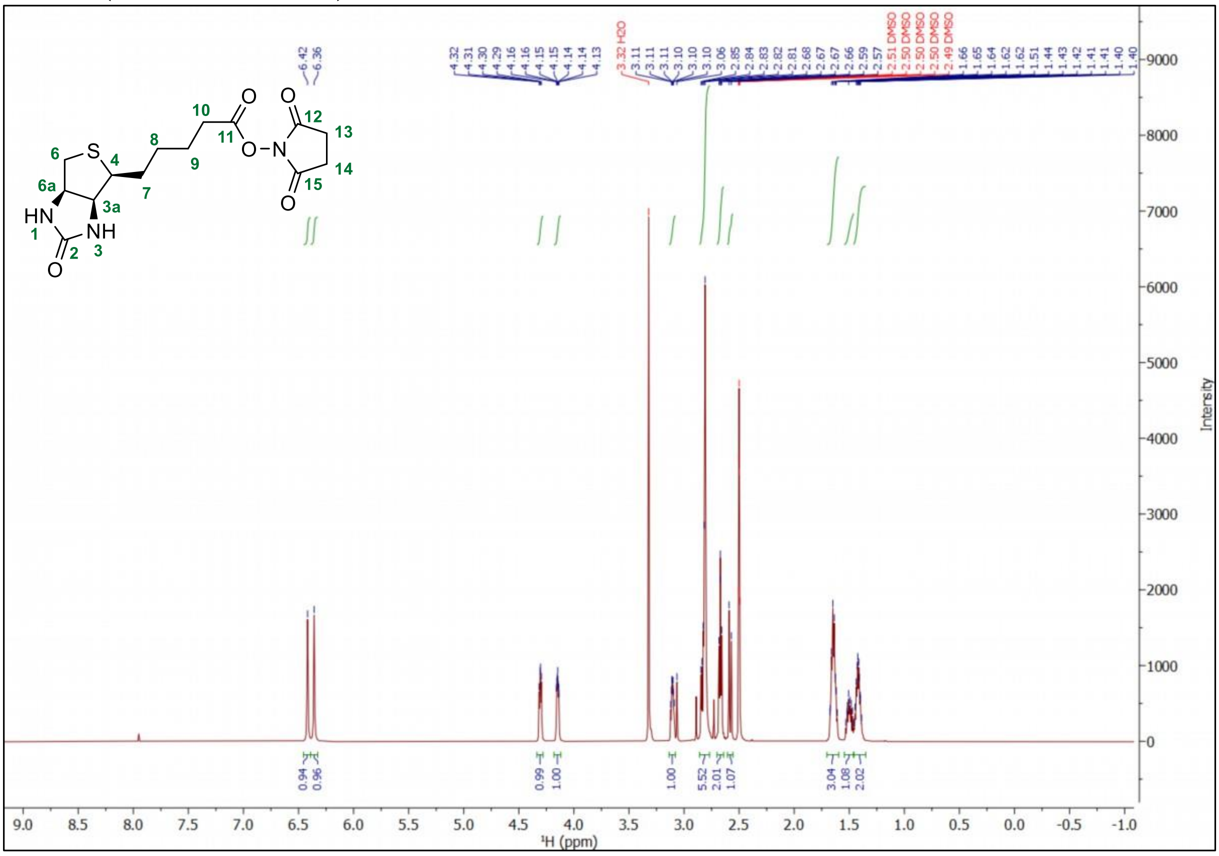

^{13}C NMR (151 MHz, DMSO-d_6)

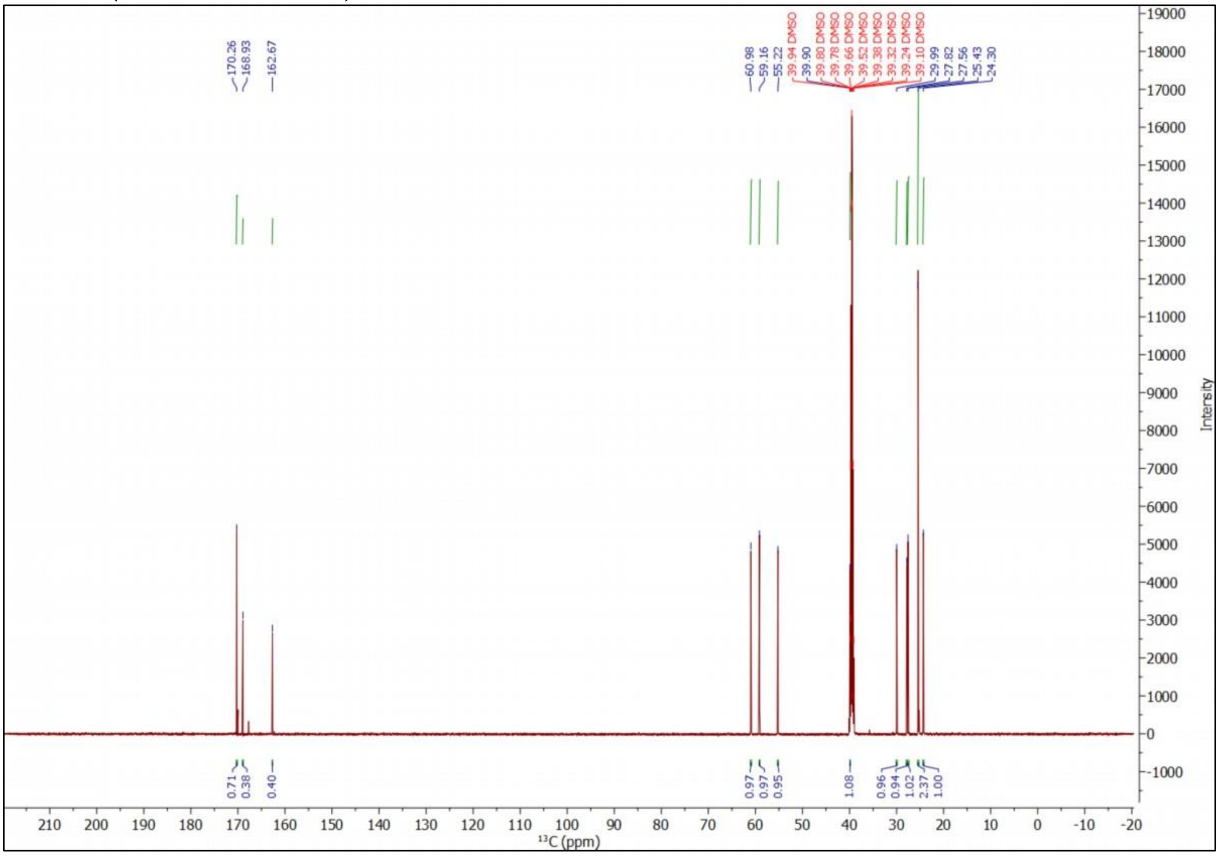

DEPT135 NMR (DMSO-d_6, **3**)

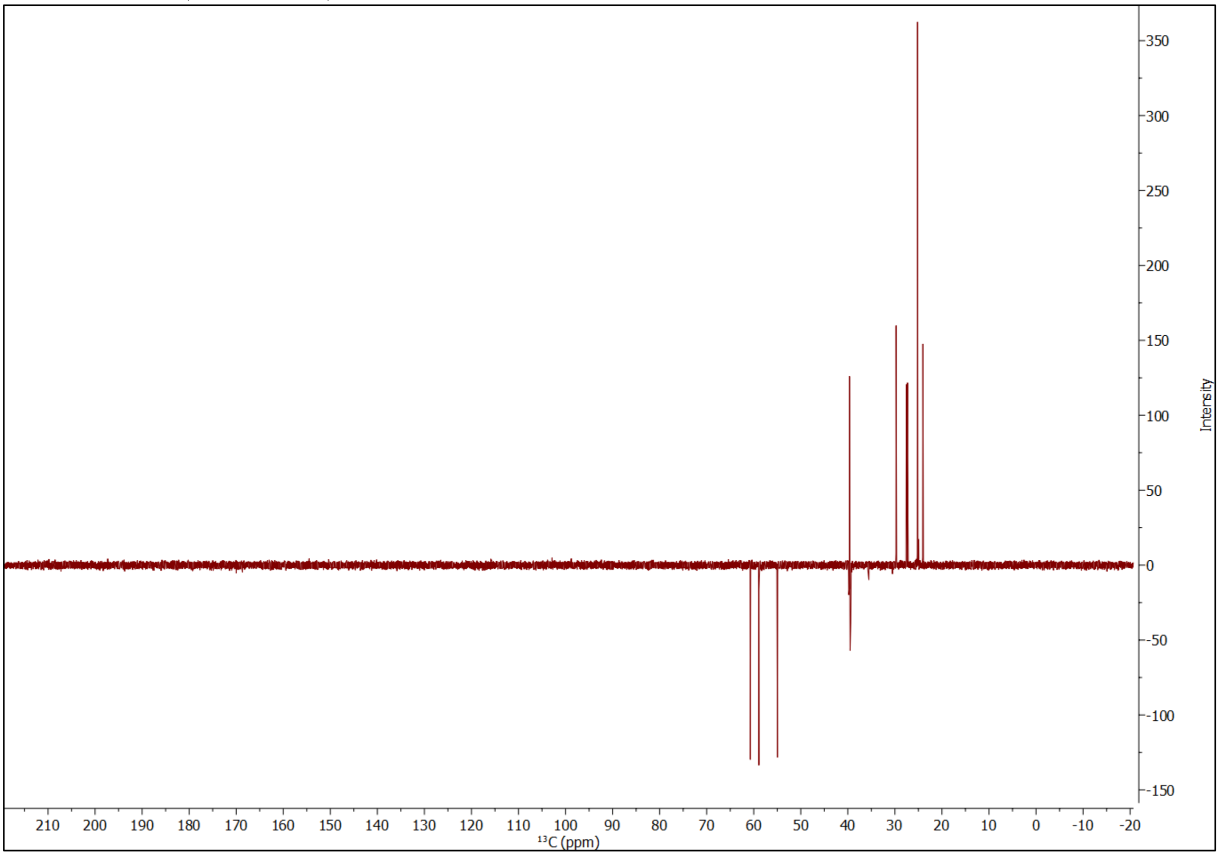

^{1}H-^{1}H-COSY NMR (DMSO-d_6, **3**)

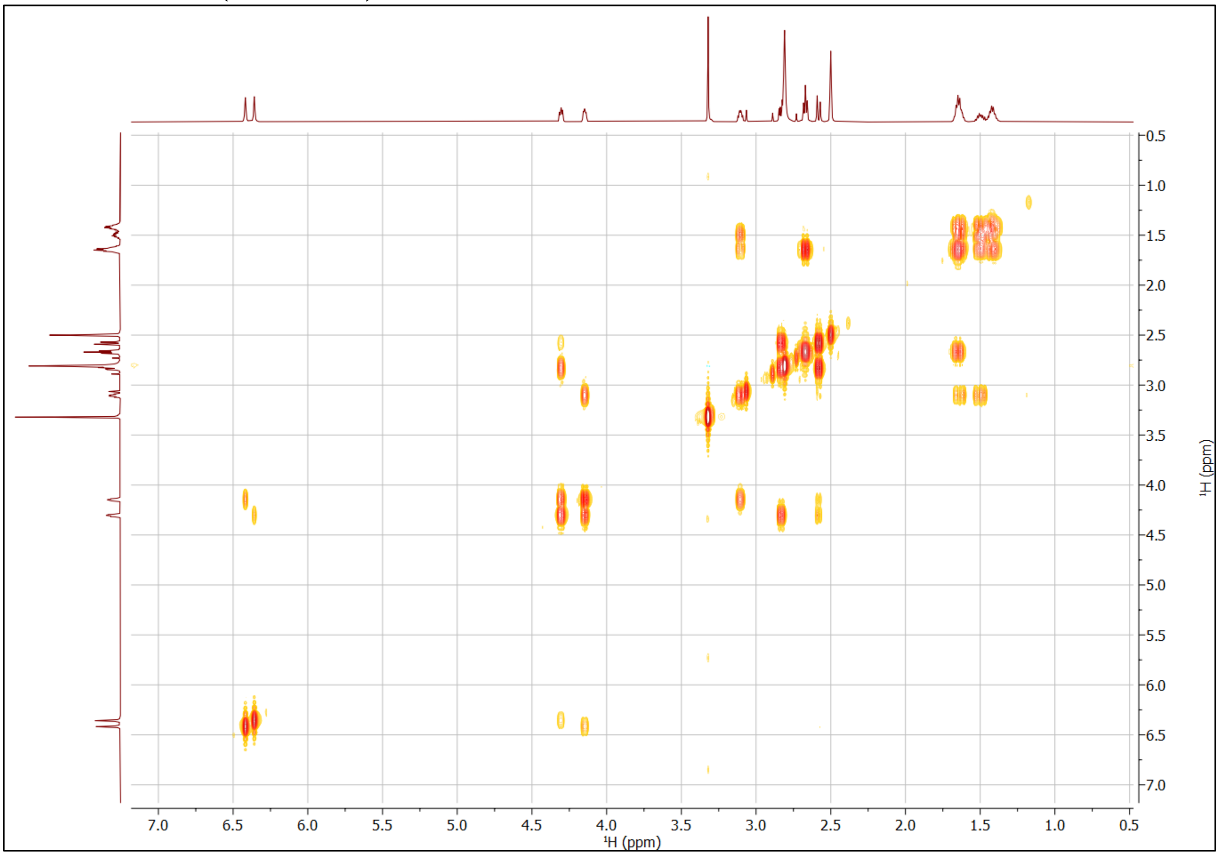

^{1}H-^{13}C-HSQC NMR (DMSO-d_6, **3**)

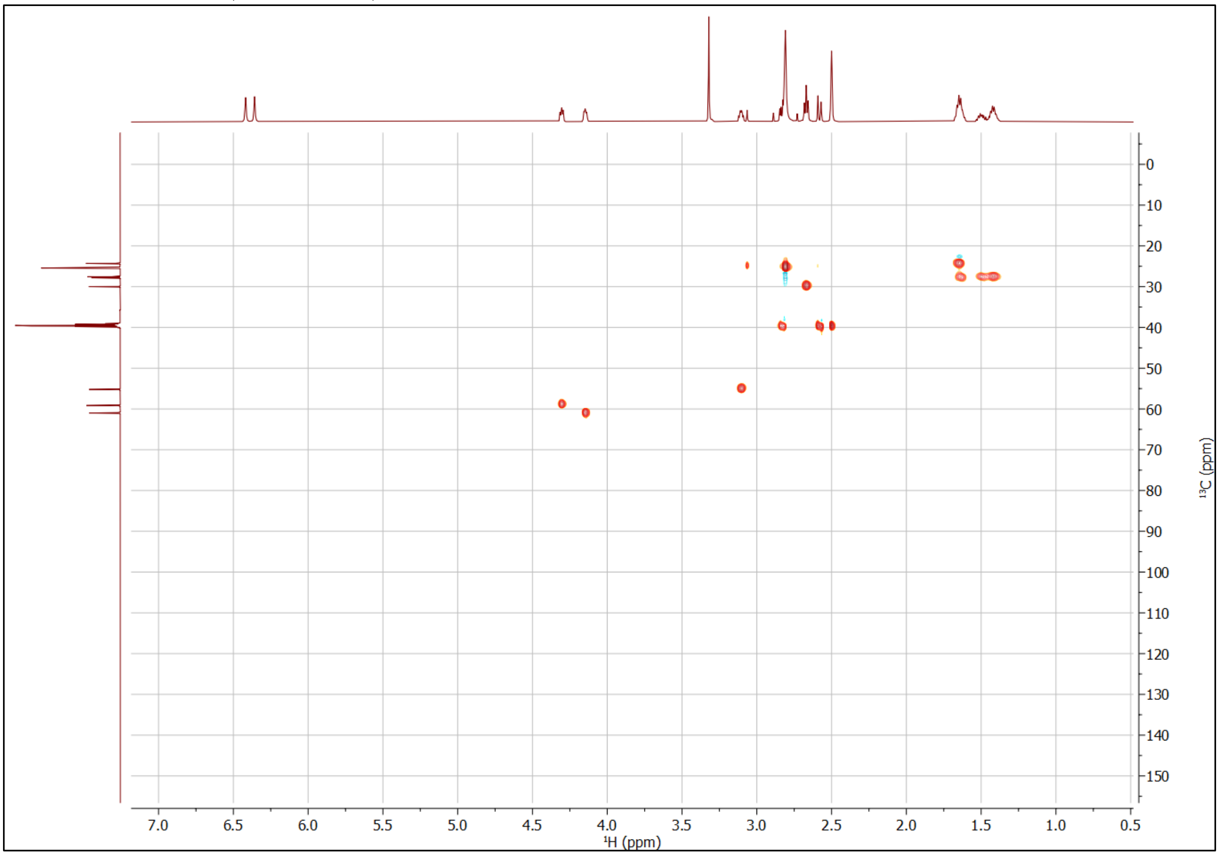

^{1}H-^{13}C-HMBC NMR (DMSO-d_6, **3**)

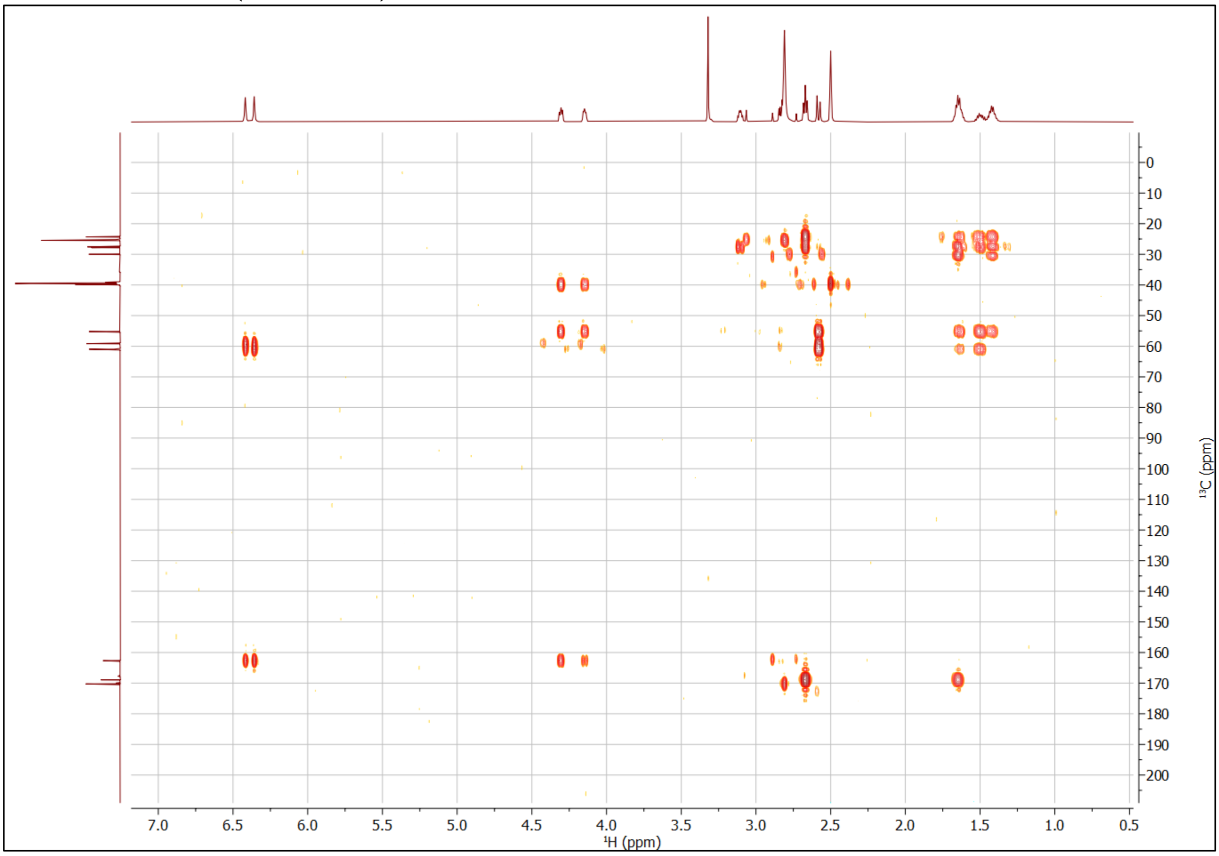

N,*N*-Bis(2-(2-hydroxyethoxy)ethyl)-5-((3a*S*,4*S*,6a*R*)-2-oxohexahydro-1*H*-thieno[3,4-*d*]imidazol-4-yl)pentanamide (**7**)

^{1}H NMR (600 MHz, MeOD)

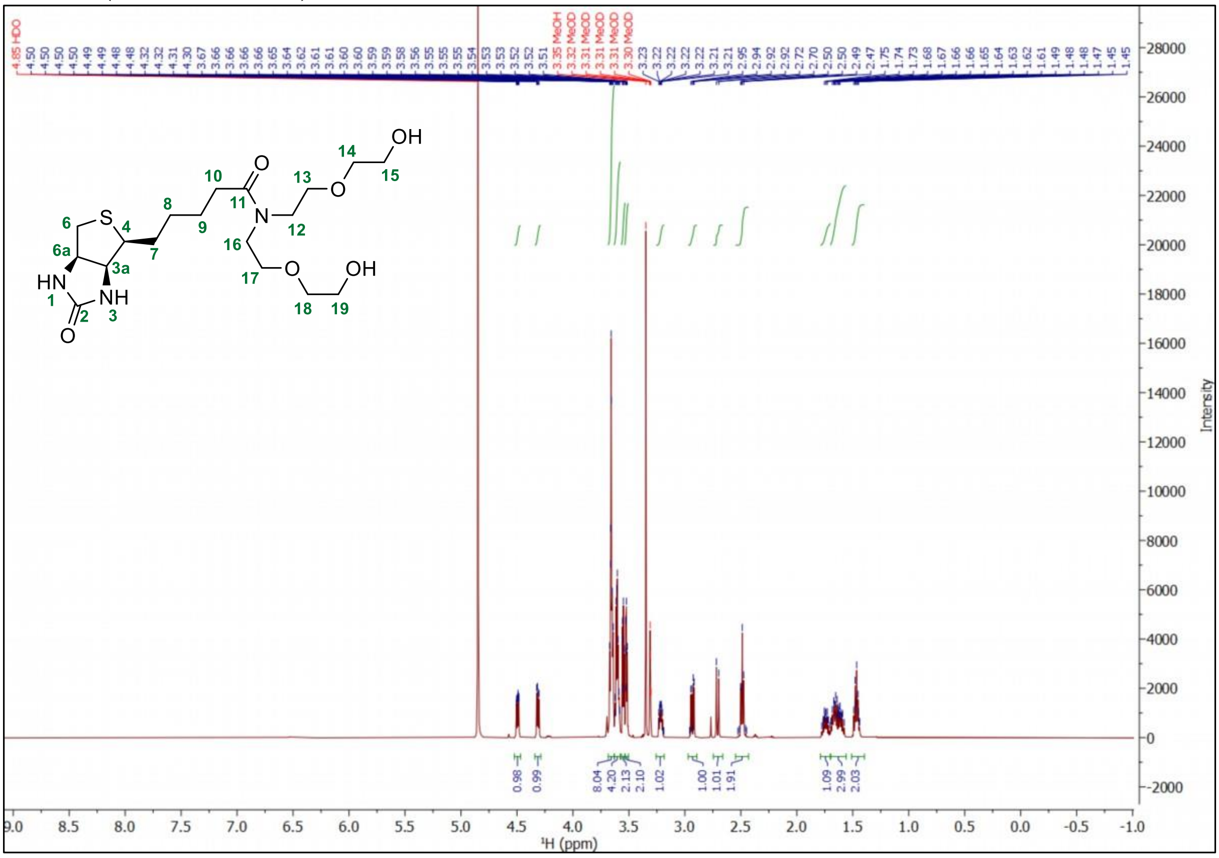

^{13}C NMR (151 MHz, MeOD)

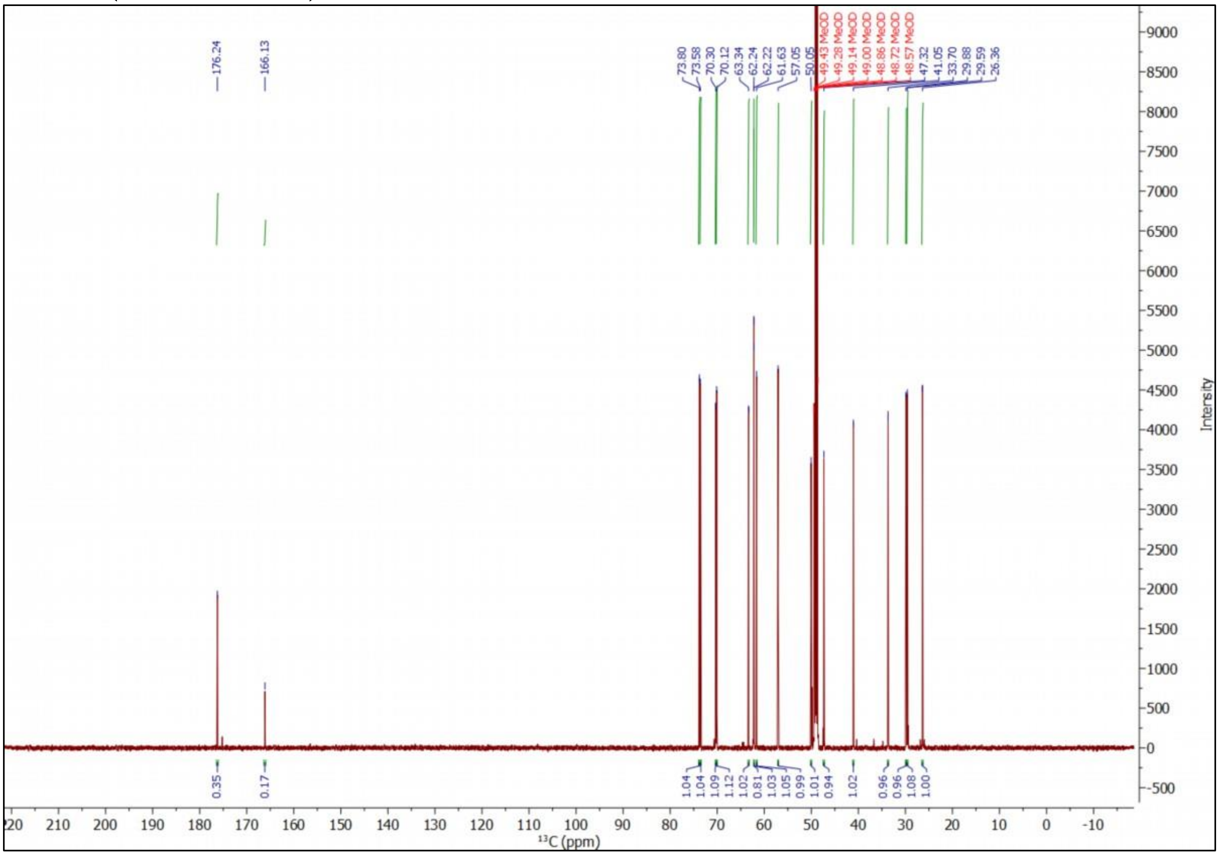

DEPT135 NMR (MeOD, **7**)

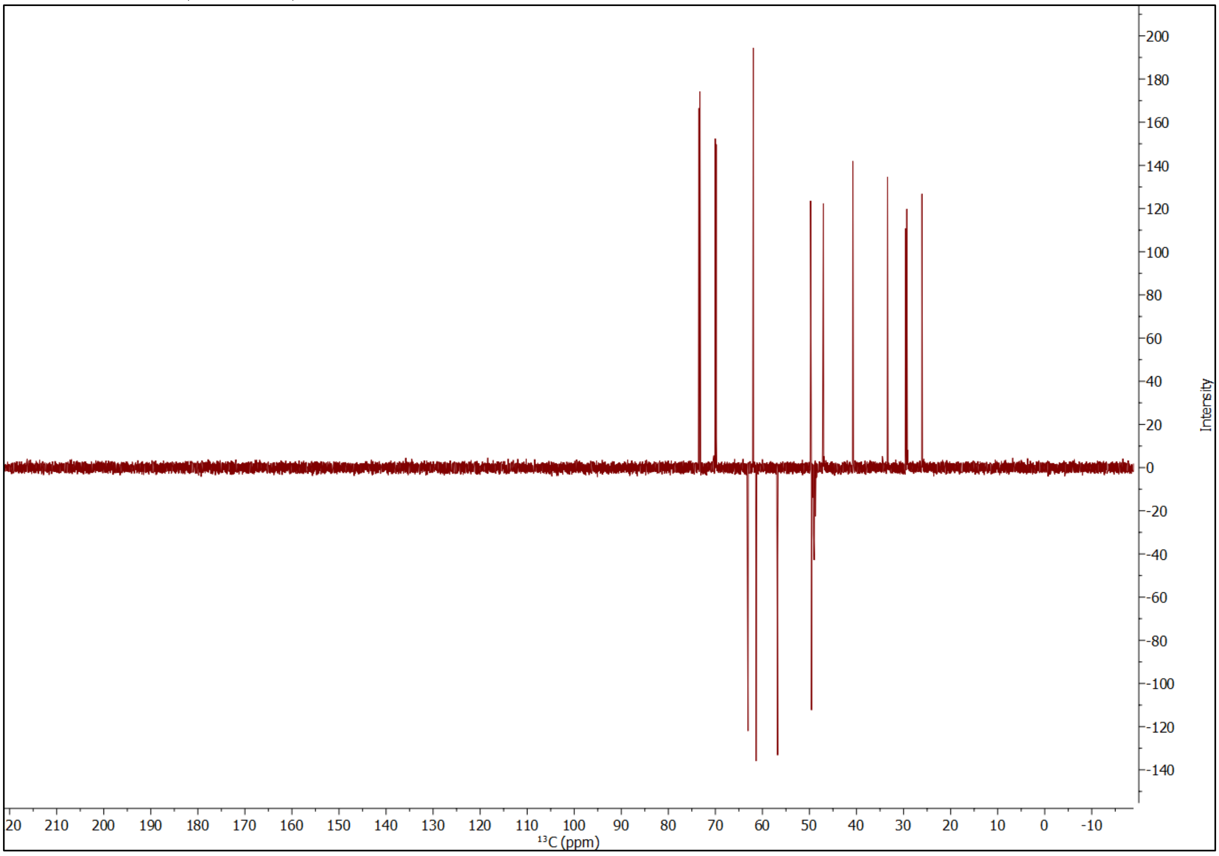

^{1}H-^{1}H-COSY NMR (MeOD, **7**)

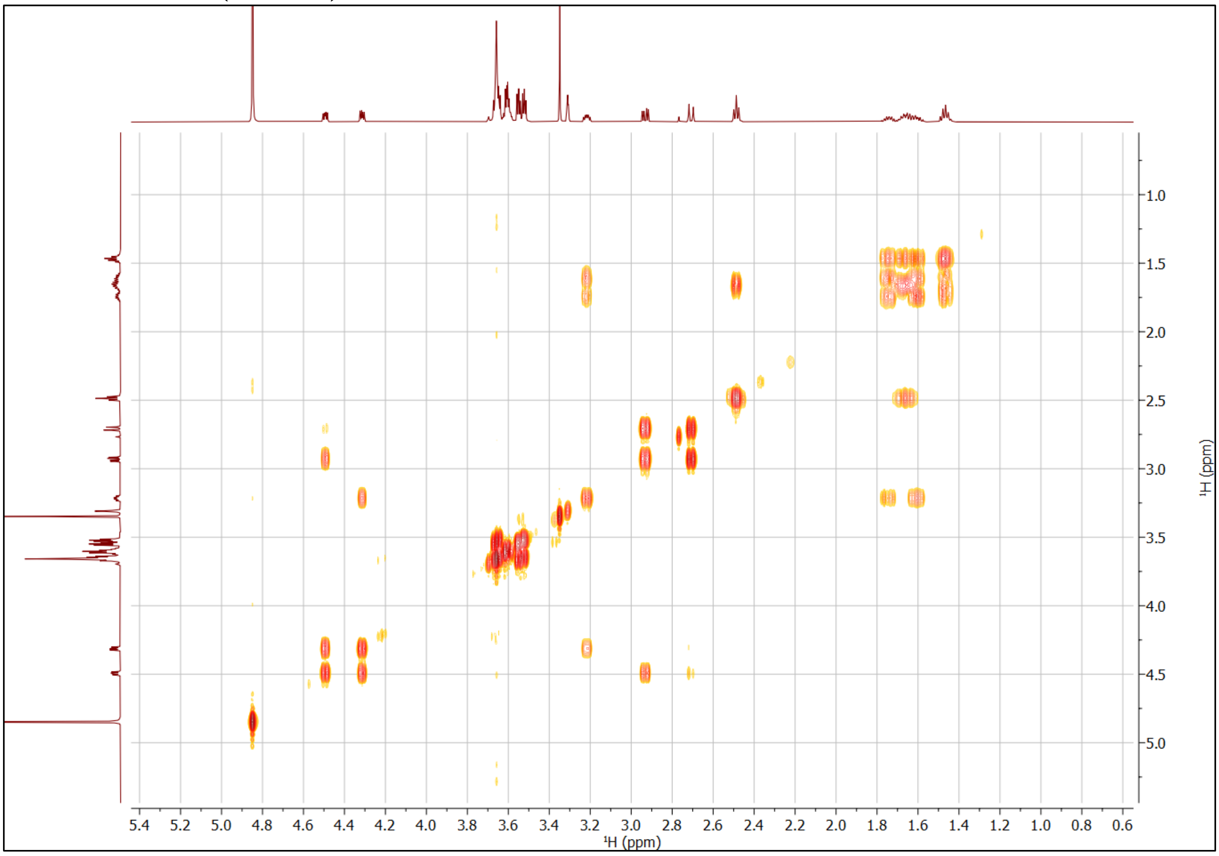

^{1}H-^{13}C-HSQC NMR (MeOD, **7**)

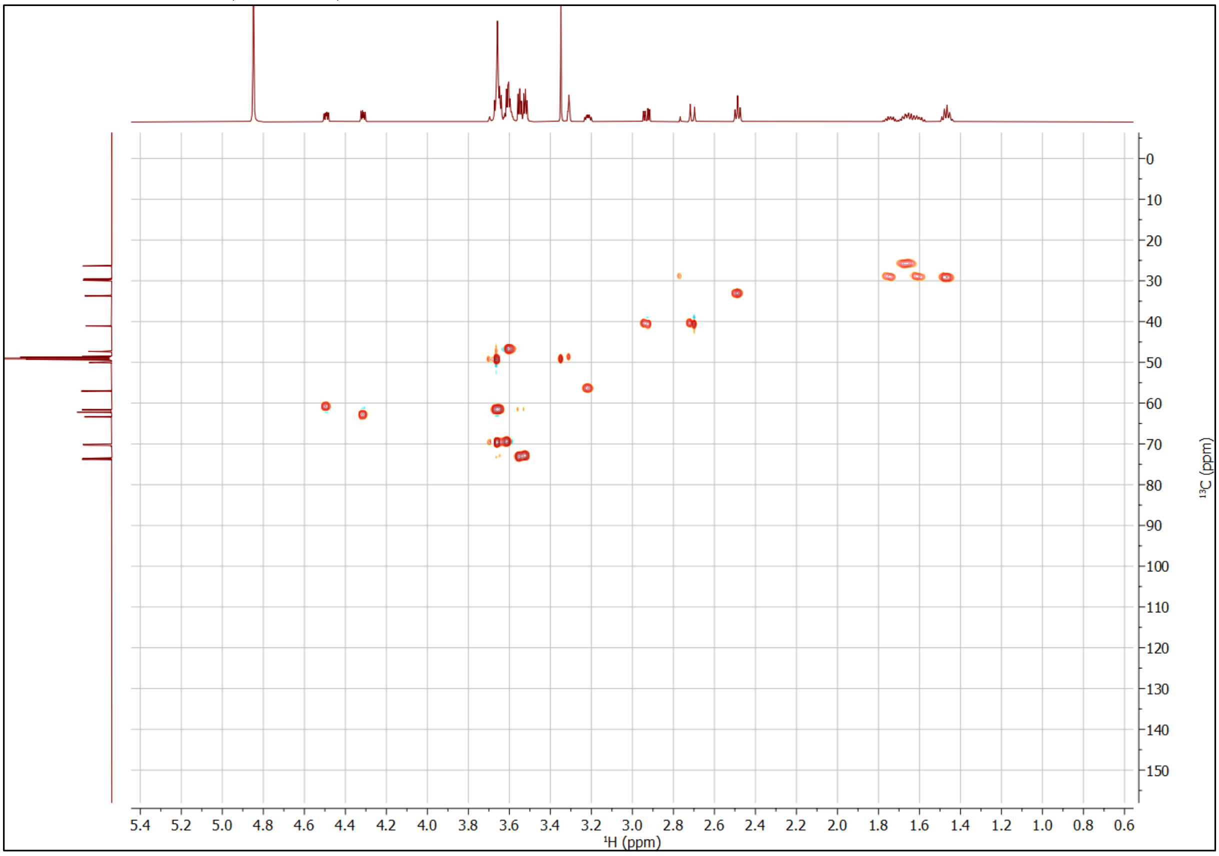

^{1}H-^{13}C-HMBC NMR (MeOD, **7**)

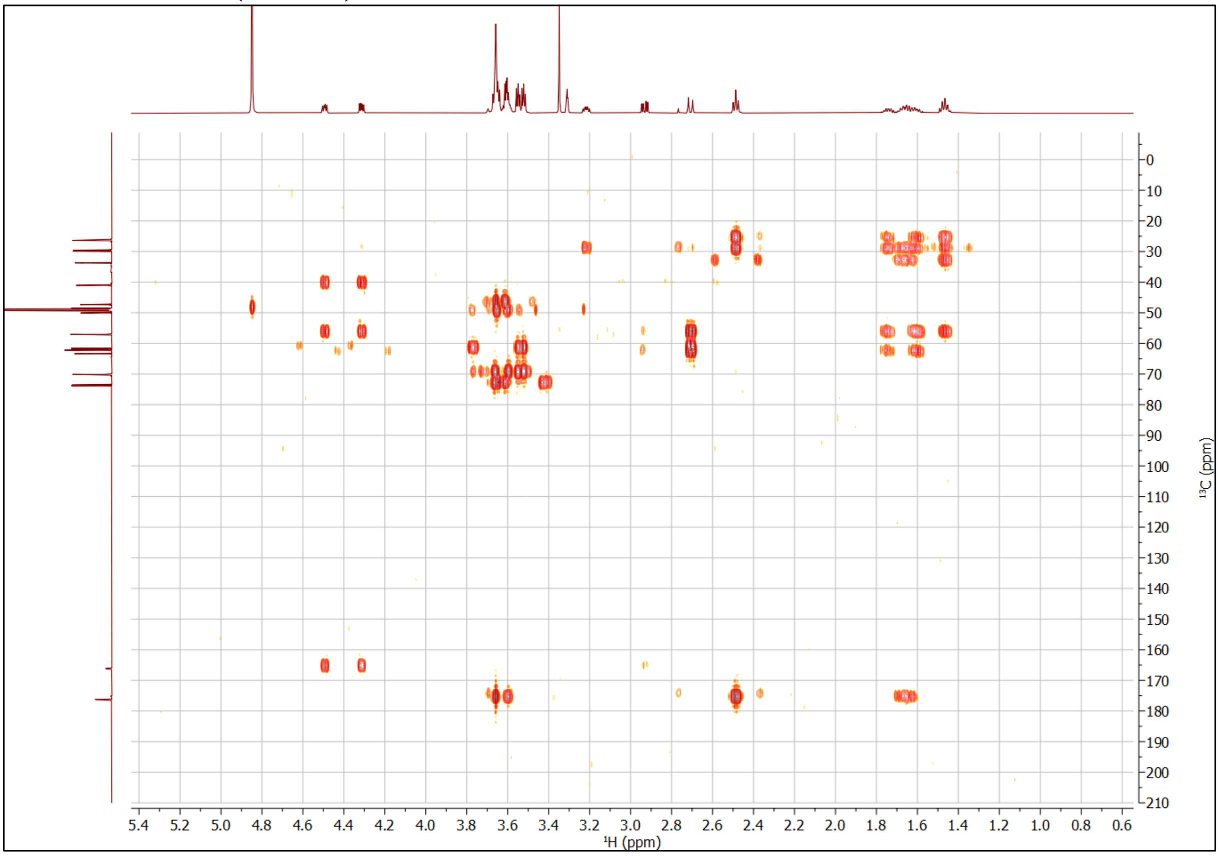

^{1}H-^{1}H-ROESY NMR (MeOD, **7**)

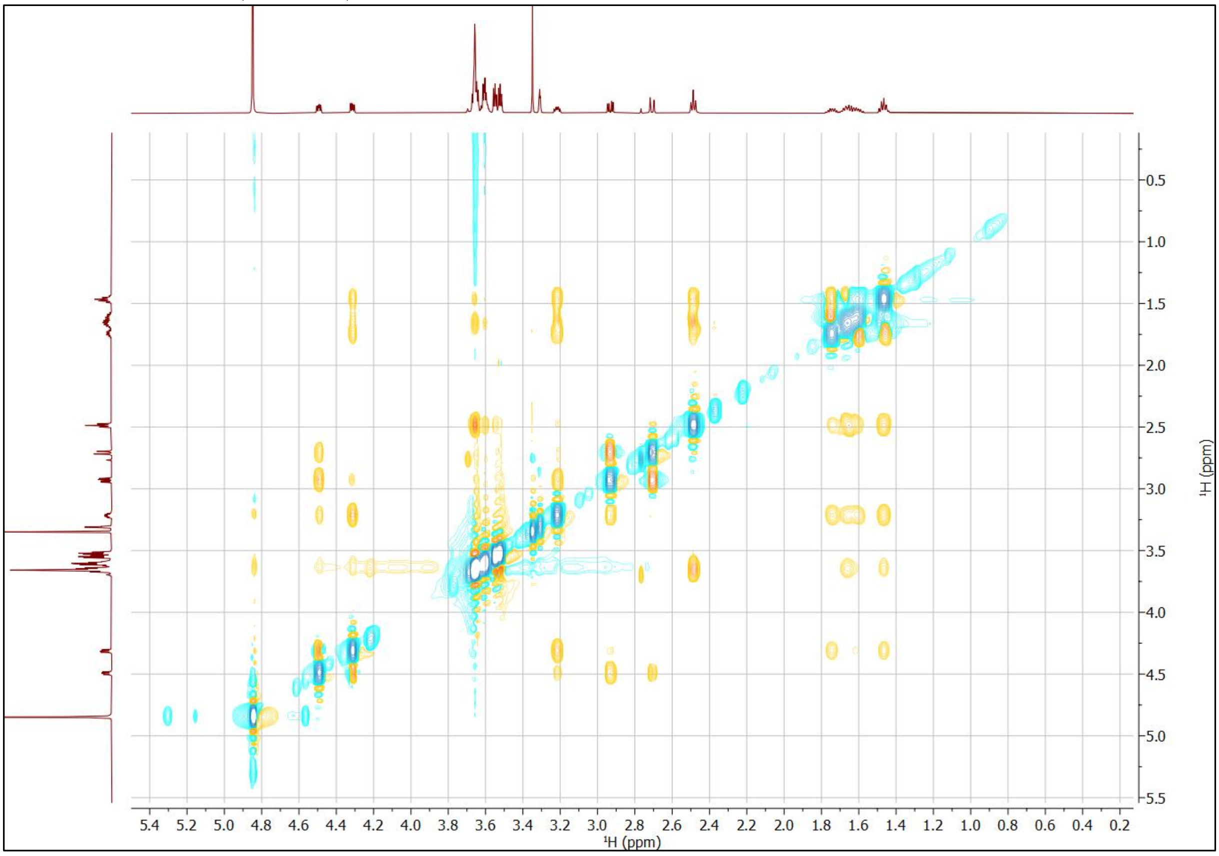

N,*N*-Bis(2-(2-iodoethoxy)ethyl)-5-((3a*S*,4*S*,6a*R*)-2-oxohexahydro-1*H*-thieno[3,4-*d*]imidazol-4-yl)pentanamide (**8**)

^{1}H NMR (600 MHz, $CDCl_3$)

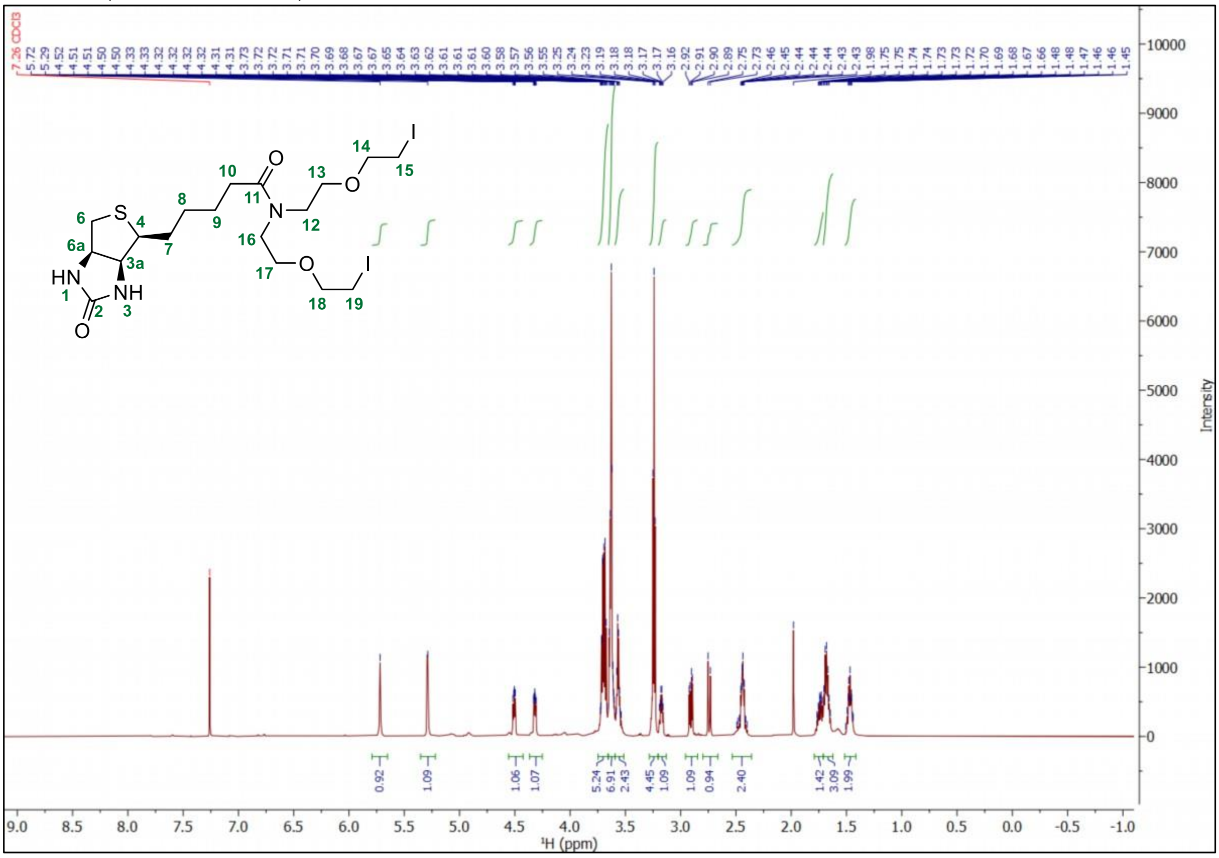

^{13}C NMR (151 MHz, $CDCl_3$)

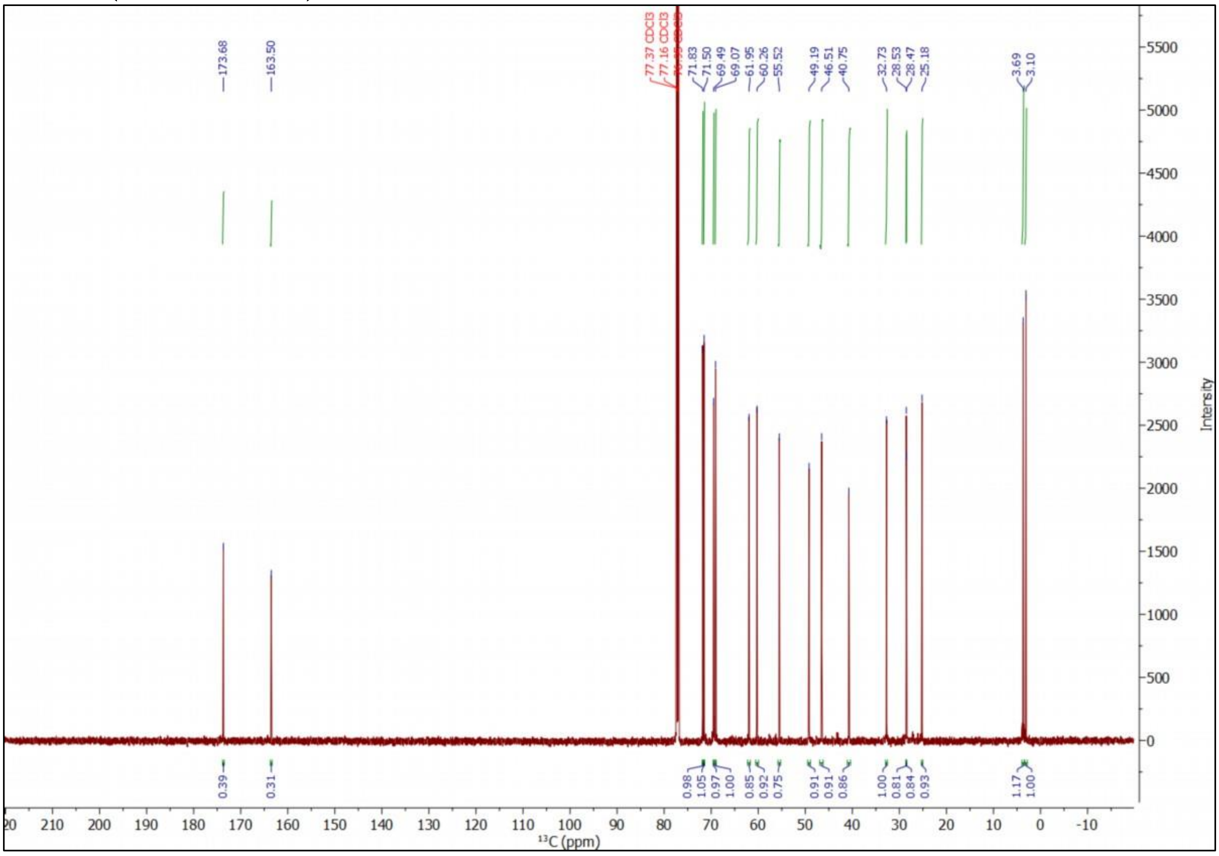

DEPT135 NMR (CDCl$_3$, **8**)

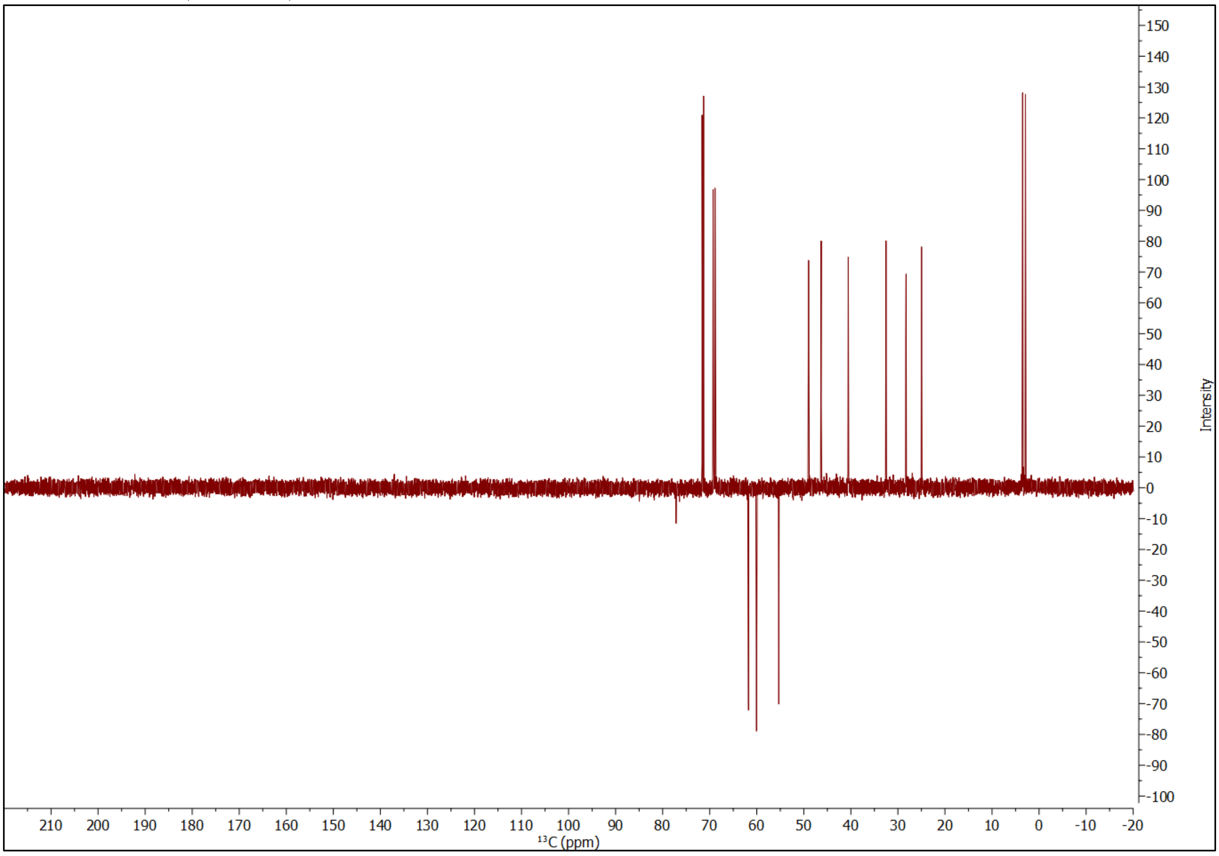

^{1}H-^{1}H-COSY NMR (CDCl$_3$, **8**)

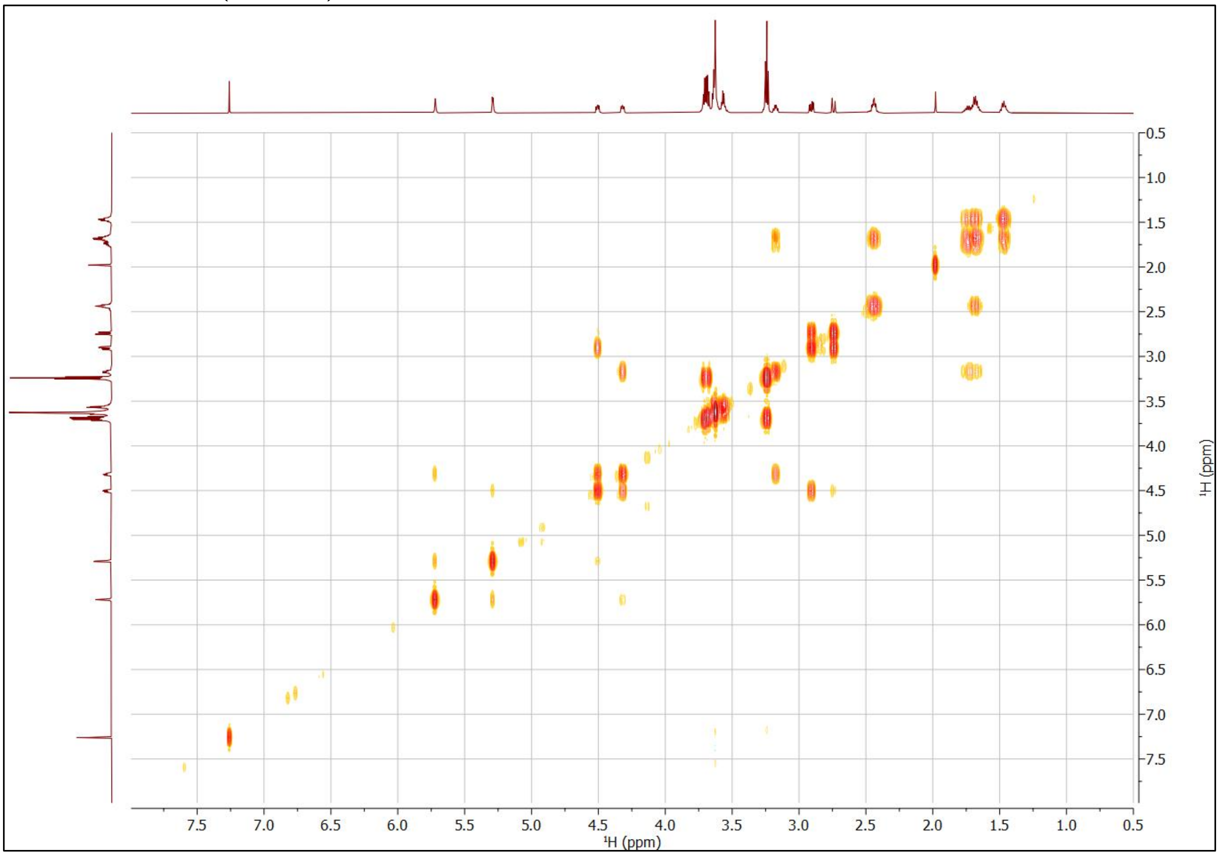

^{1}H-^{13}C-HSQC NMR ($CDCl_3$, **8**)

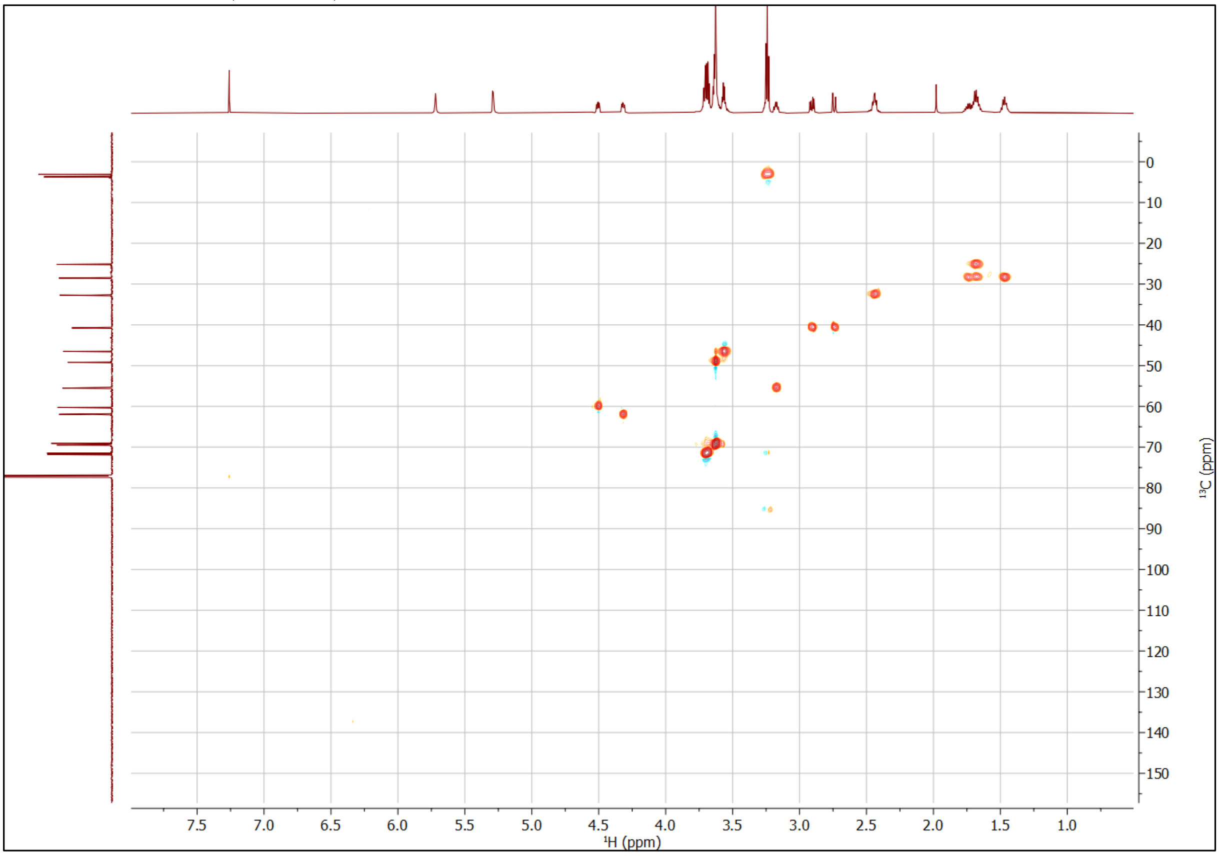

^{1}H-^{13}C-HMBC NMR ($CDCl_3$, **8**)

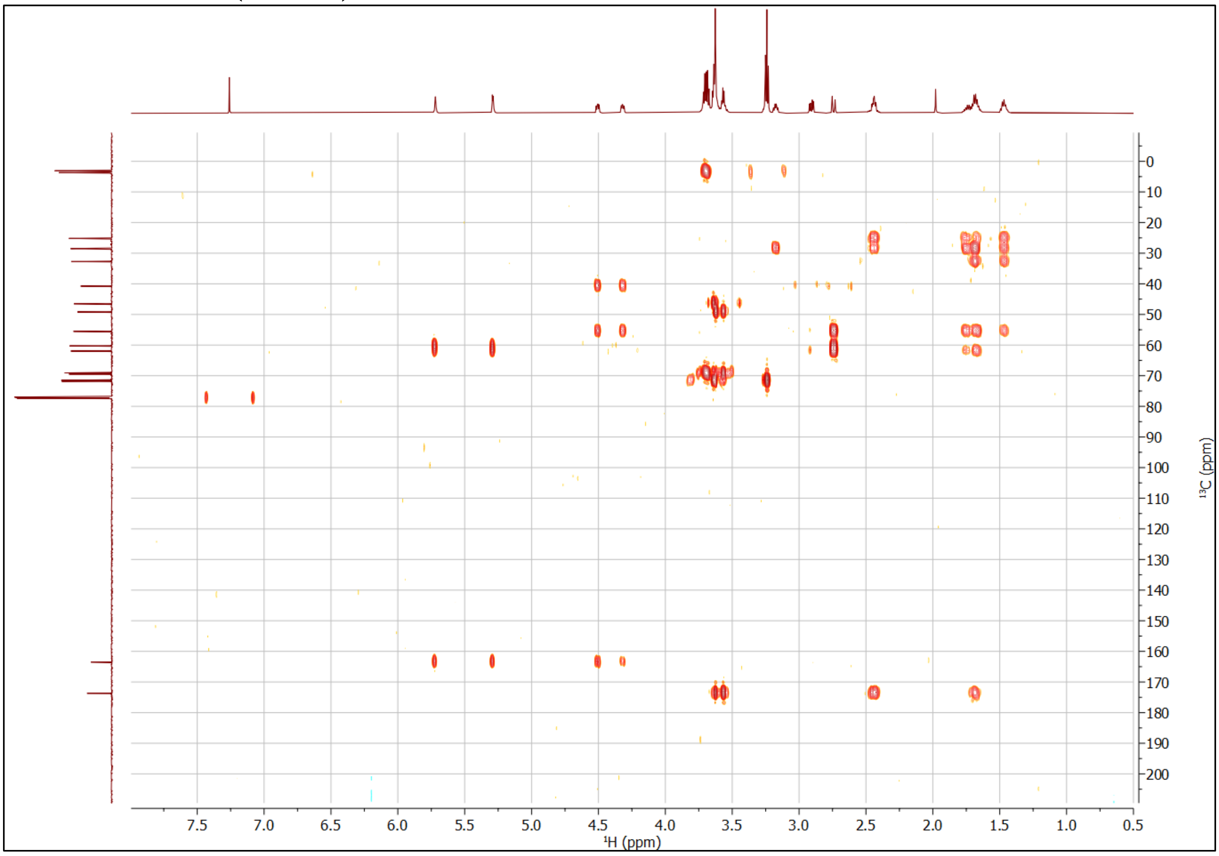

^1H-^1H-ROESY NMR ($CDCl_3$, **8**)

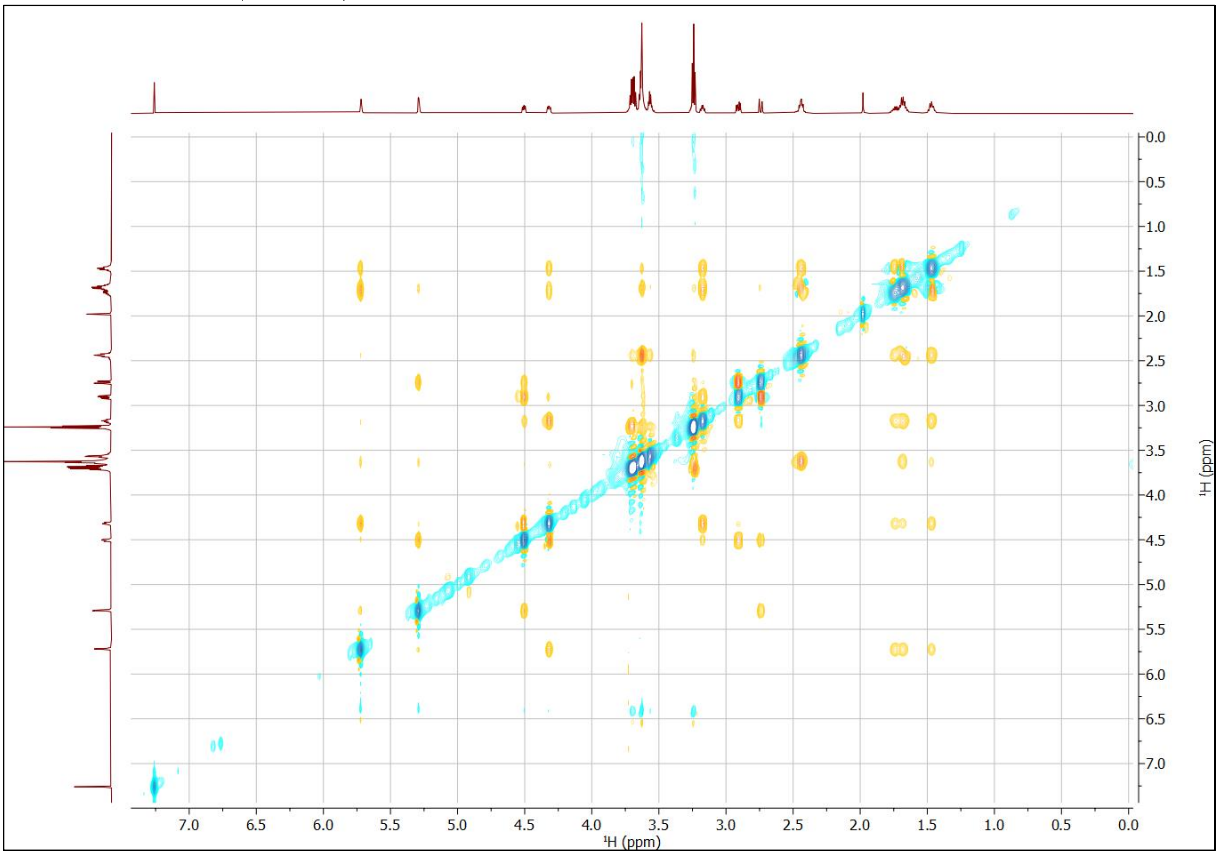

N,*N*-Bis(2-(2-azidoethoxy)ethyl)-5-((3a*S*,4*S*,6a*R*)-2-oxohexahydro-1*H*-thieno[3,4-*d*]imidazol-4-yl)pentanamide (**9**)

^{1}H NMR (600 MHz, $CDCl_3$)

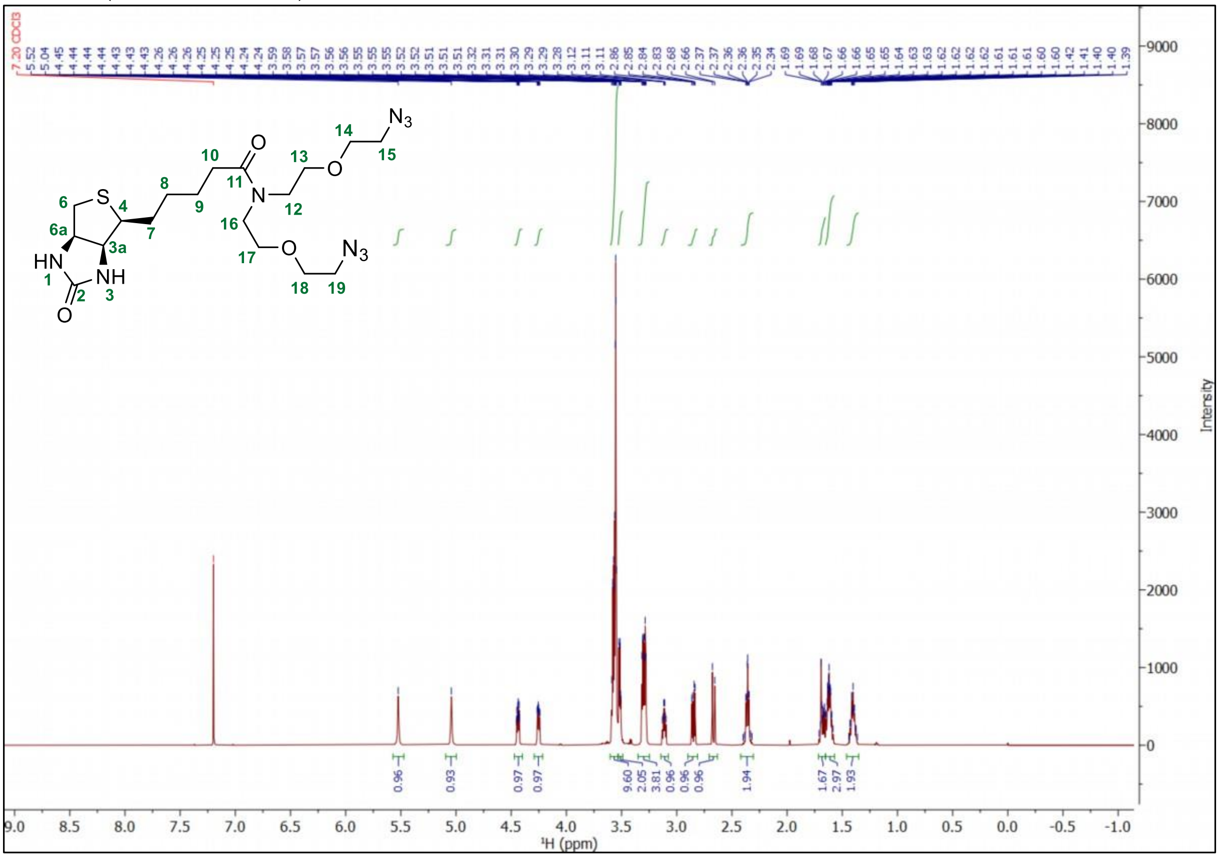

^{13}C NMR (151 MHz, $CDCl_3$)

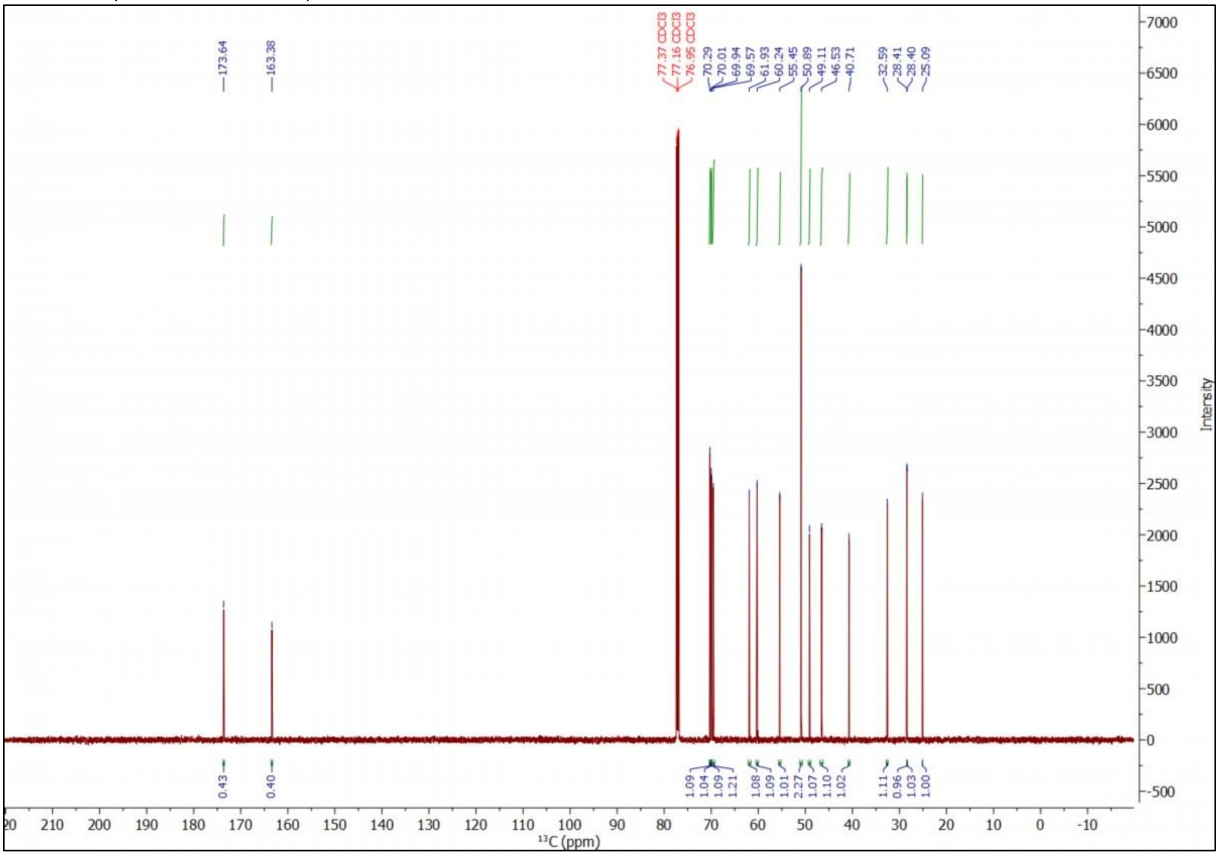

DEPT135 NMR ($CDCl_3$, **9**)

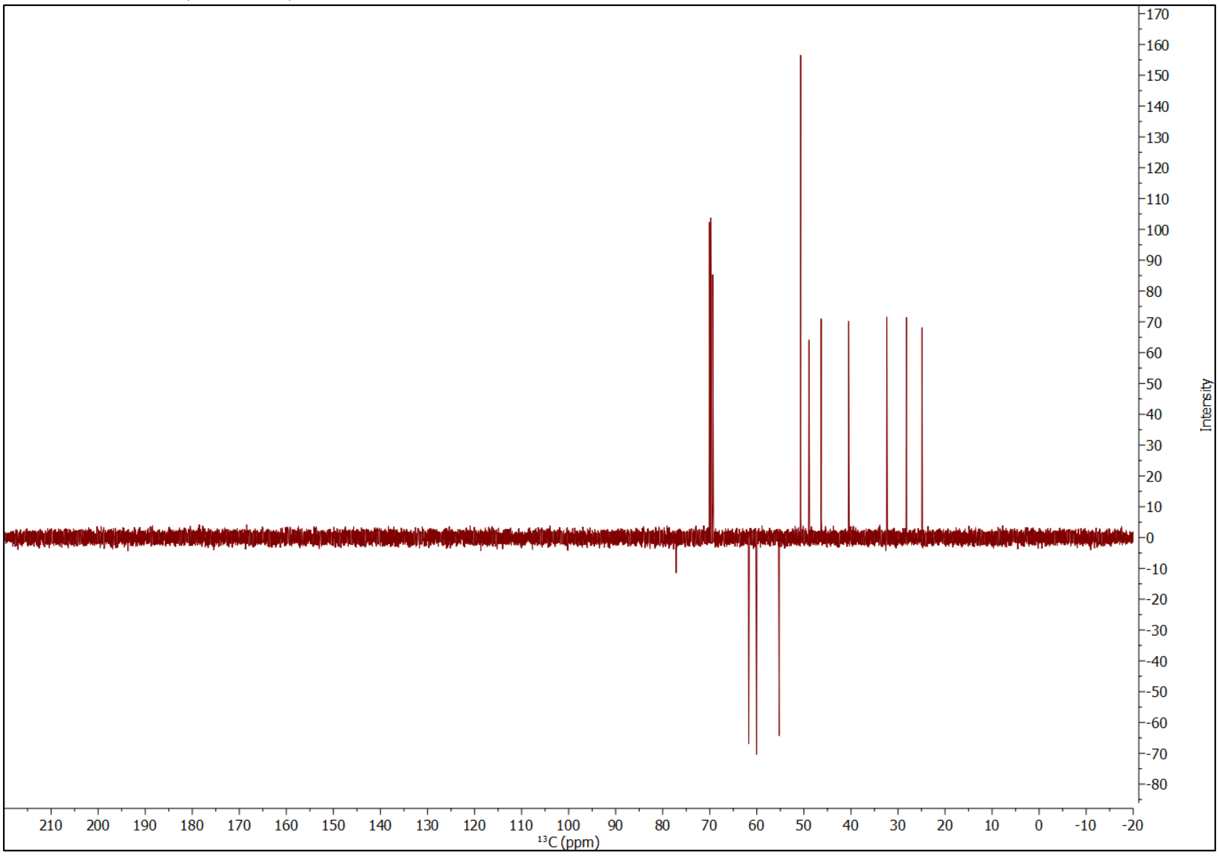

^1H-^1H-COSY NMR ($CDCl_3$, **9**)

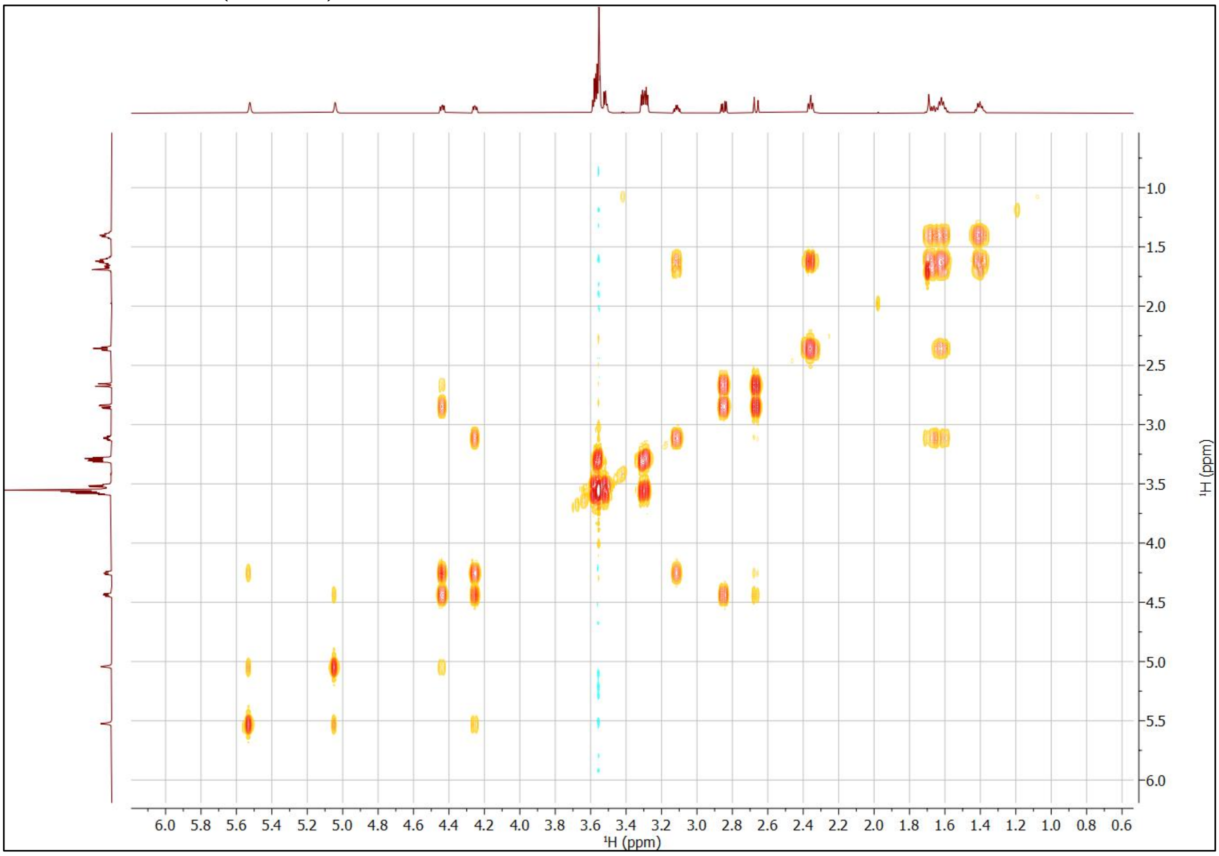

^{1}H-^{13}C-HSQC NMR ($CDCl_3$, **9**)

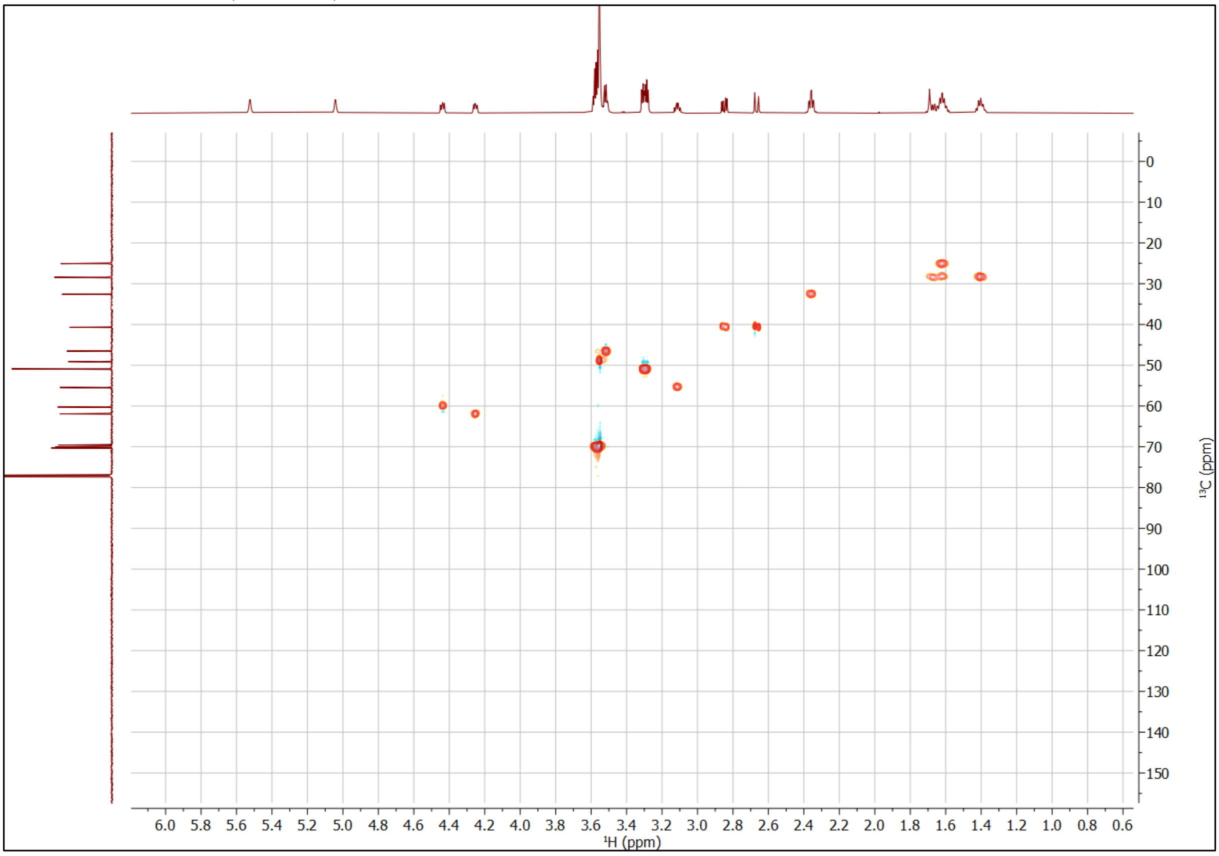

^{1}H-^{13}C-HMBC NMR ($CDCl_3$, **9**)

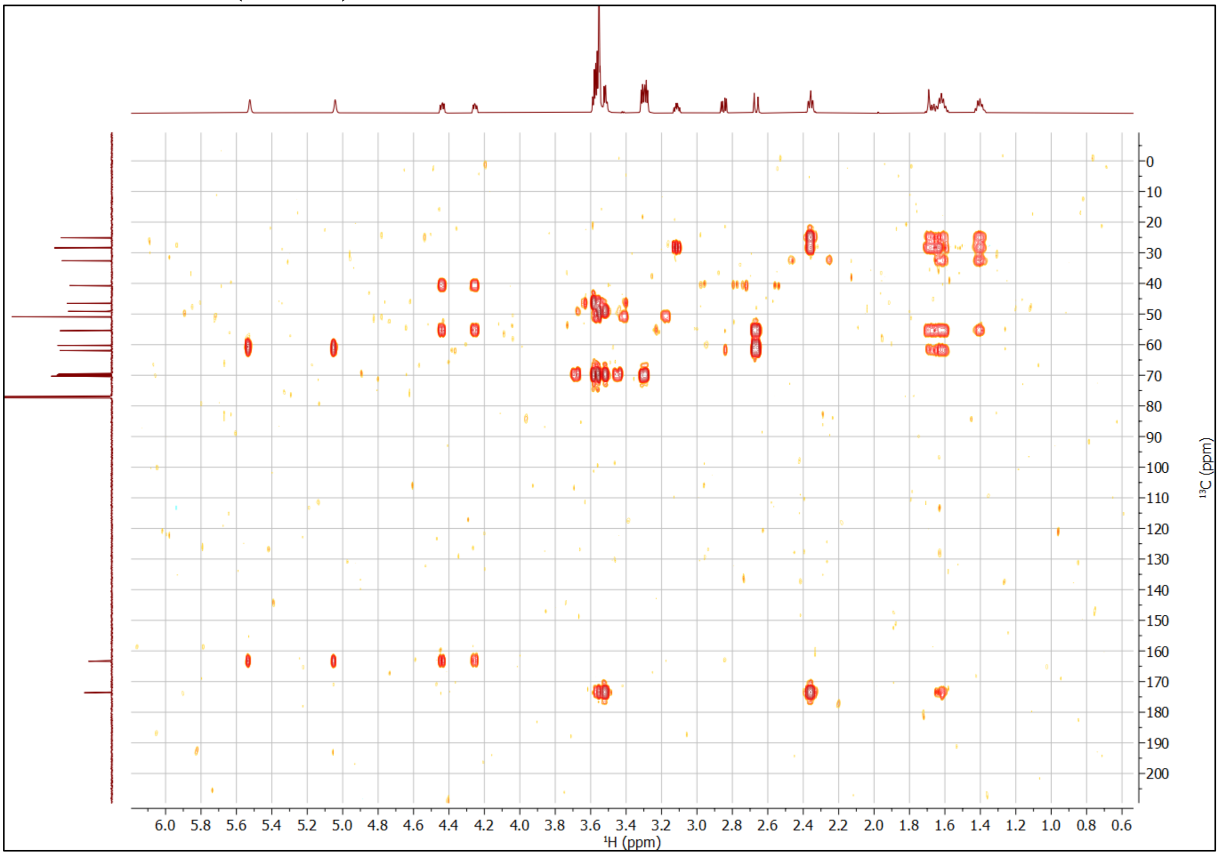

2-Amino-5-methoxybenzenethiol (**12**)

^{1}H NMR (600 MHz, $CDCl_3$)

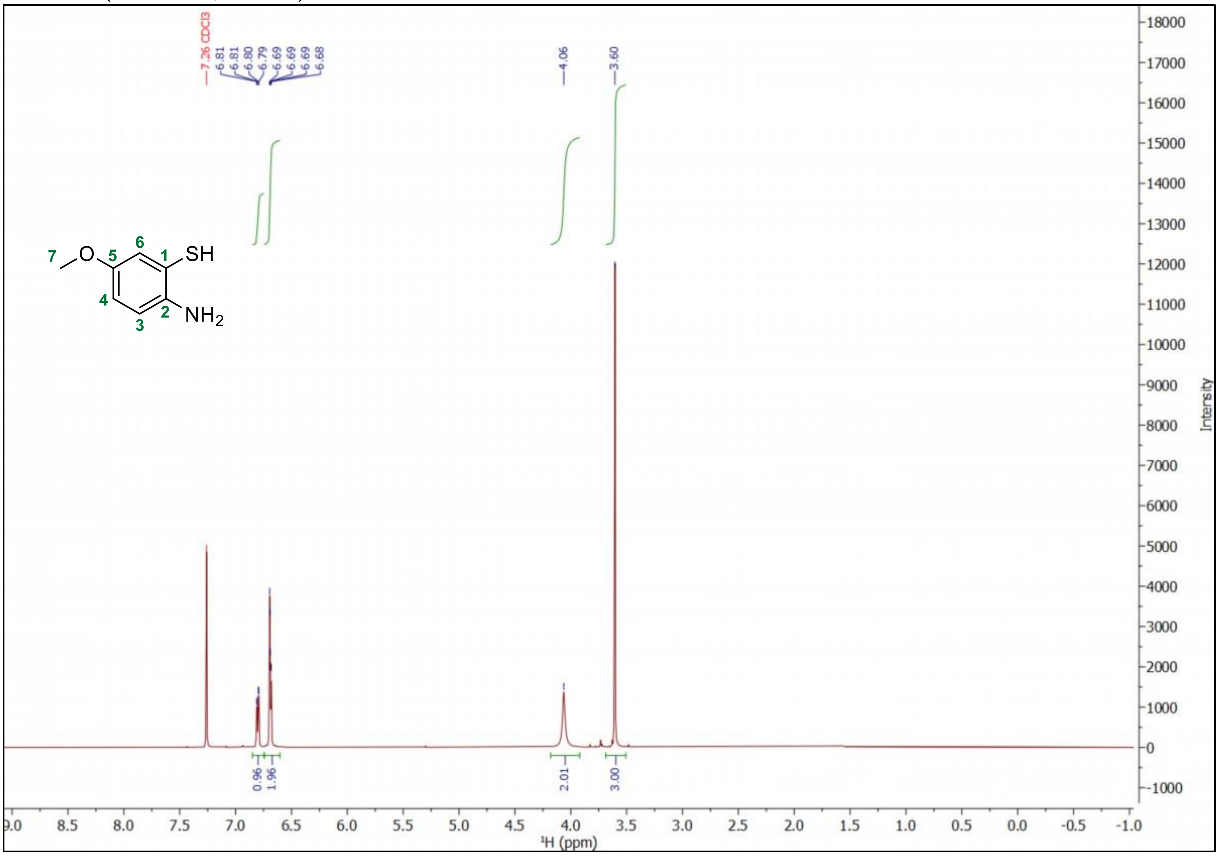

^{13}C NMR (151 MHz, $CDCl_3$)

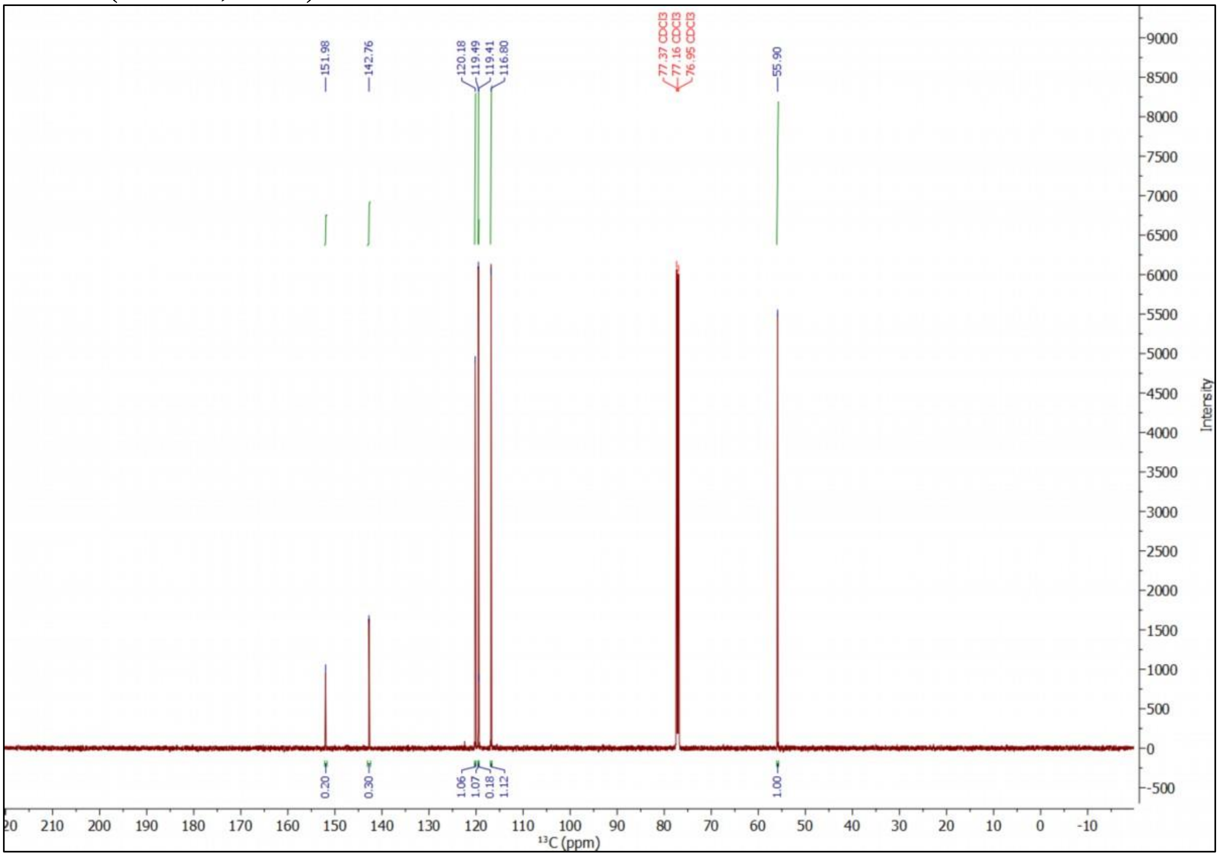

DEPT135 NMR ($CDCl_3$, **12**)

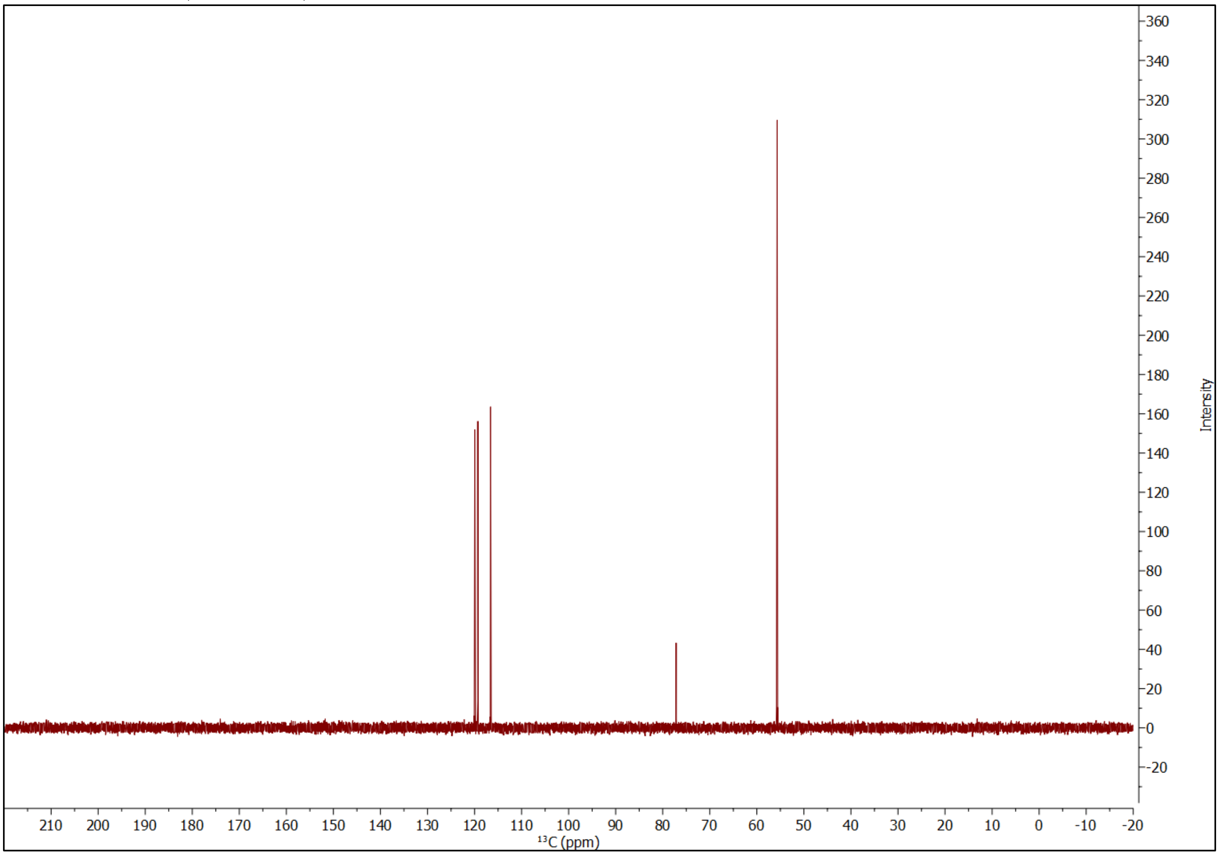

^{1}H-^{1}H-COSY NMR ($CDCl_3$, **12**)

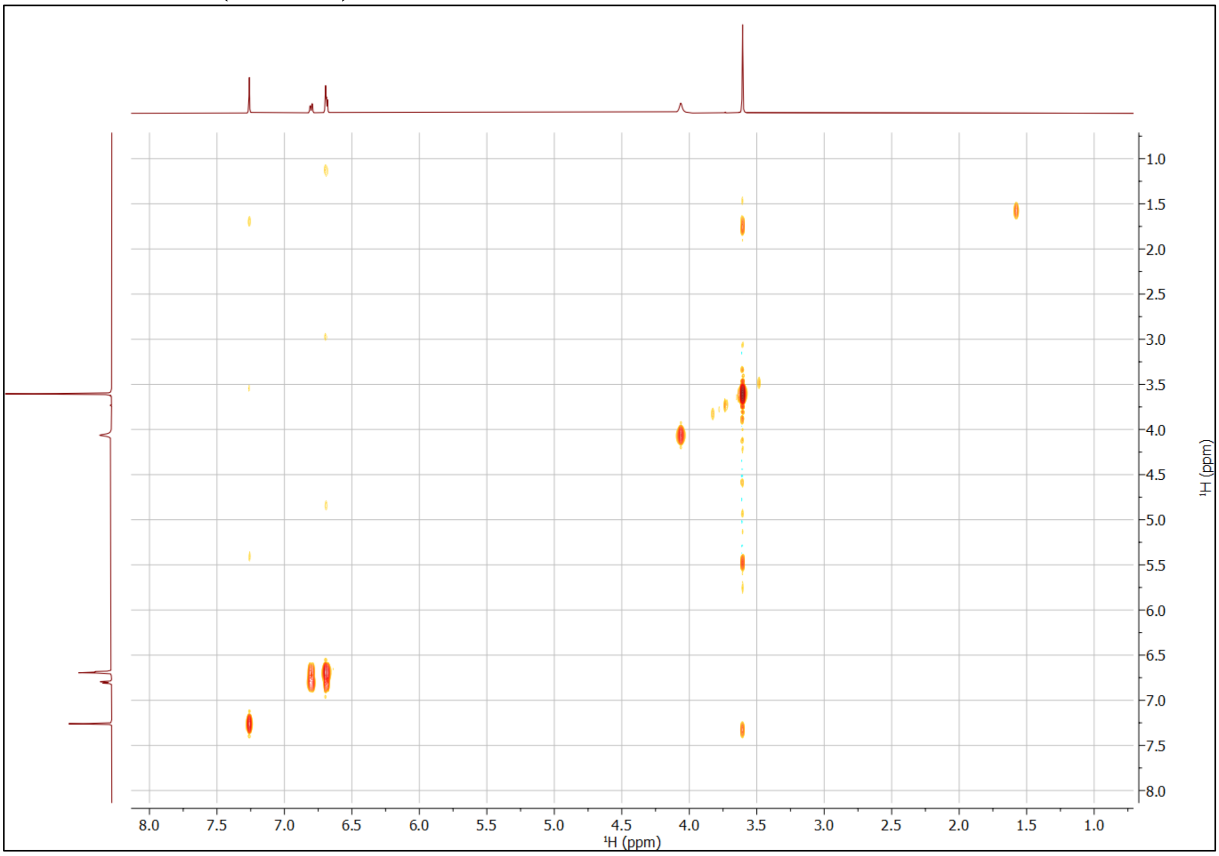

^{1}H-^{13}C-HSQC NMR ($CDCl_3$, **12**)

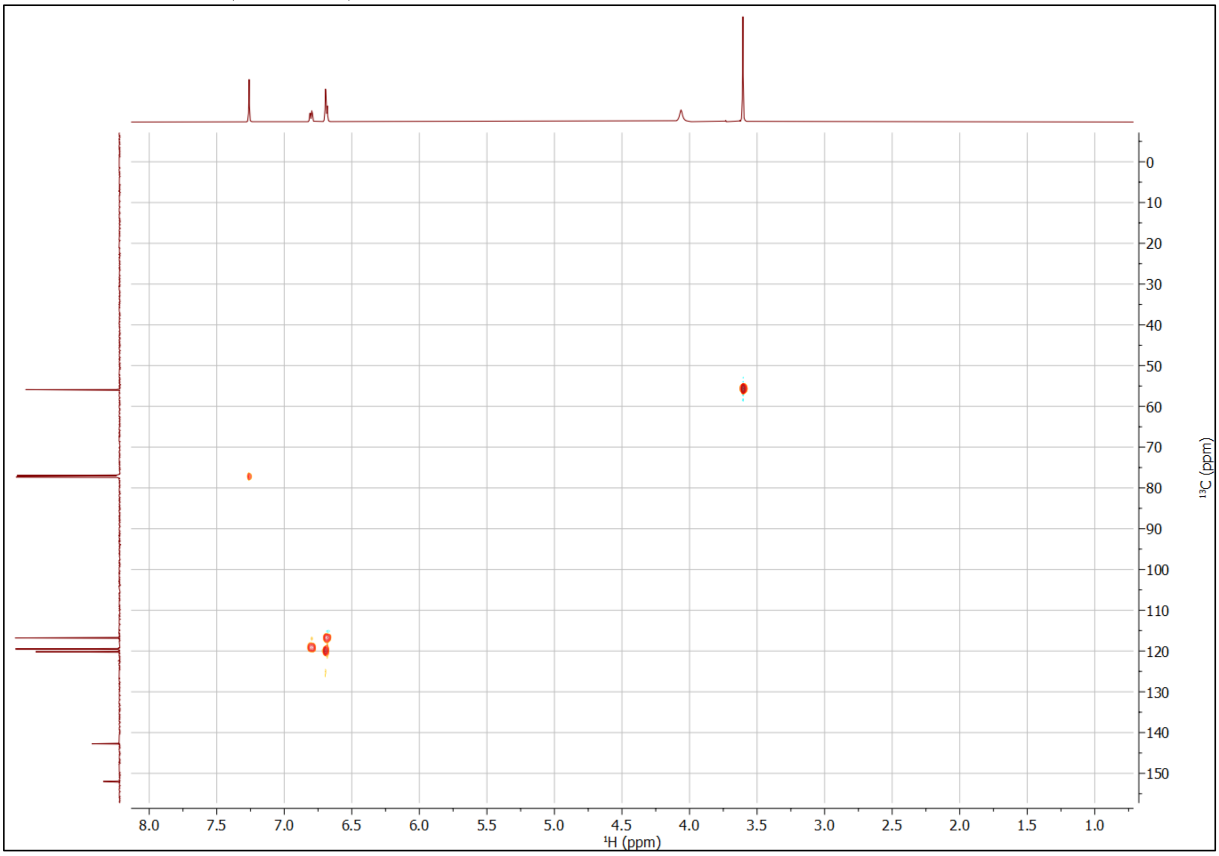

^{1}H-^{13}C-HMBC NMR ($CDCl_3$, **12**)

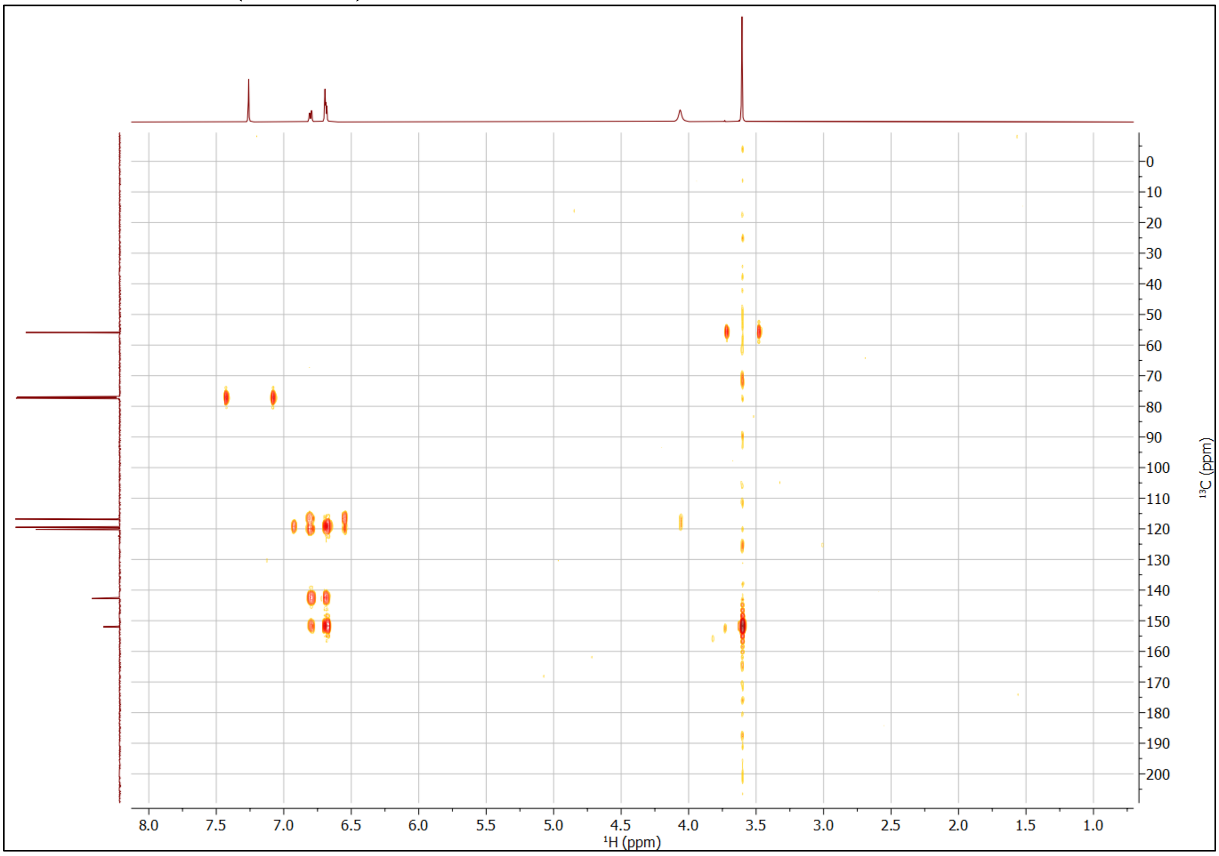

4-(6-methoxybenzo[*d*]thiazol-2-yl)-*N*,*N*-dimethylaniline (**14**)

^{1}H NMR (600 MHz, $CDCl_3$)

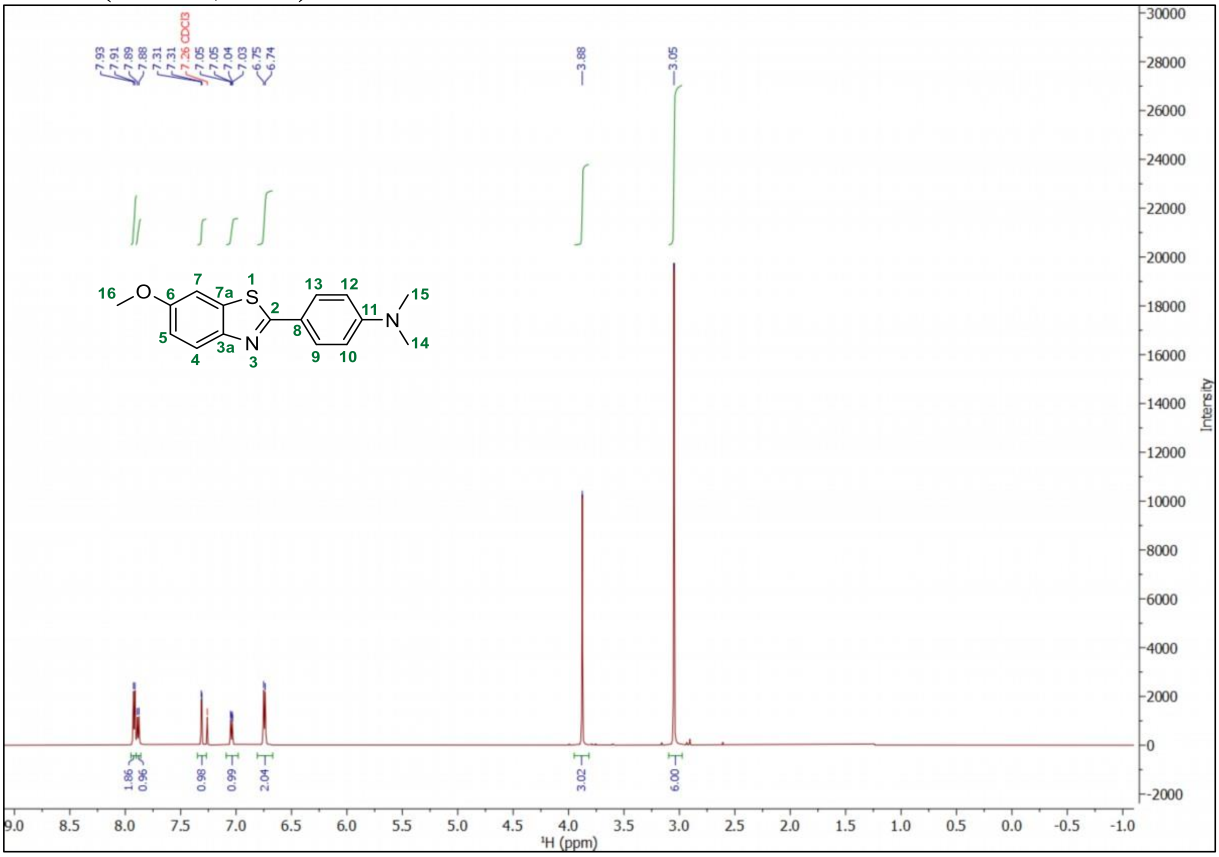

^{13}C NMR (151 MHz, $CDCl_3$)

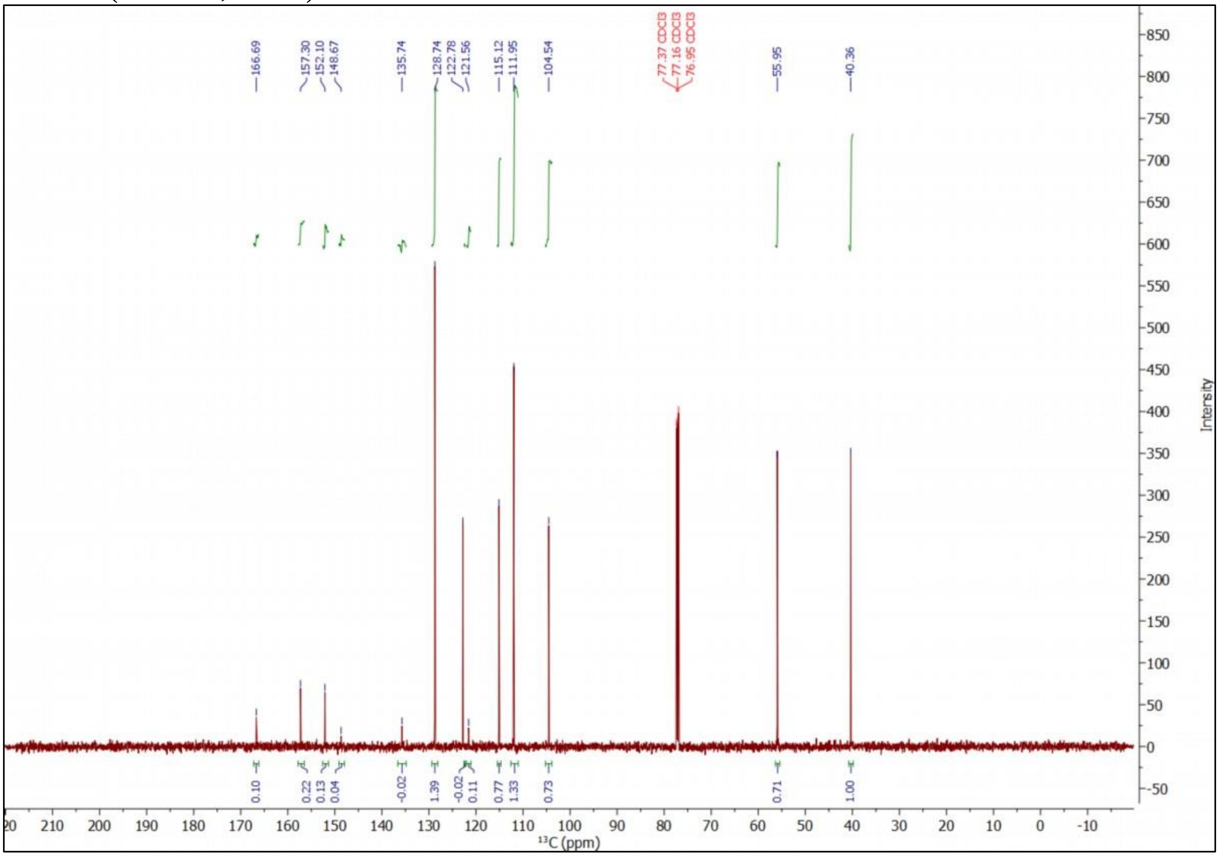

DEPT135 NMR (CDCl$_3$, **14**)

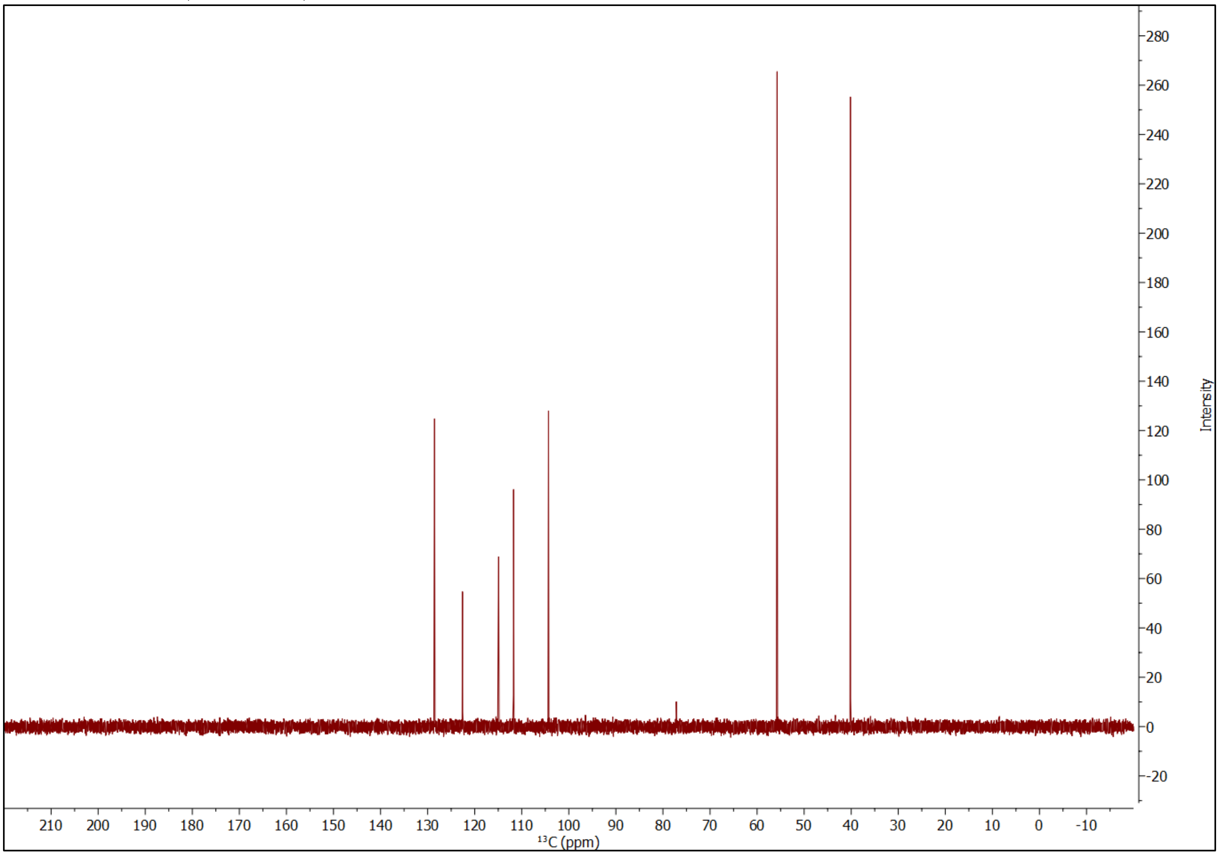

^{1}H-^{1}H-COSY NMR (CDCl$_3$, **14**)

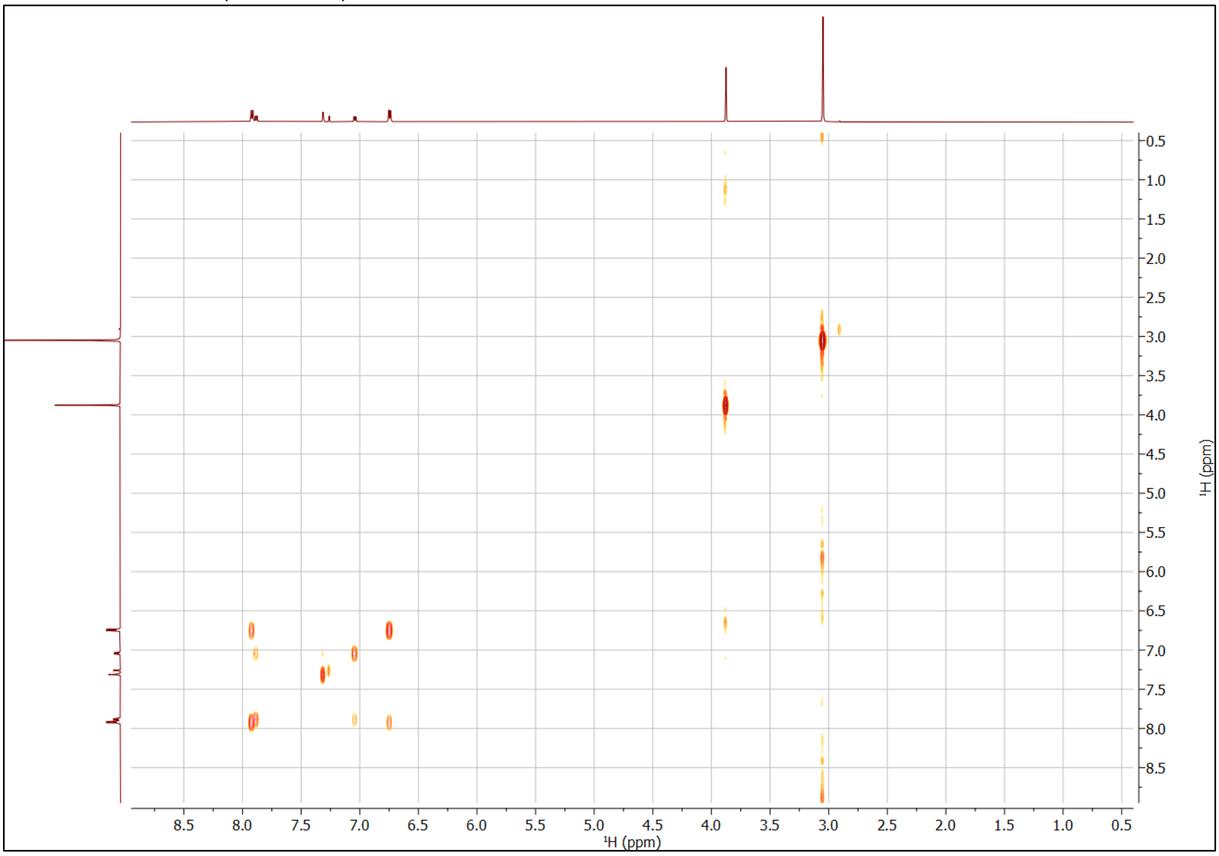

^{1}H-^{13}C-HSQC NMR ($CDCl_3$, **14**)

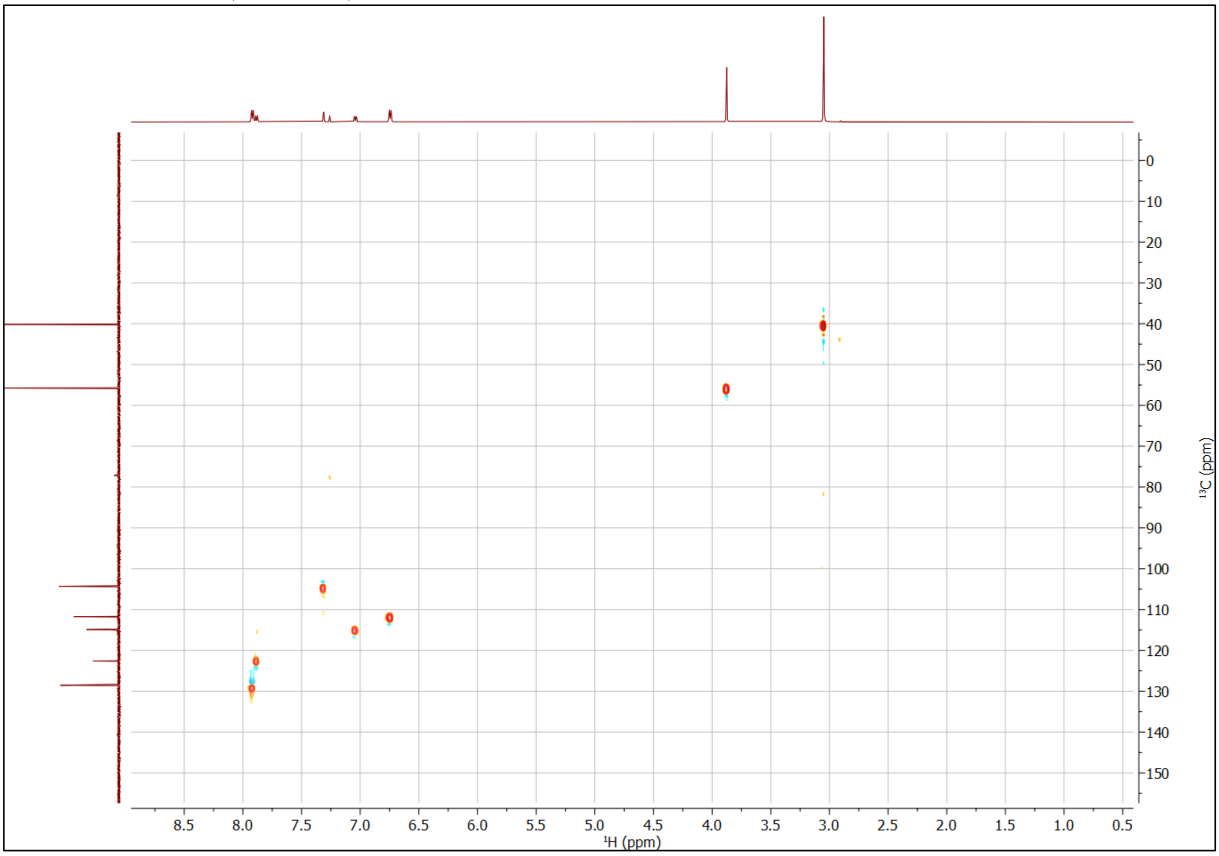

^{1}H-^{13}C-HMBC NMR ($CDCl_3$, **14**)

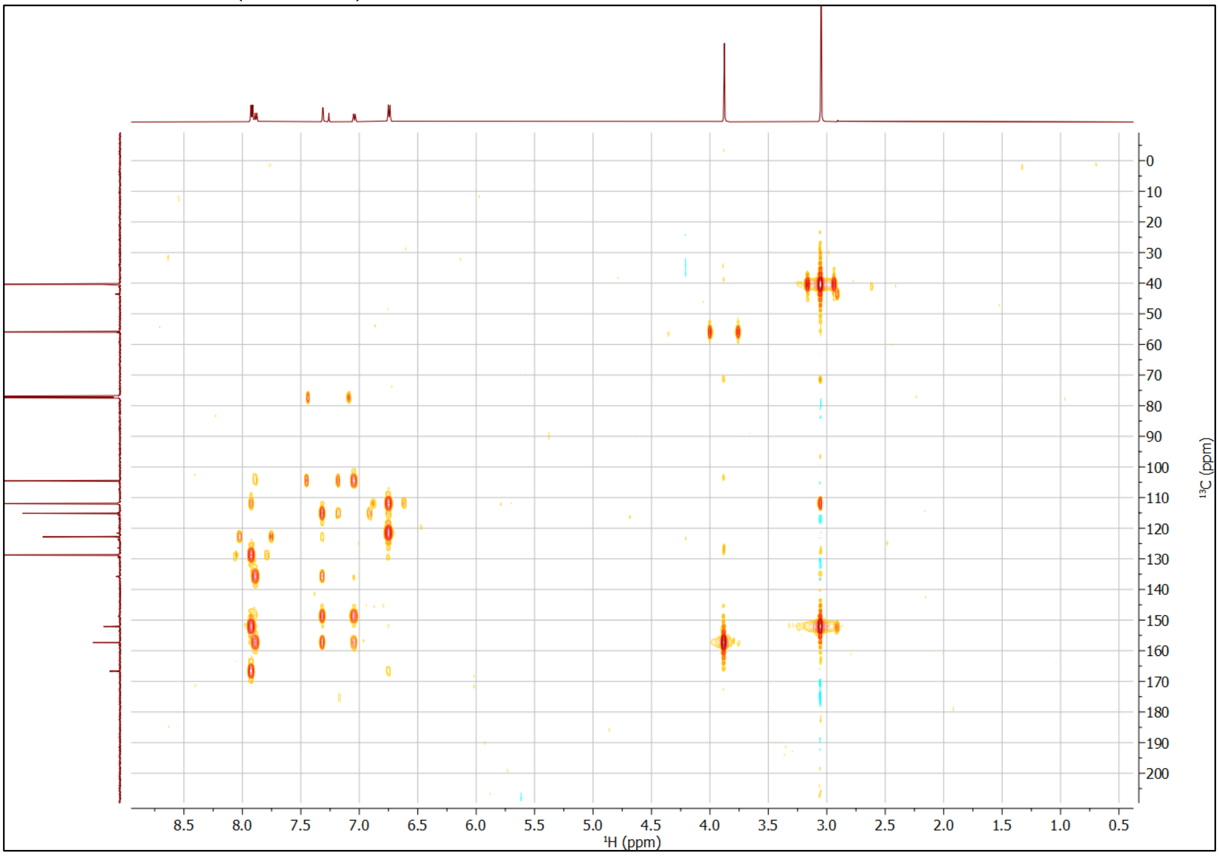

2-(4-(Dimethylamino)phenyl)benzo[*d*]thiazol-6-ol (**10**) [from **procedure A**]

^{1}H NMR (600 MHz, DMSO-d_6)

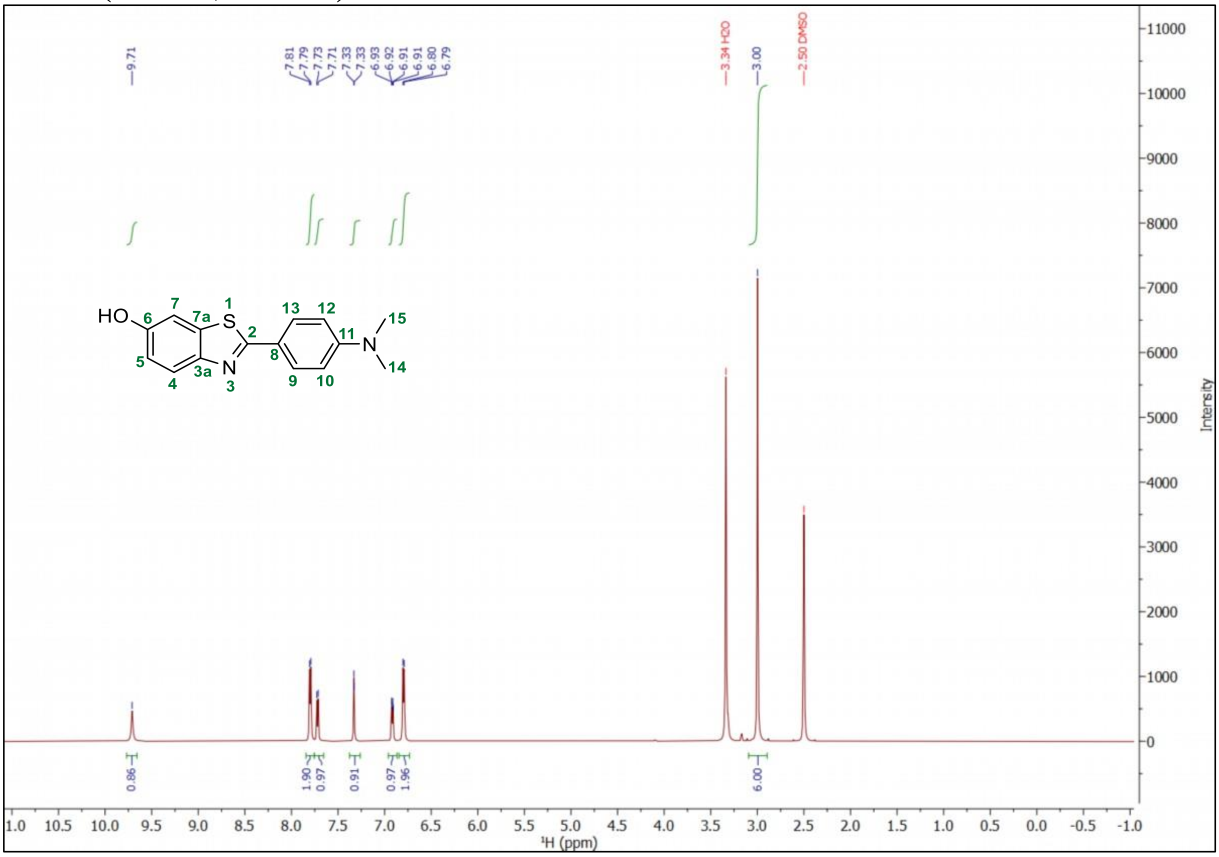

^{13}C NMR (151 MHz, DMSO-d_6)

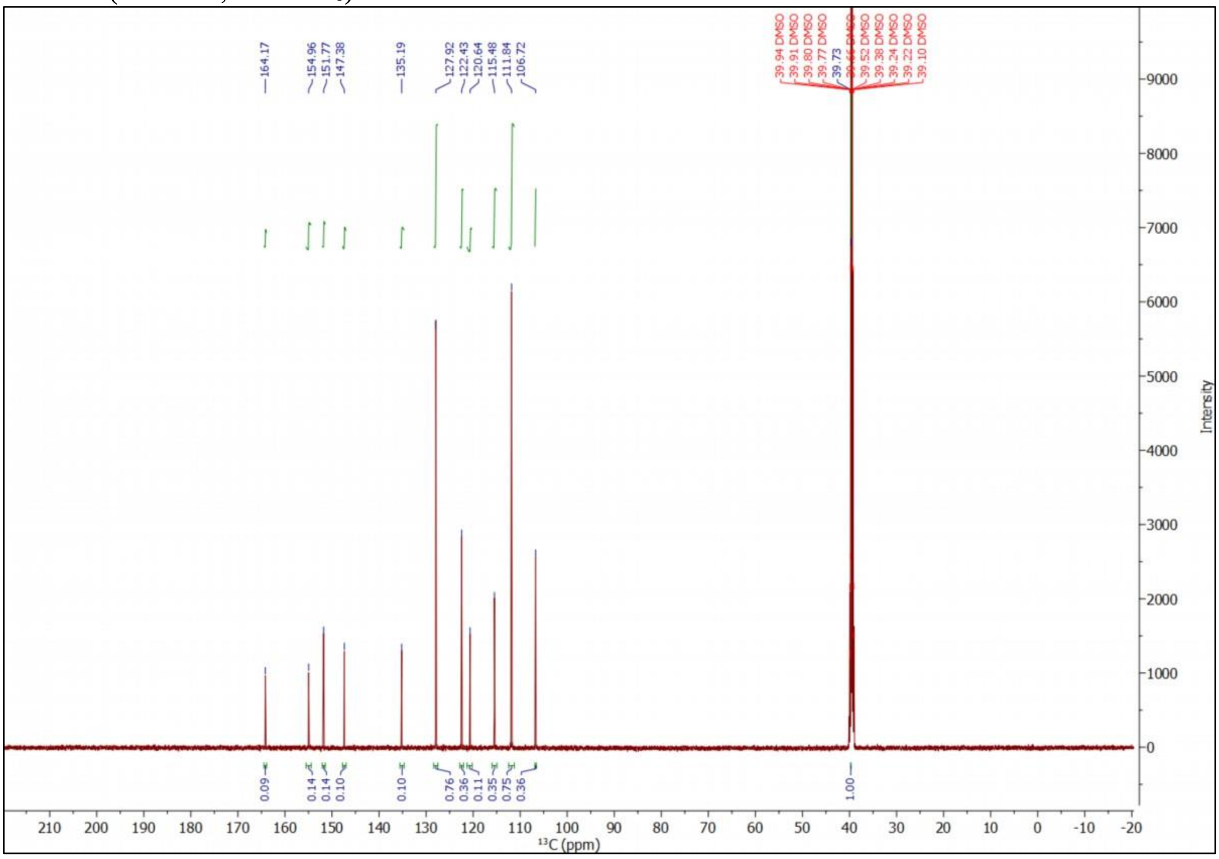

DEPT135 NMR (DMSO-d_6, **10**)

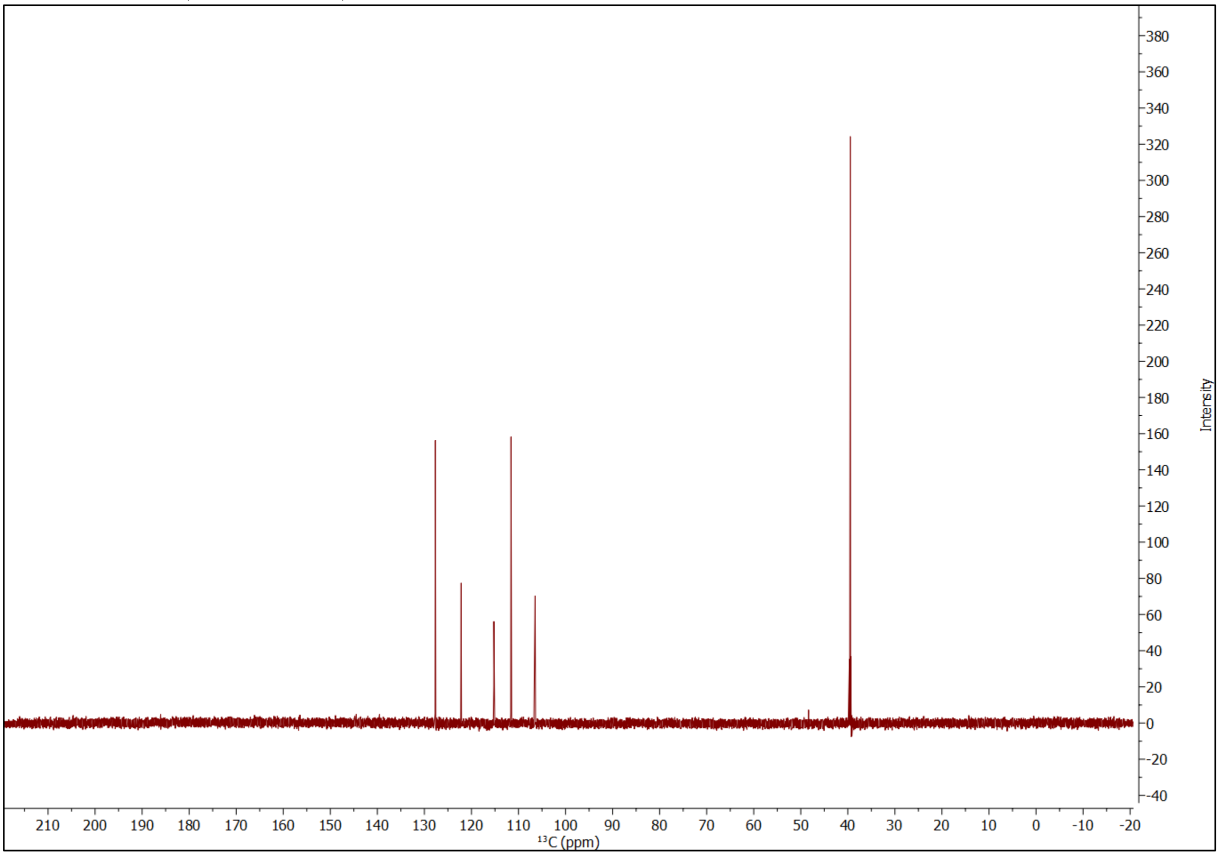

¹H-¹H-COSY NMR (DMSO-d_6, **10**)

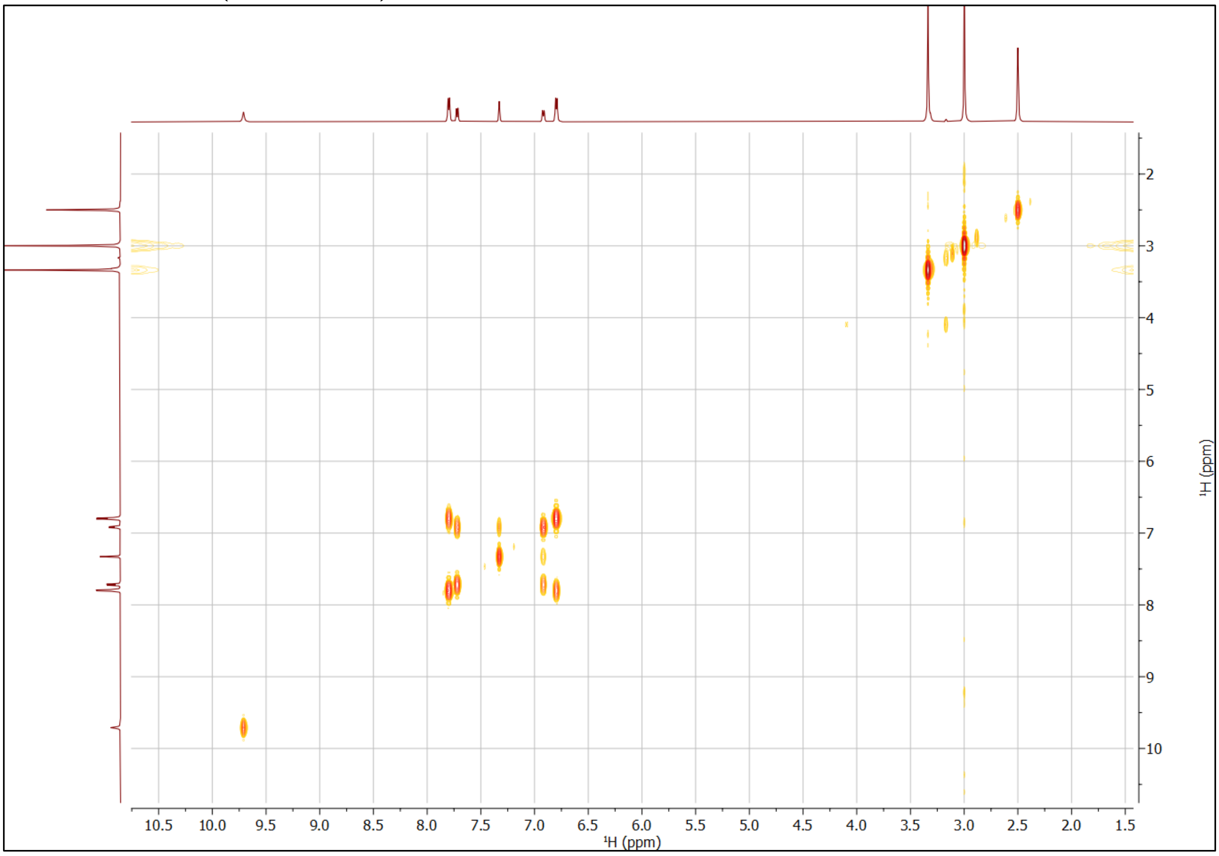

^{1}H-^{13}C-HSQC NMR (DMSO-d_6, **10**)

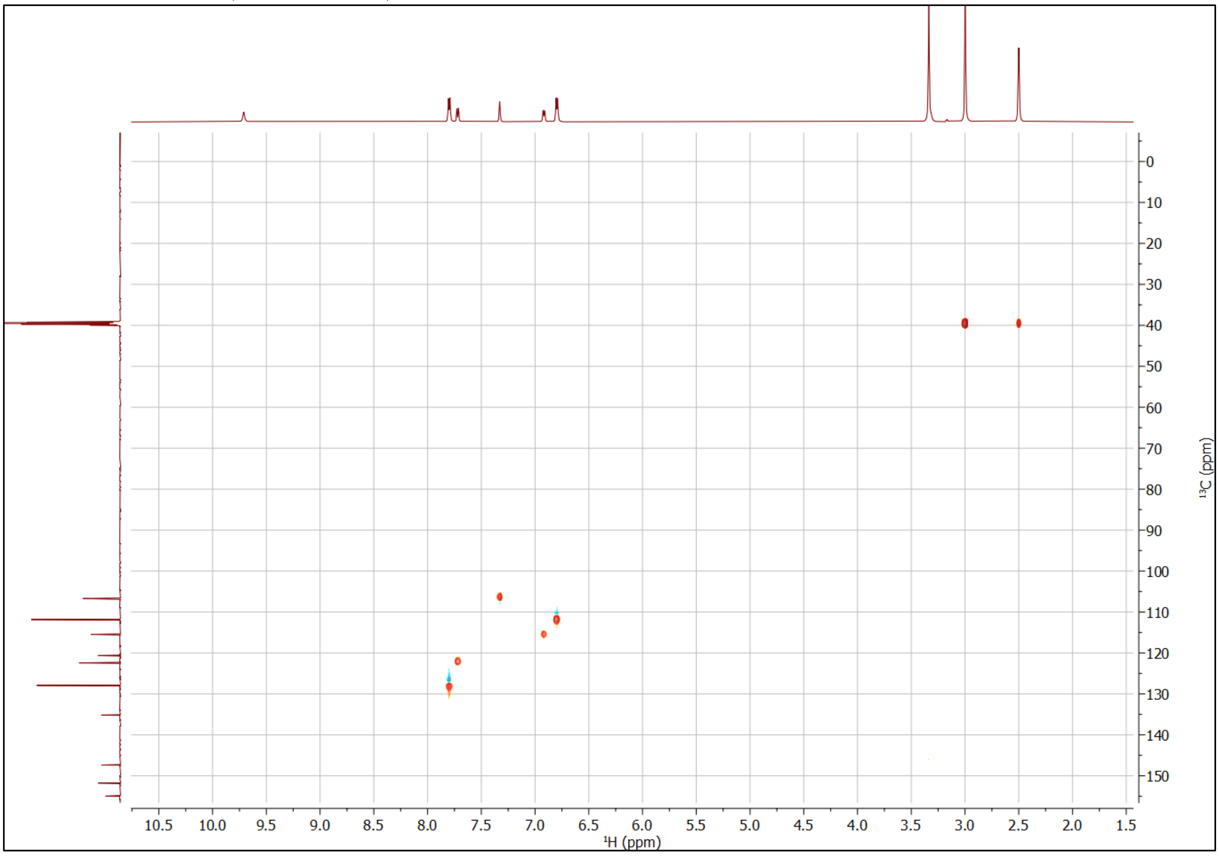

^{1}H-^{13}C-HMBC NMR (DMSO-d_6, **10**)

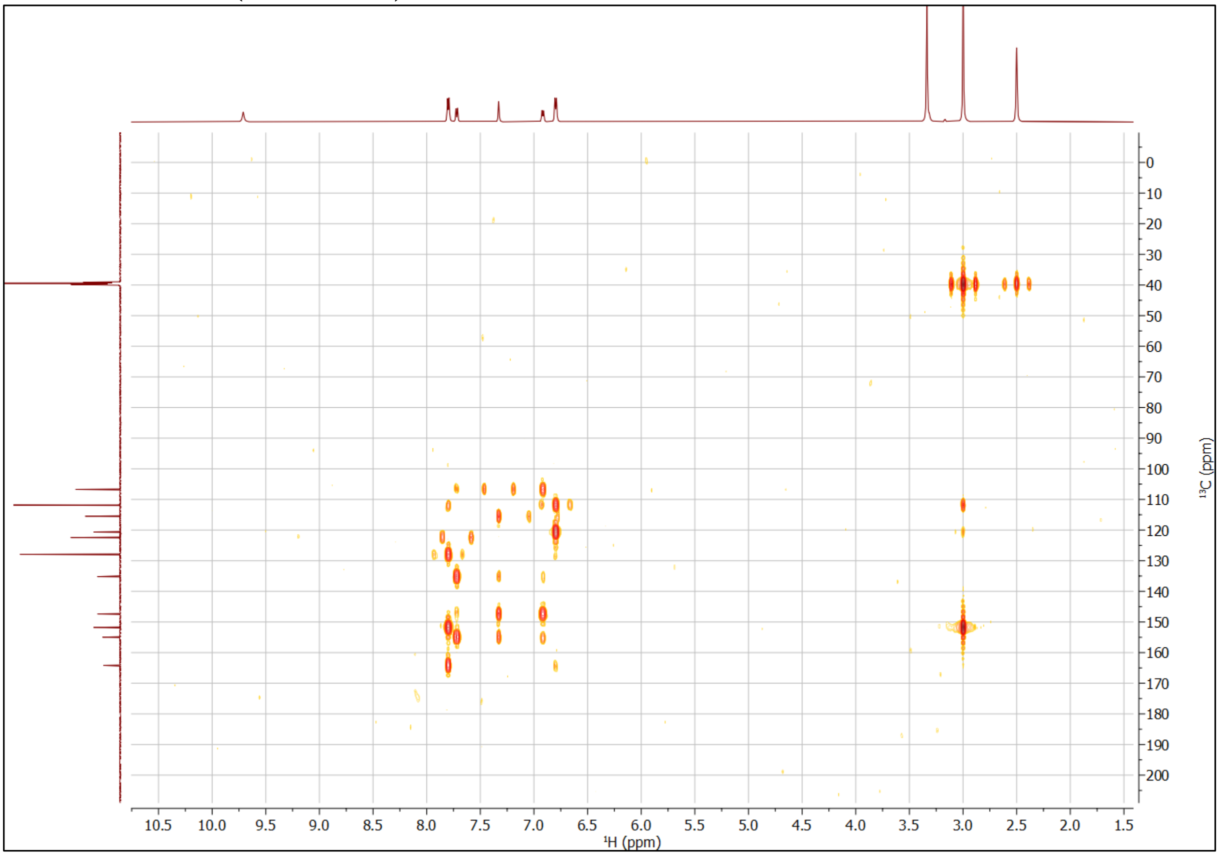

2-(4-(Dimethylamino)phenyl)benzo[*d*]thiazol-6-ol (**10**) [from **procedure B**]

^{1}H NMR (600 MHz, DMSO-d_6)

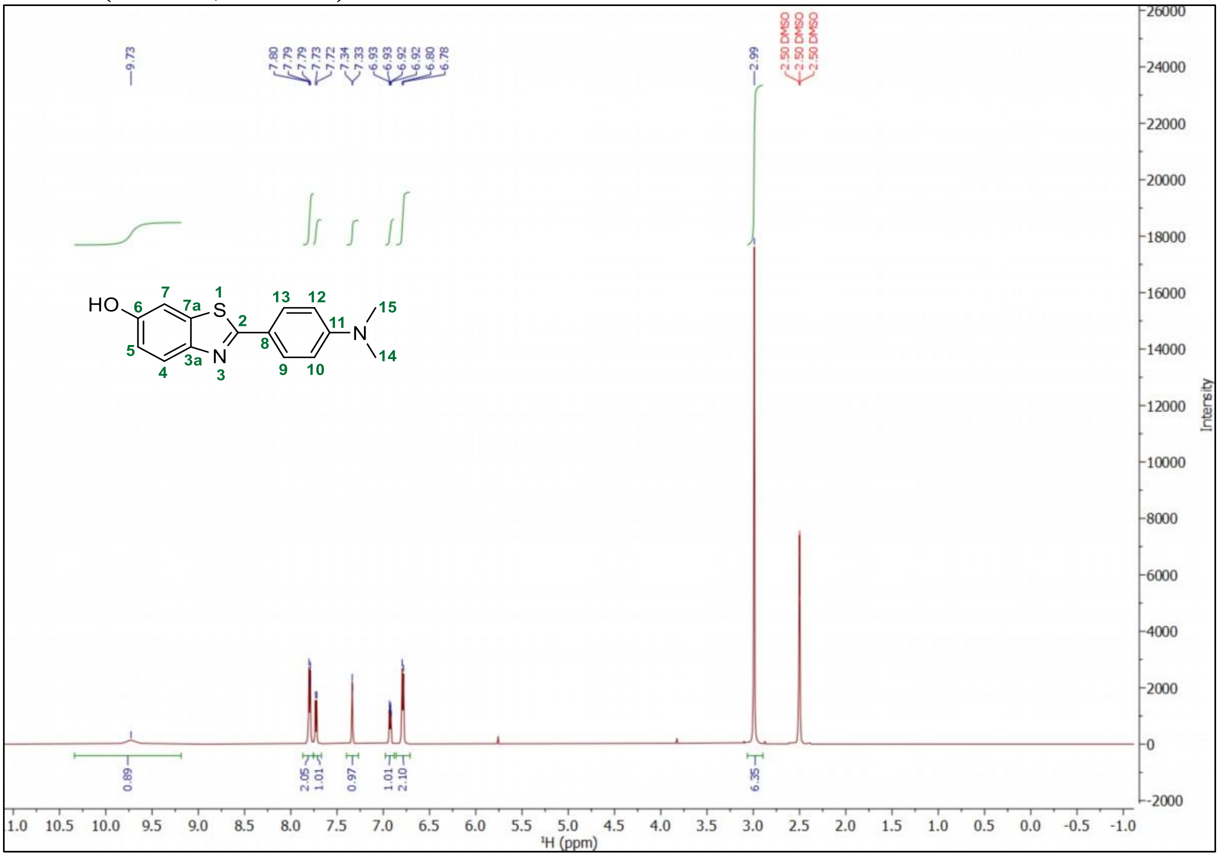

^{13}C NMR (151 MHz, DMSO-d_6)

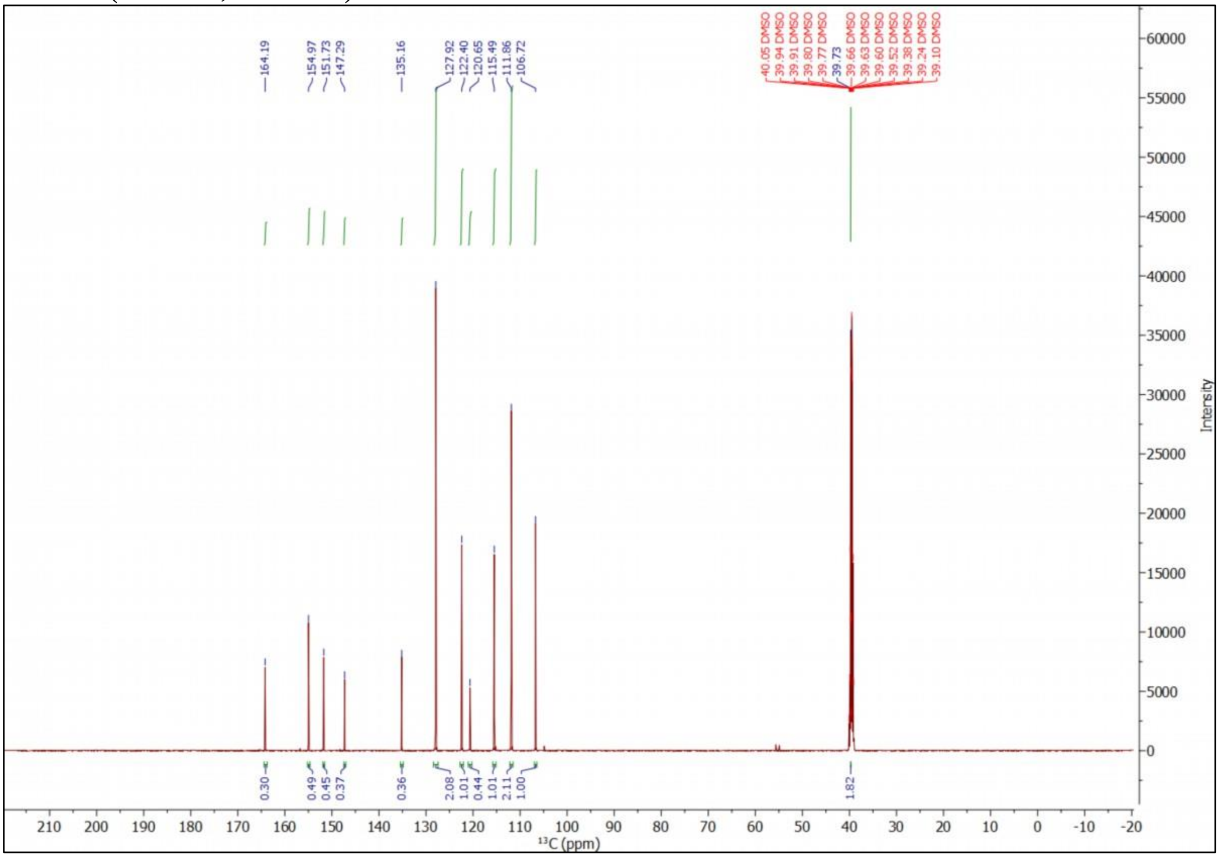

DEPT135 NMR (DMSO-d_6, **10**)

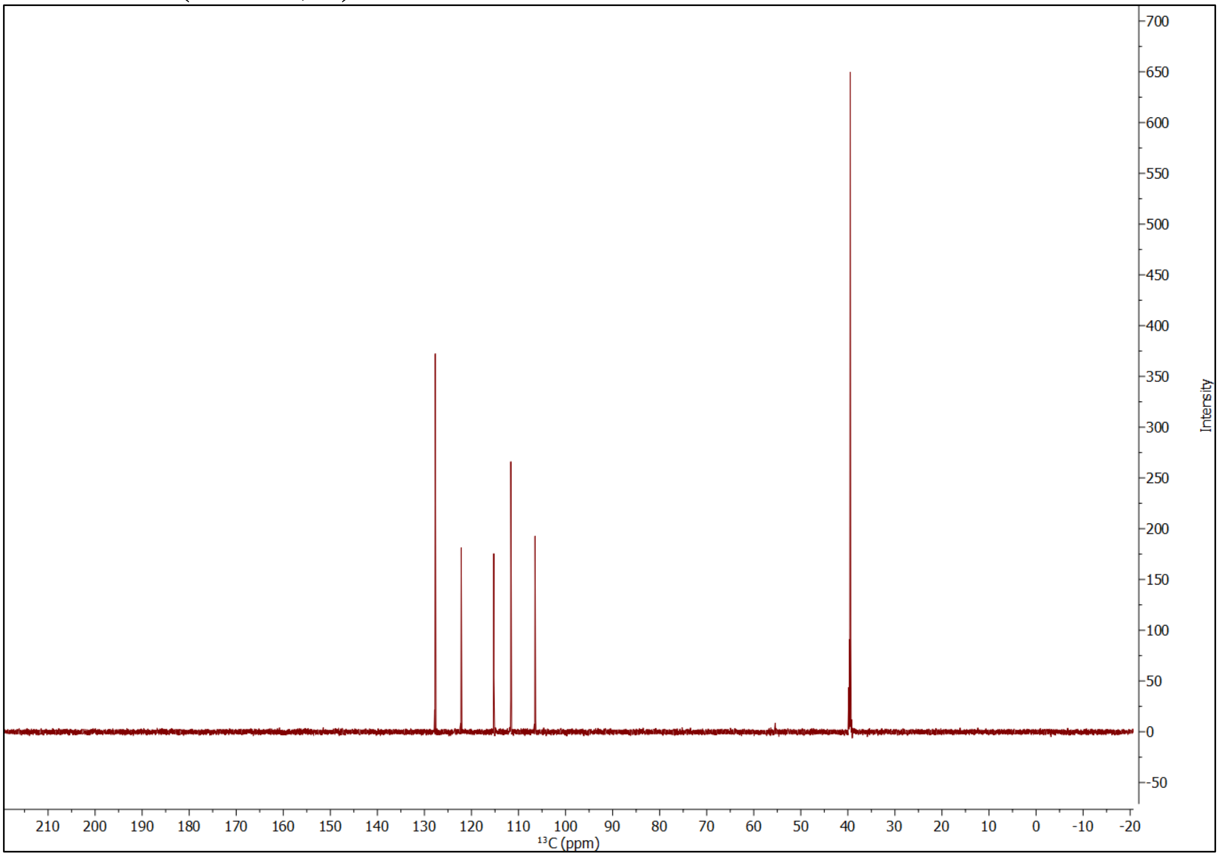

^{1}H-^{1}H-COSY NMR (DMSO-d_6, **10**)

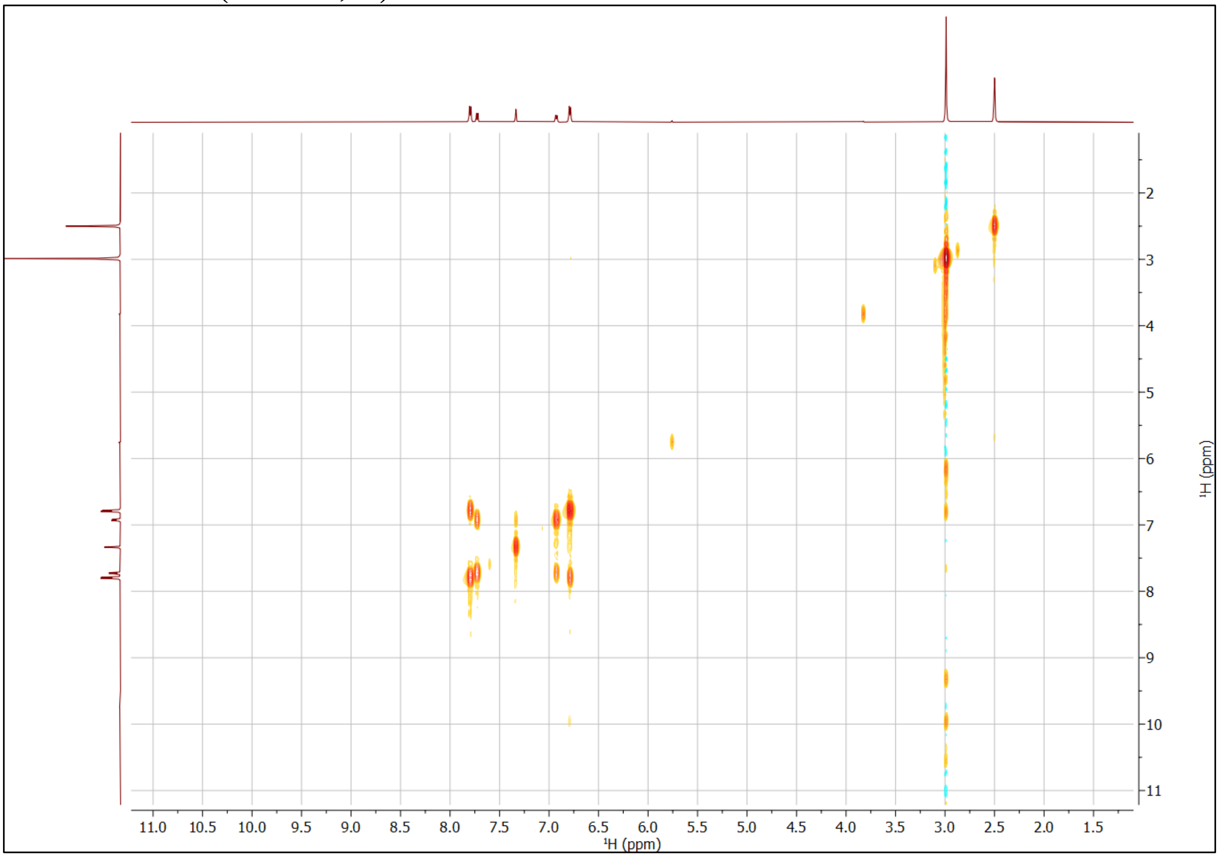

^{1}H-^{13}C-HSQC NMR (DMSO-d_6, **10**)

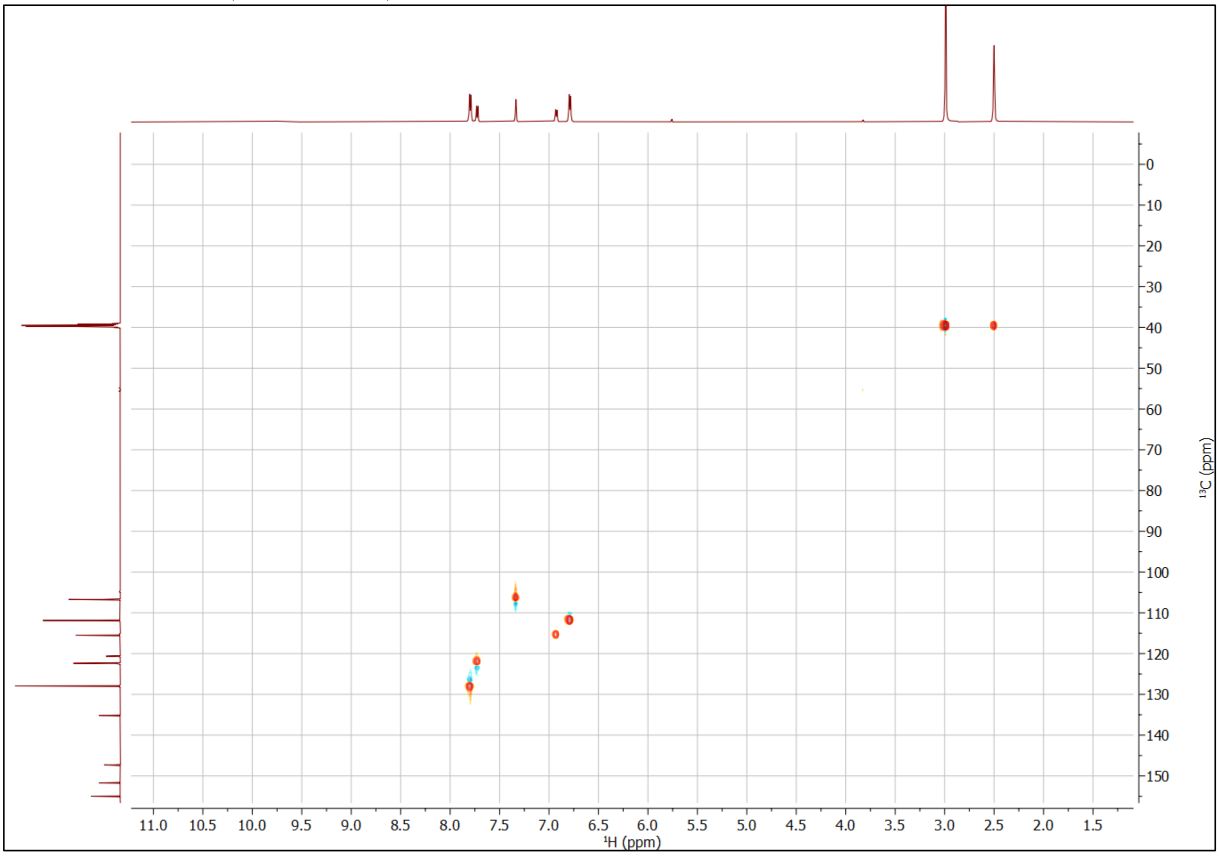

^{1}H-^{13}C-HMBC NMR (DMSO-d_6, **10**)

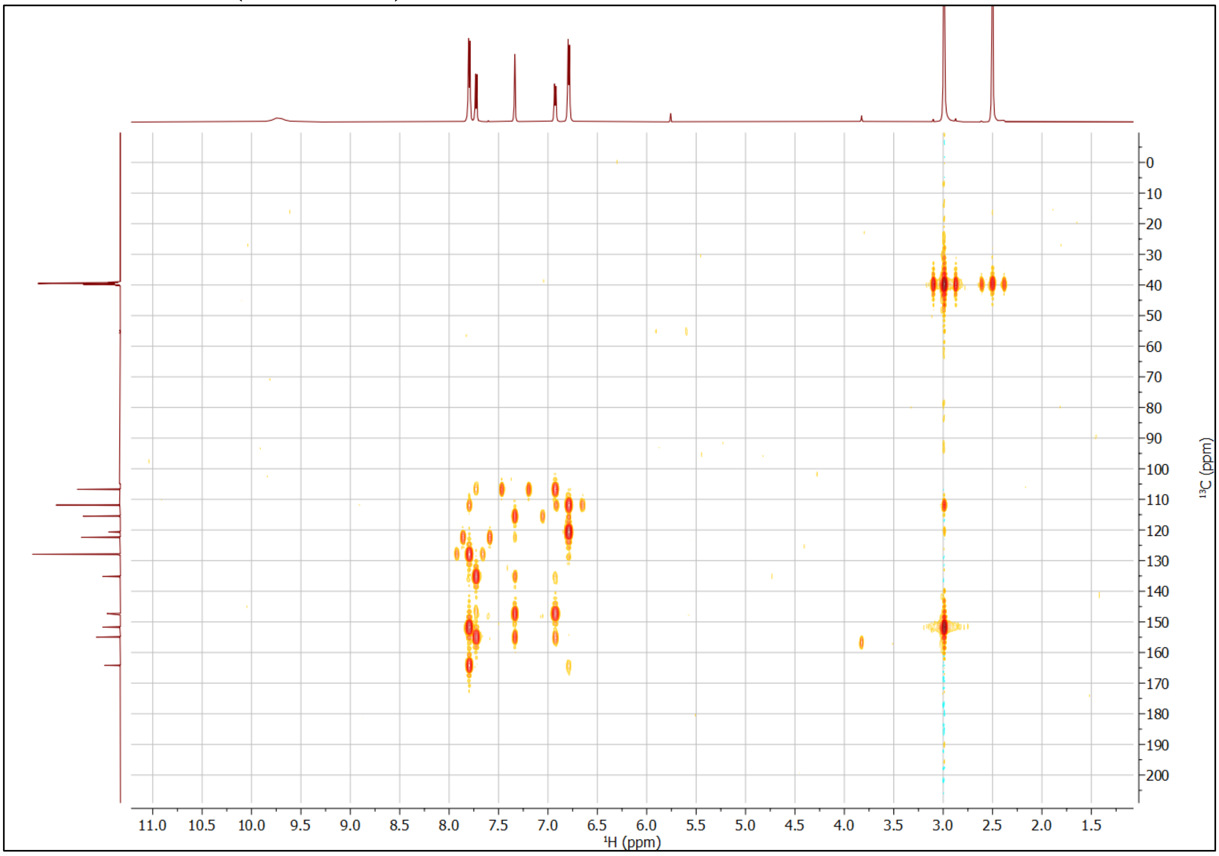

3-(2-(2-Bromoethoxy)ethoxy)prop-1-yne (**16**)

^1H NMR (600 MHz, $CDCl_3$)

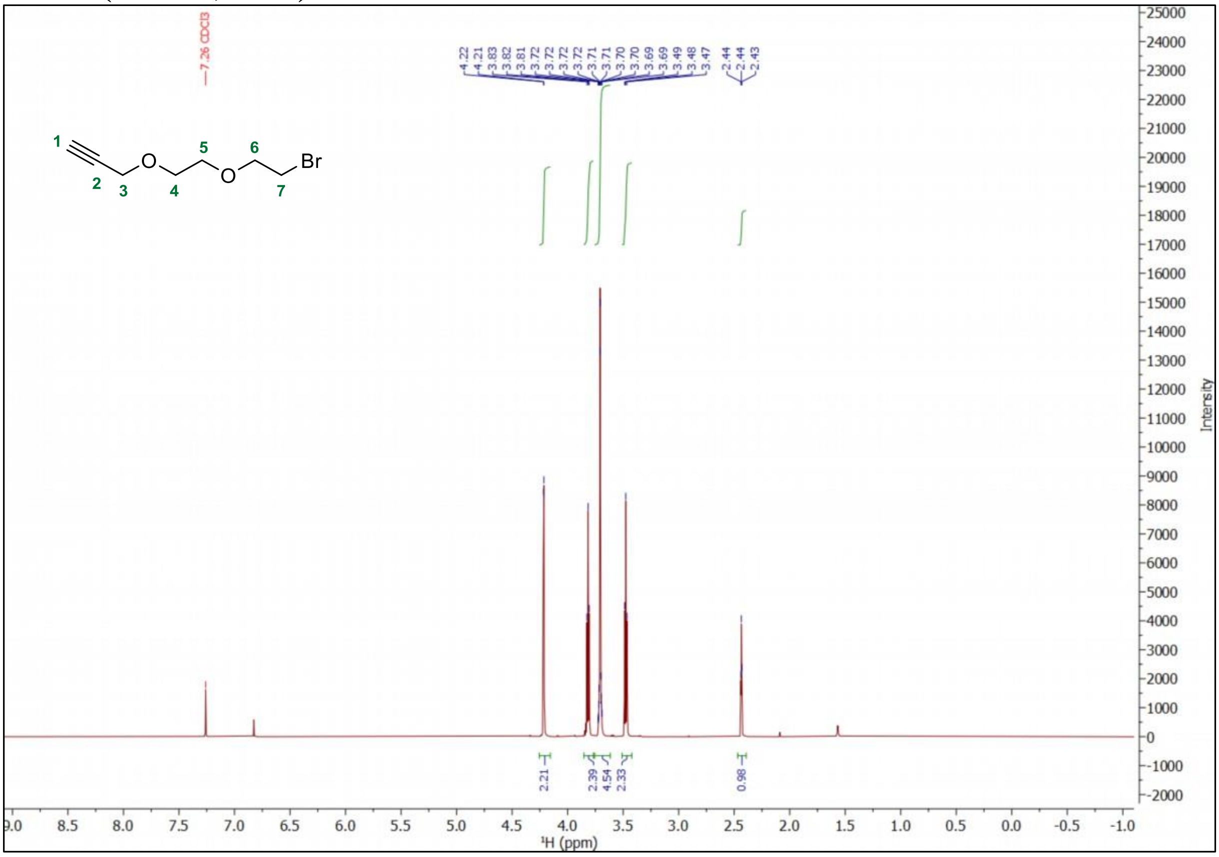

^{13}C NMR (151 MHz, $CDCl_3$)

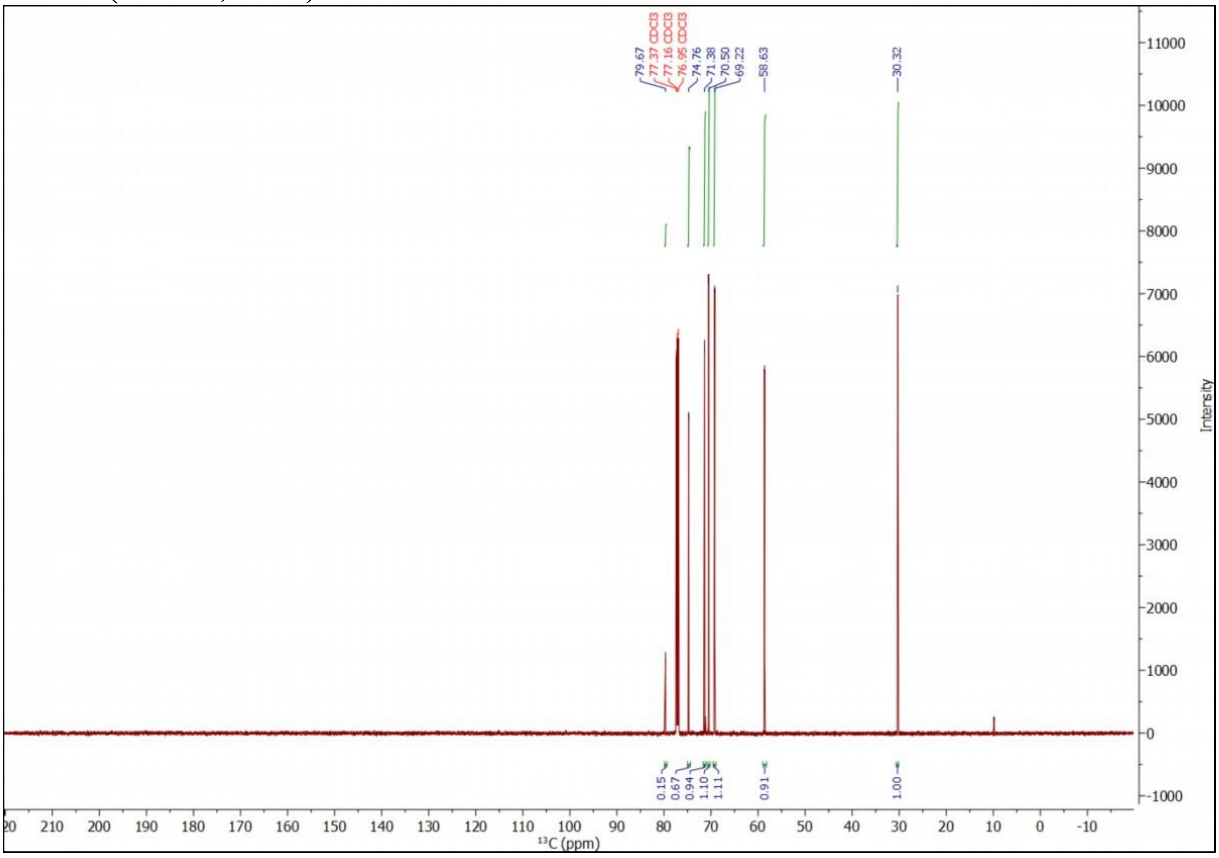

DEPT135 NMR ($CDCl_3$, **16**)

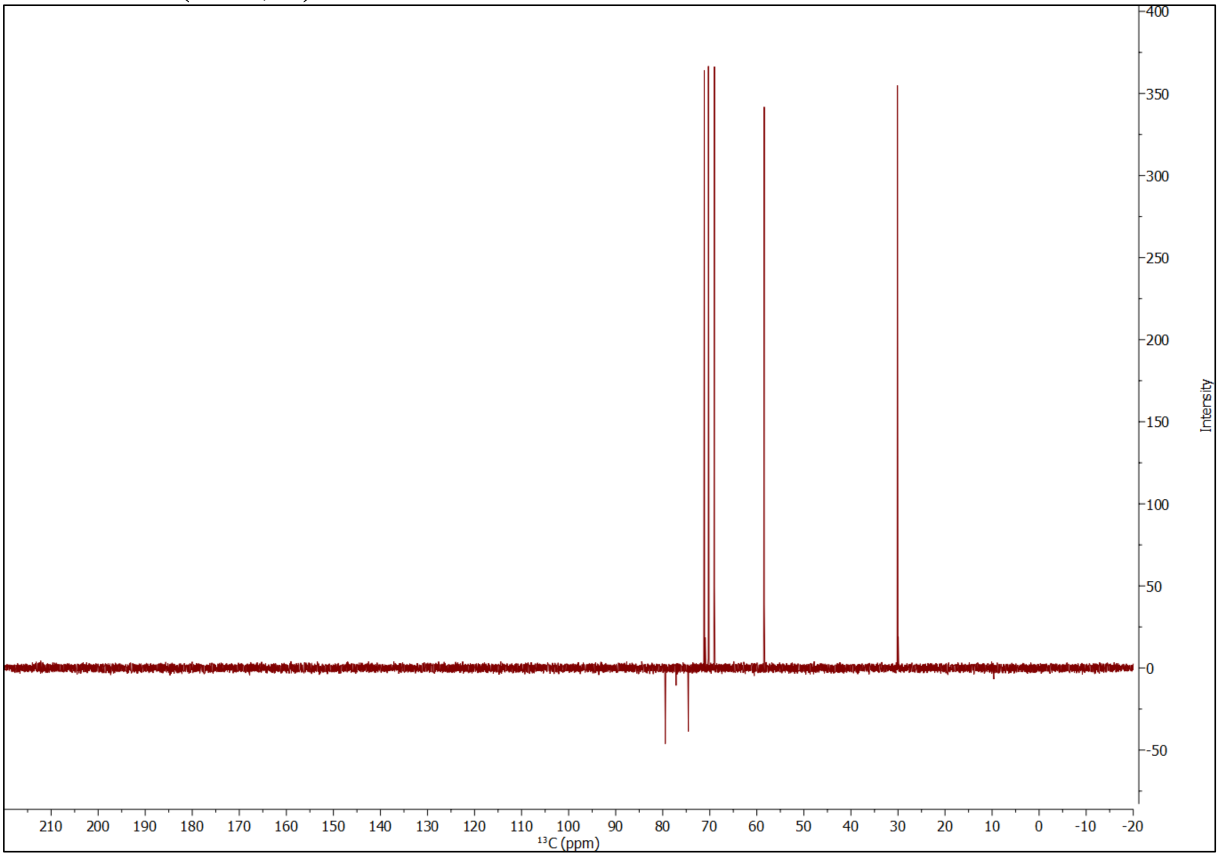

^{1}H-^{1}H-COSY NMR ($CDCl_3$, **16**)

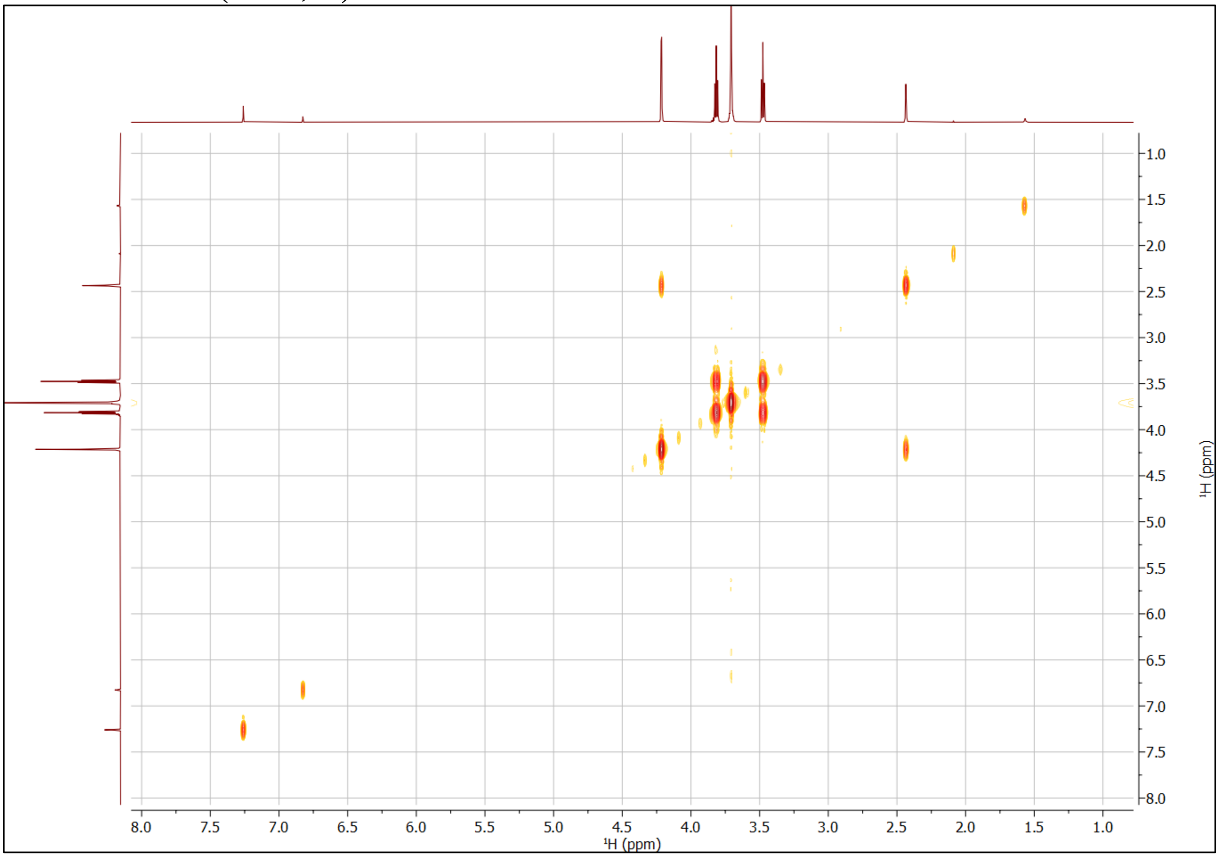

^{1}H-^{13}C-HSQC NMR ($CDCl_3$, **16**)

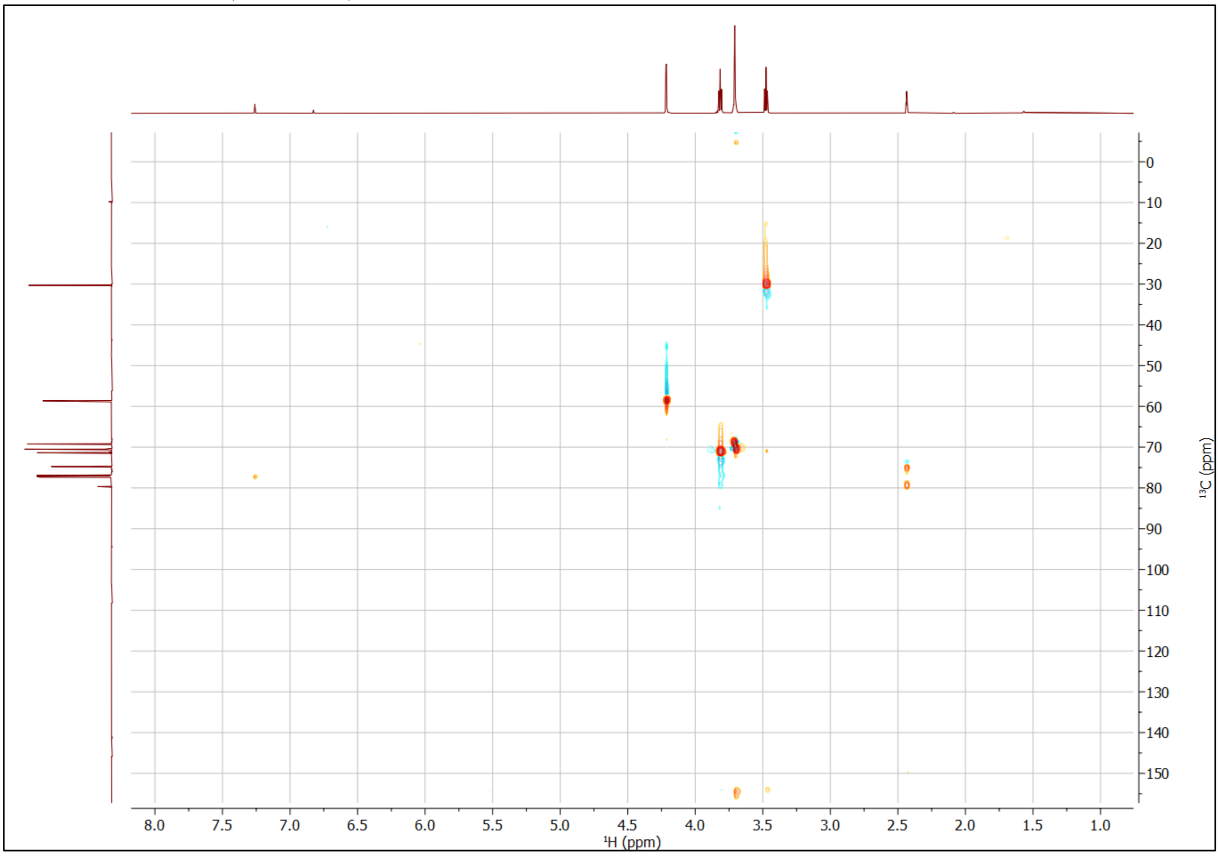

^{1}H-^{13}C-HMBC NMR ($CDCl_3$, **16**)

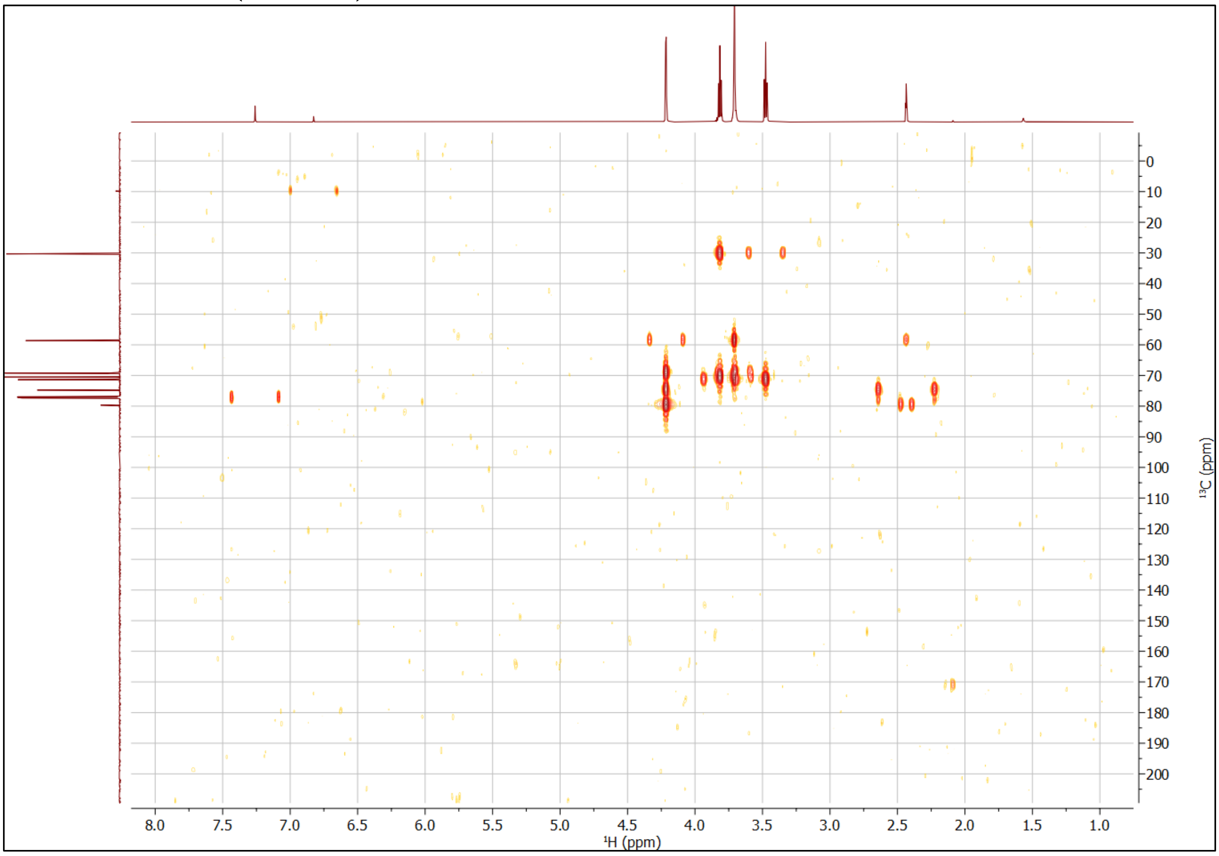

N,*N*-Dimethyl-4-(6-(2-(2-(prop-2-yn-1-yloxy)ethoxy)ethoxy)benzo[*d*]thiazol-2-yl)aniline (**17**)

^{1}H NMR (600 MHz, $CDCl_3$)

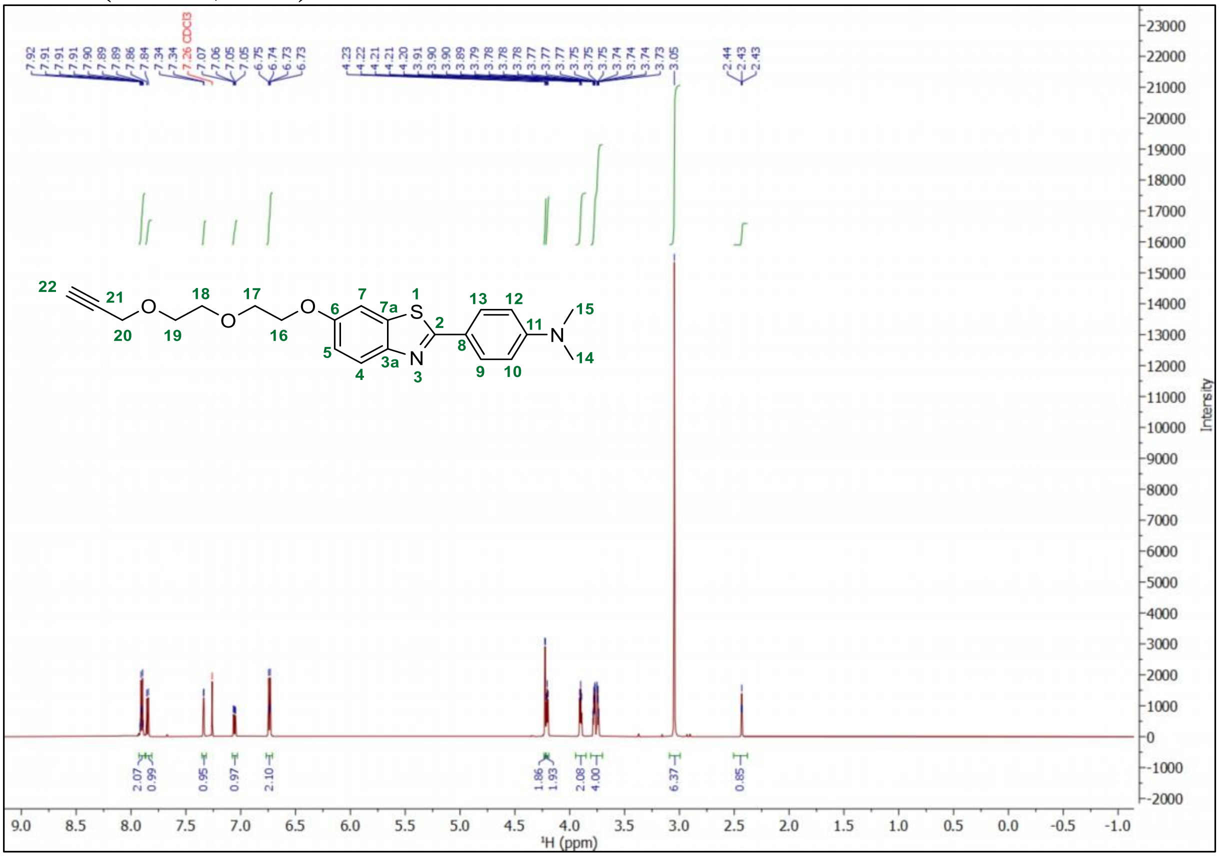

^{13}C NMR (151 MHz, $CDCl_3$)

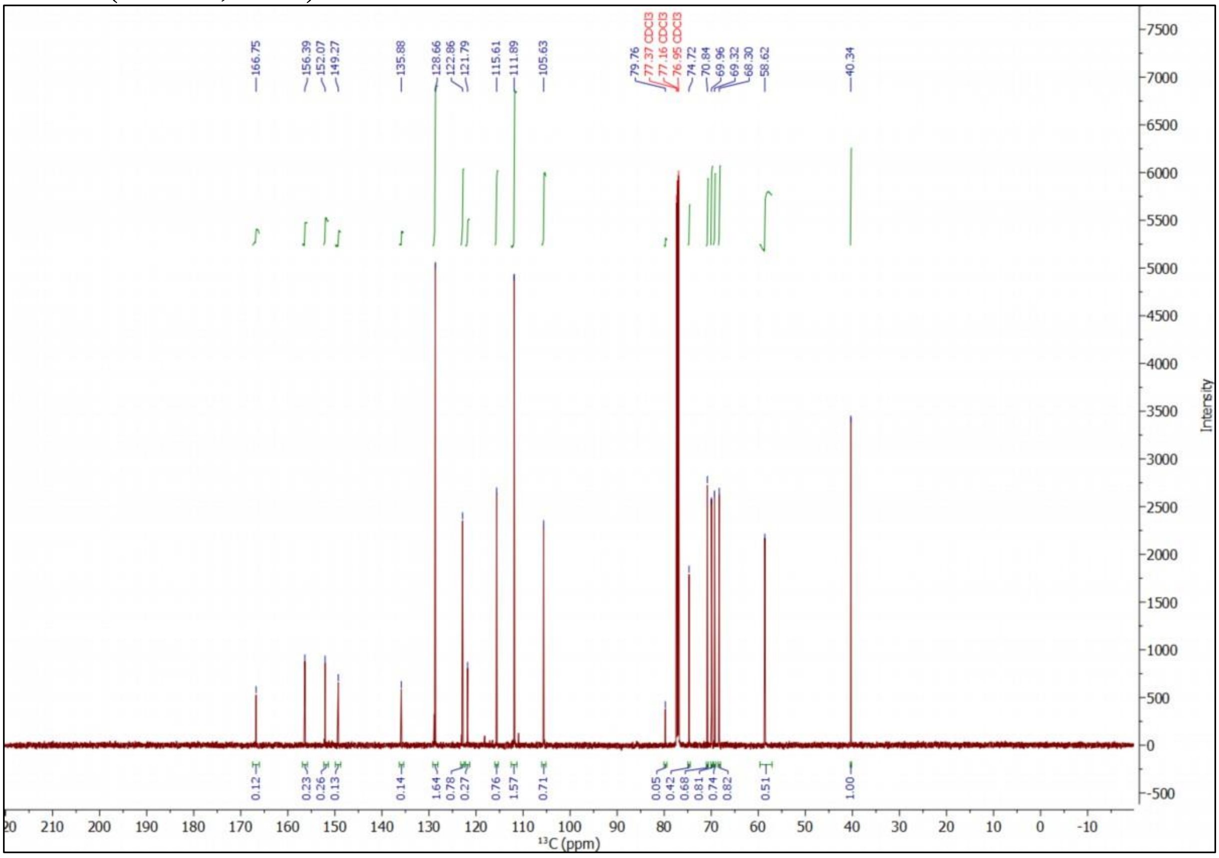

DEPT135 NMR ($CDCl_3$, **17**)

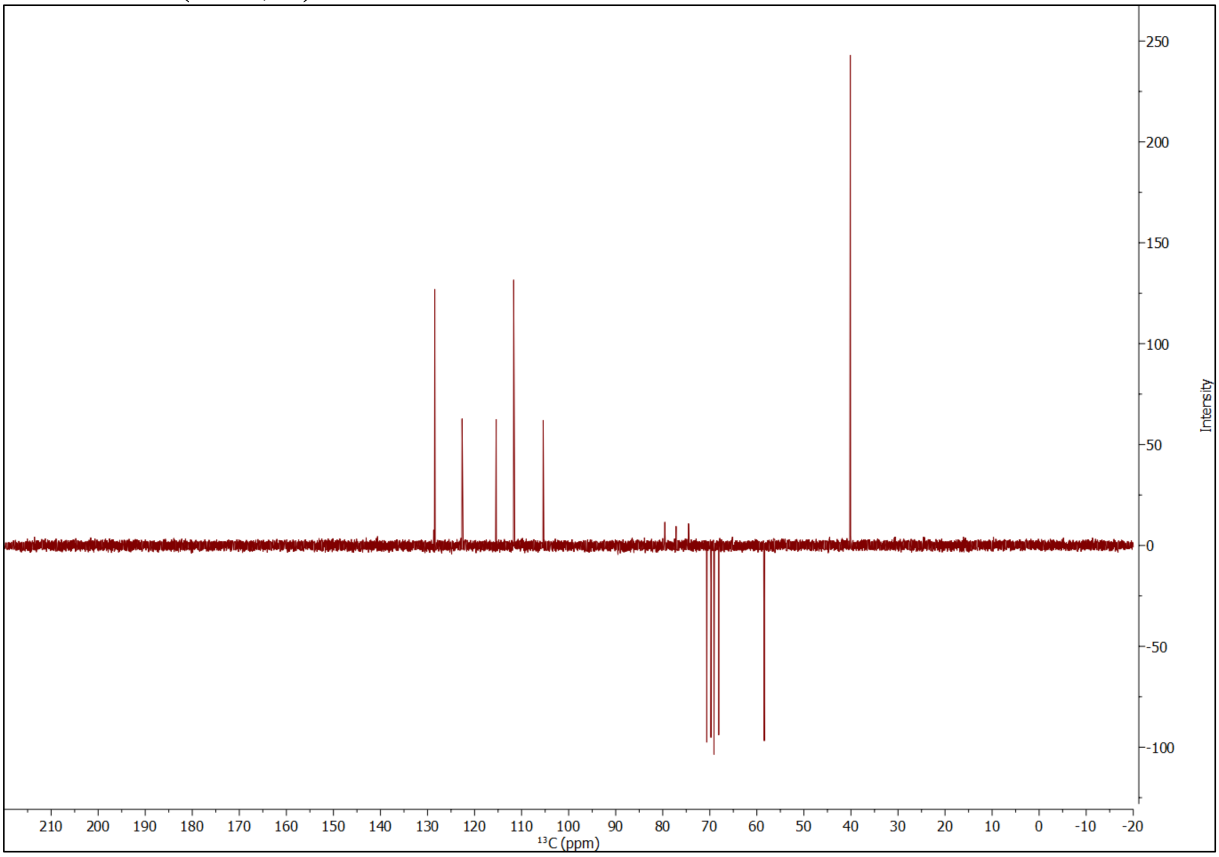

^{1}H-^{1}H-COSY NMR ($CDCl_3$, **17**)

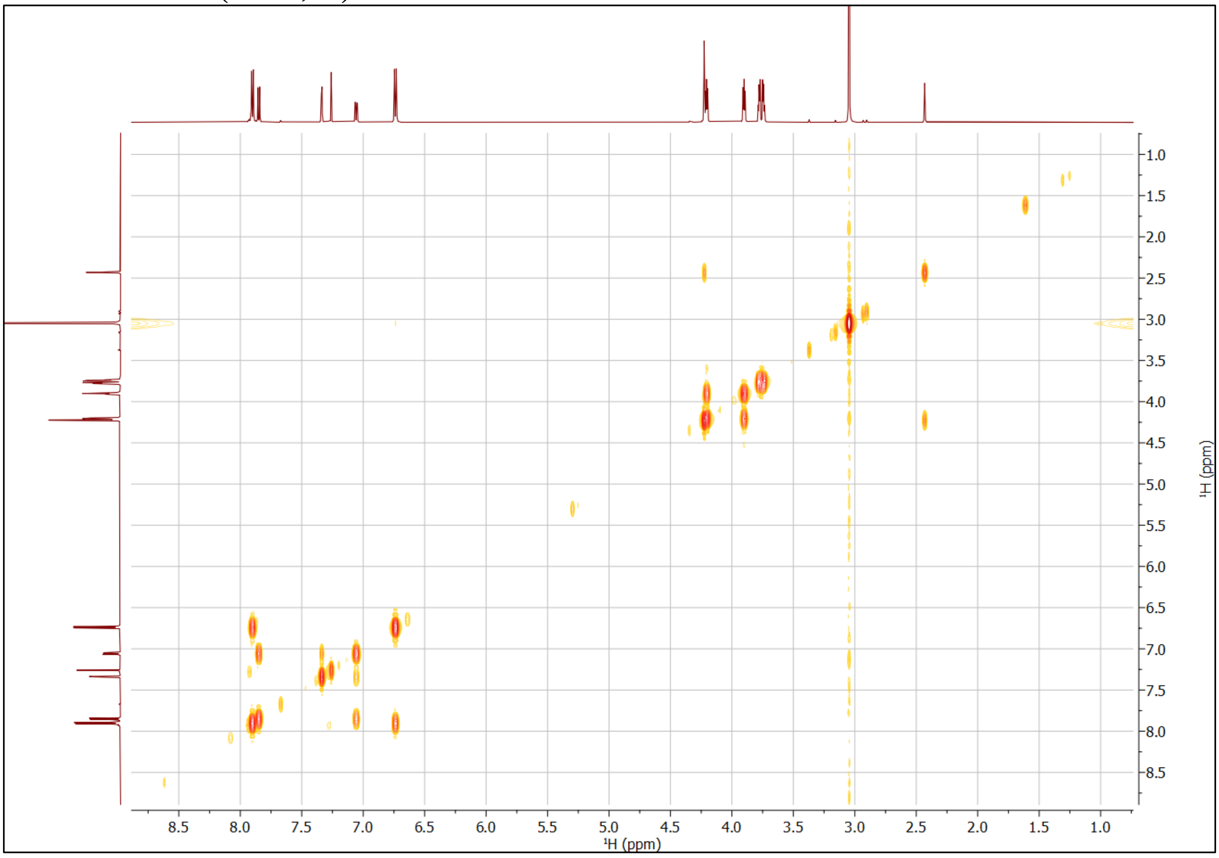

^{1}H-^{13}C-HSQC NMR ($CDCl_3$, **17**)

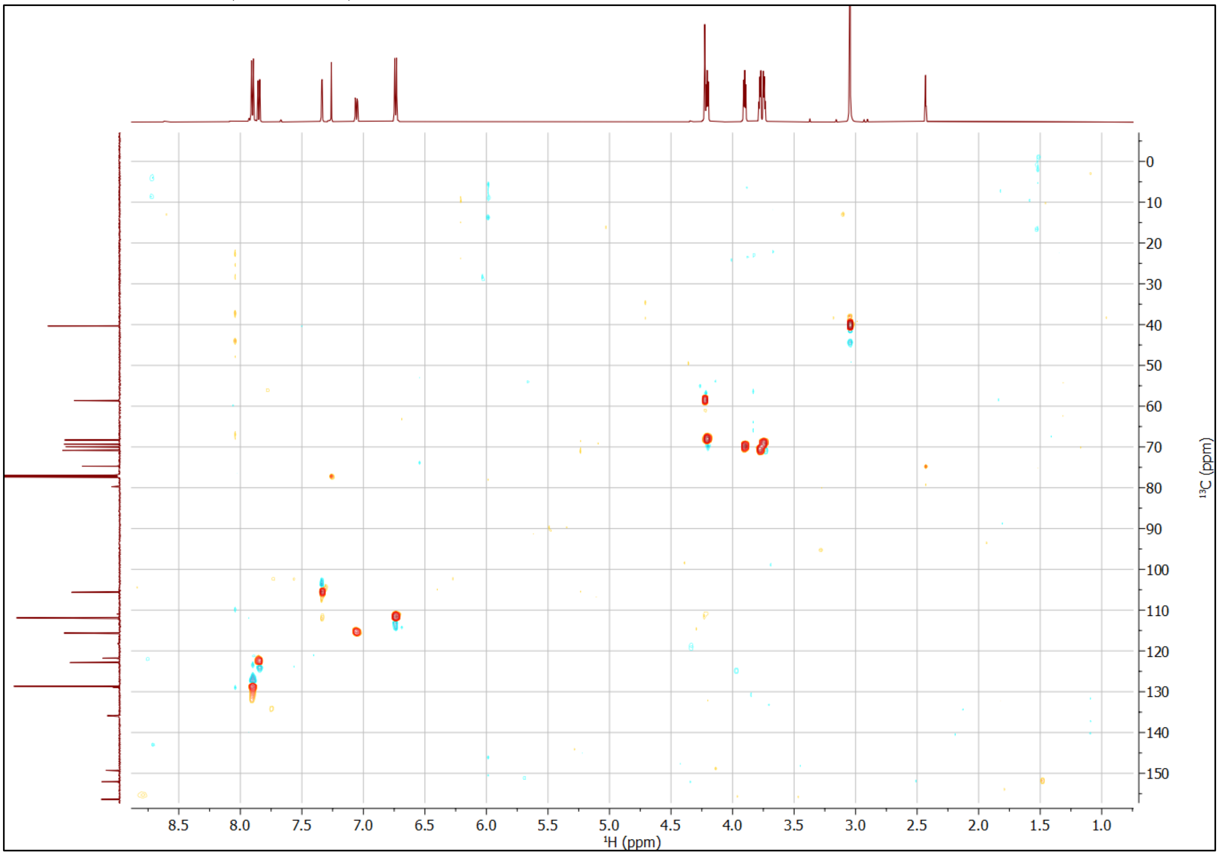

^{1}H-^{13}C-HMBC NMR ($CDCl_3$, **17**)

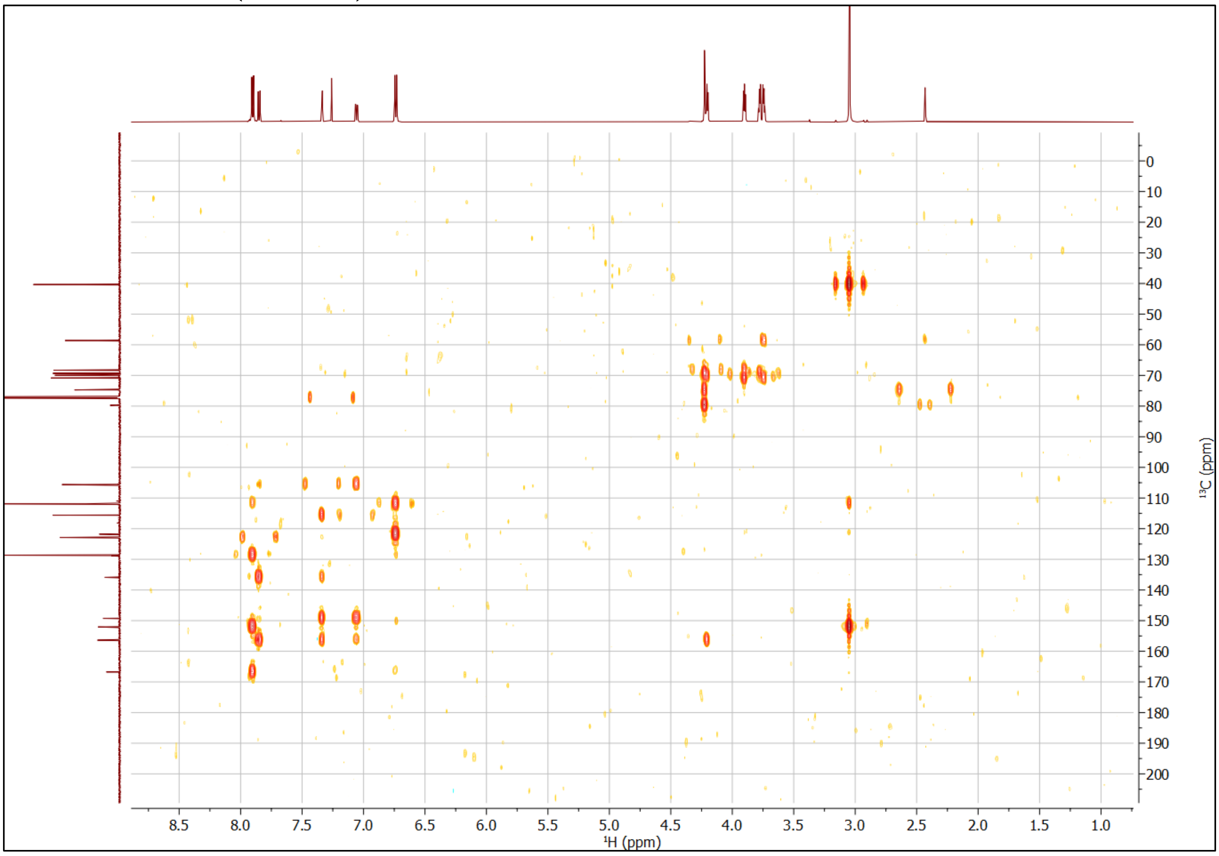

N,*N*-Bis(2-(2-(4-((2-(2-((2-(4-(dimethylamino)phenyl)benzo[*d*]thiazol-6-yl)oxy)ethoxy)ethoxy)methyl)-1*H*-1,2,3-triazol-1-yl)ethoxy)ethyl)-5-((3a*S*,4*S*,6a*R*)-2-oxohexahydro-1*H*-thieno[3,4-*d*]imidazol-4-yl)pentanamide (**18**, cCAP-1)

^{1}H NMR (600 MHz, $CDCl_3$)

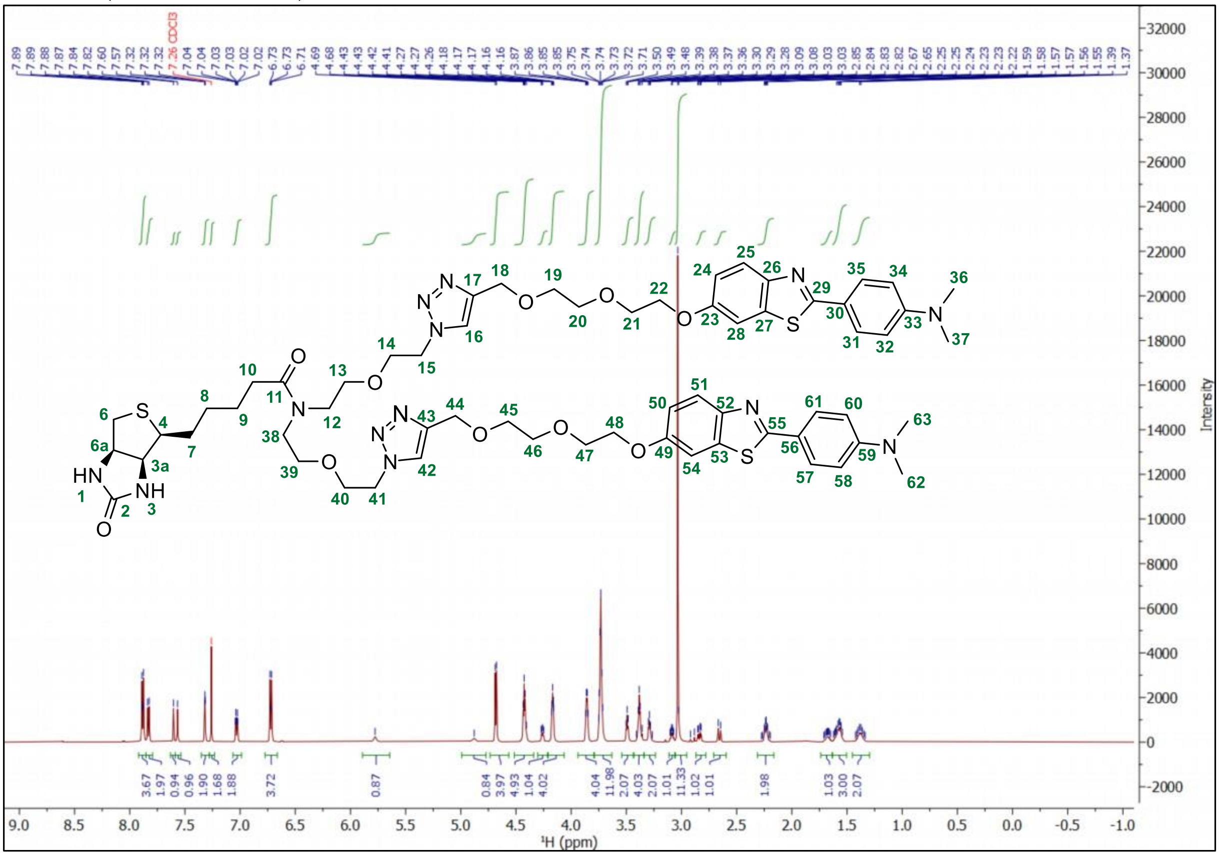

^{13}C NMR (151 MHz, $CDCl_3$)

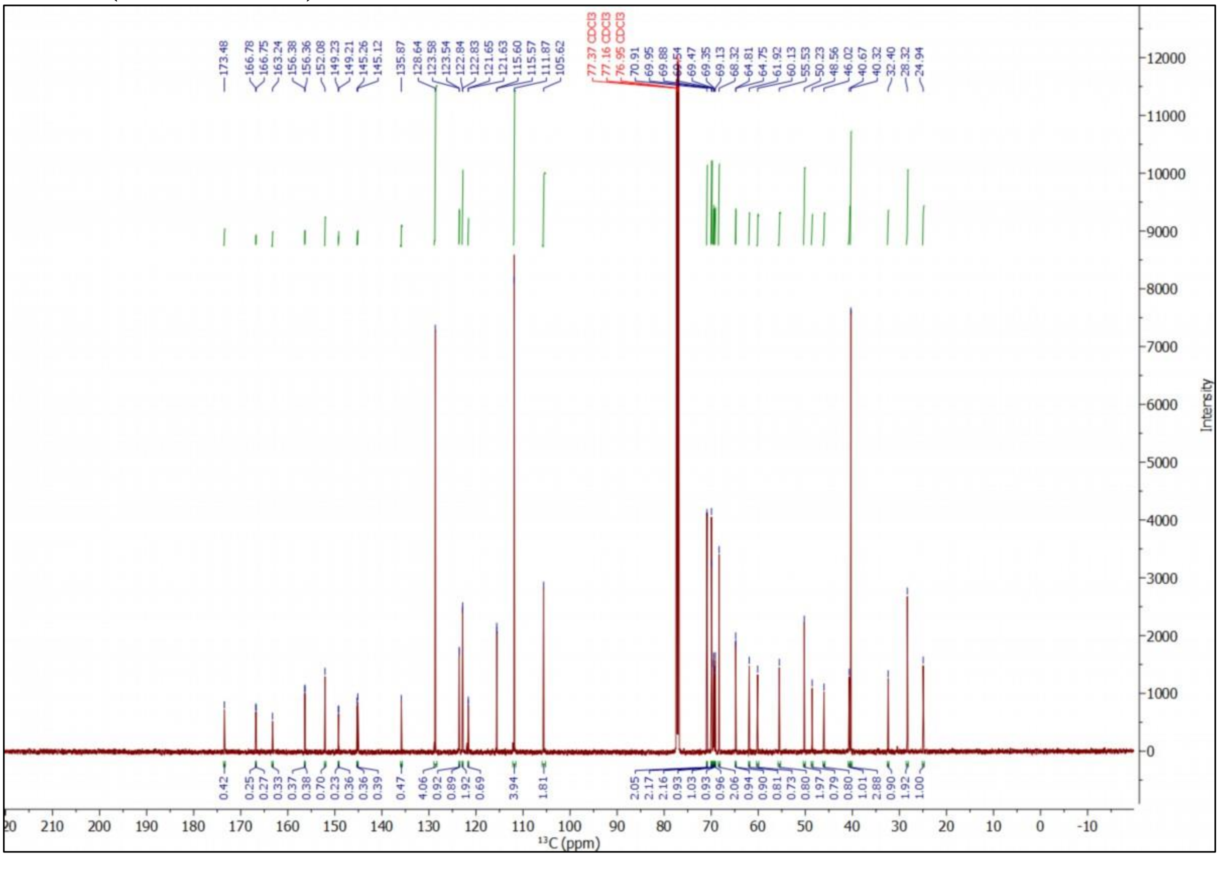

DEPT135 NMR (CDCl$_3$, **18**, cCAP-1)

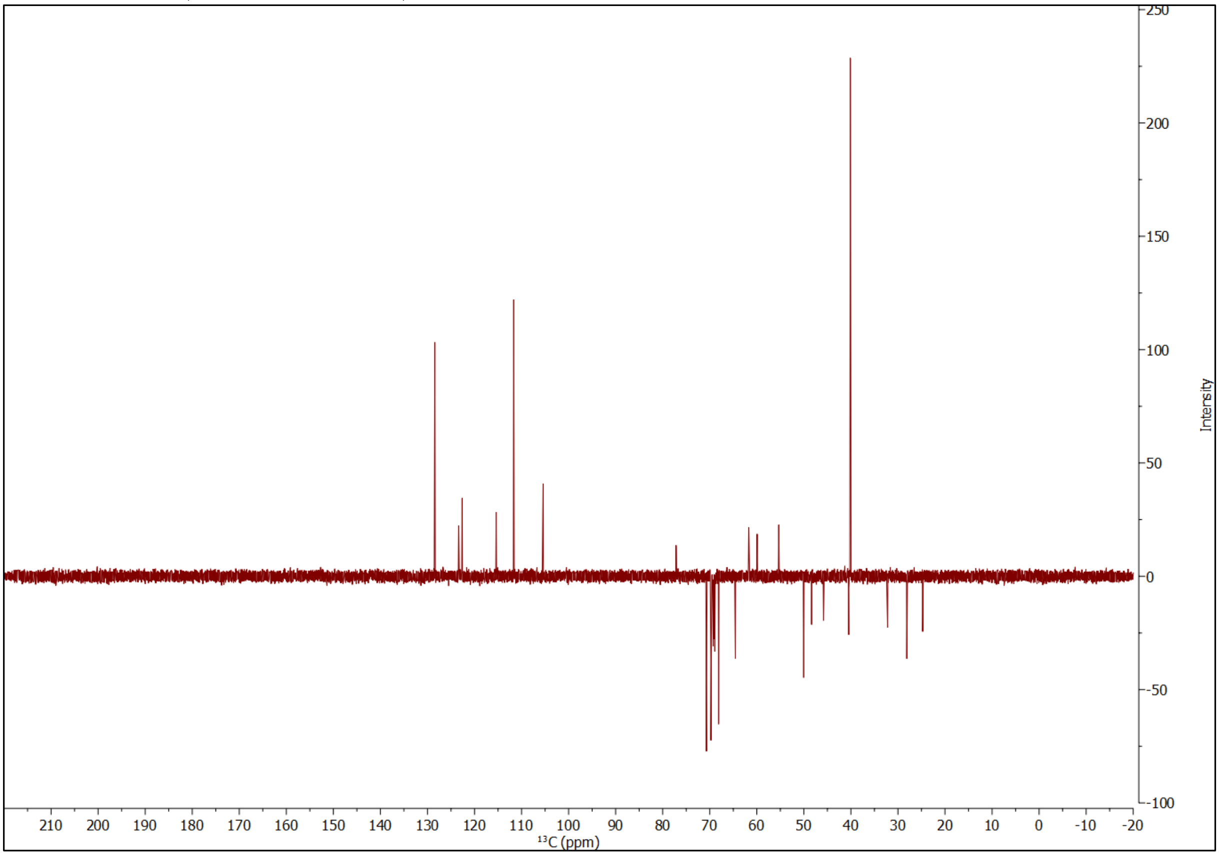

^{1}H-^{1}H-COSY NMR (CDCl$_3$, **18**, cCAP-1)

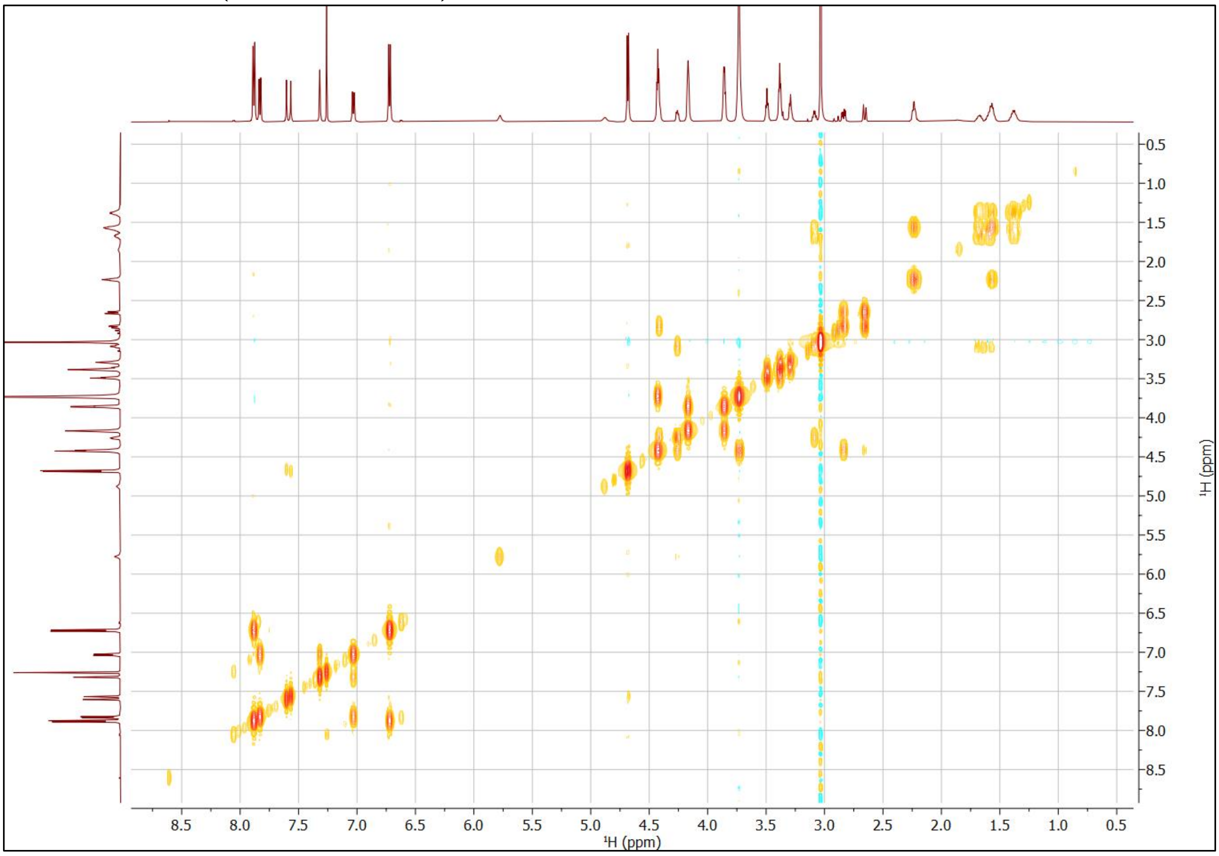

^{1}H-^{13}C-HSQC NMR ($CDCl_3$, **18**, cCAP-1)

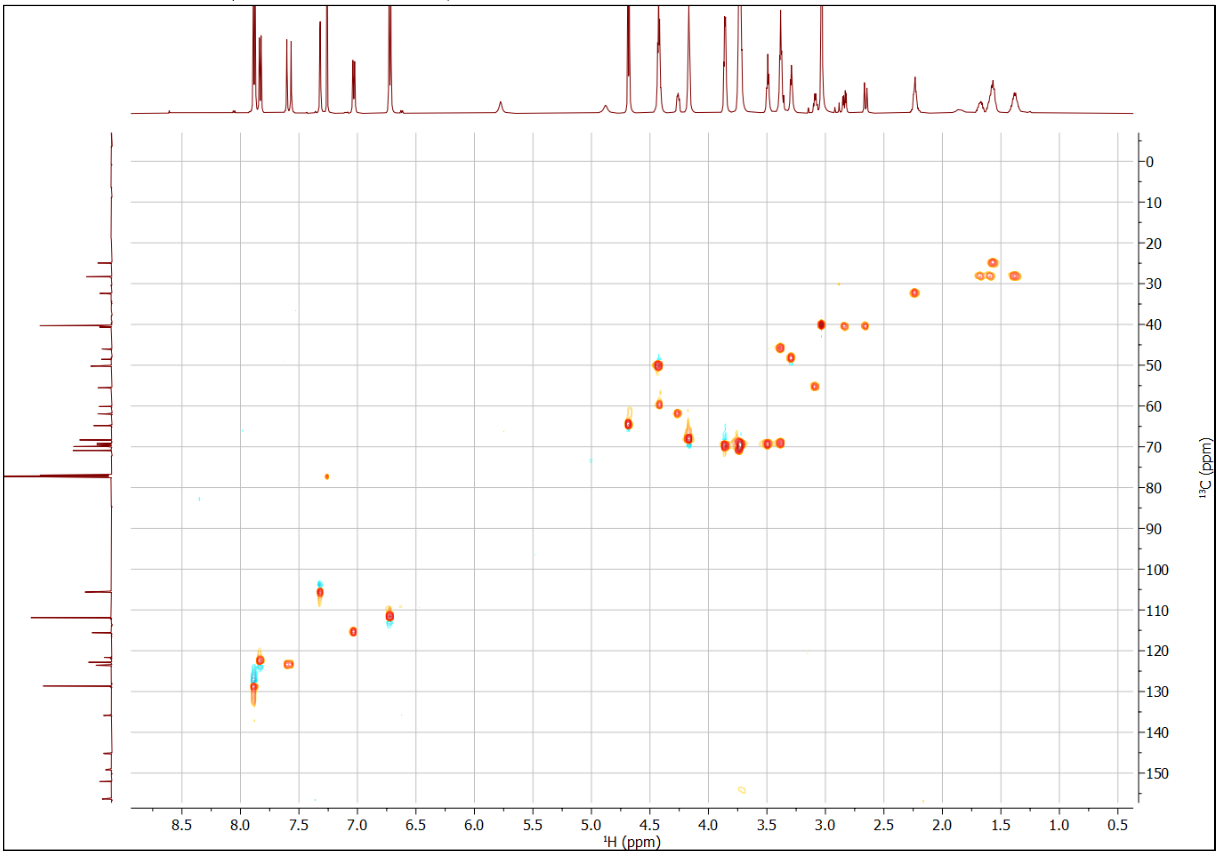

^{1}H-^{13}C-HMBC NMR ($CDCl_3$, **18**, cCAP-1)

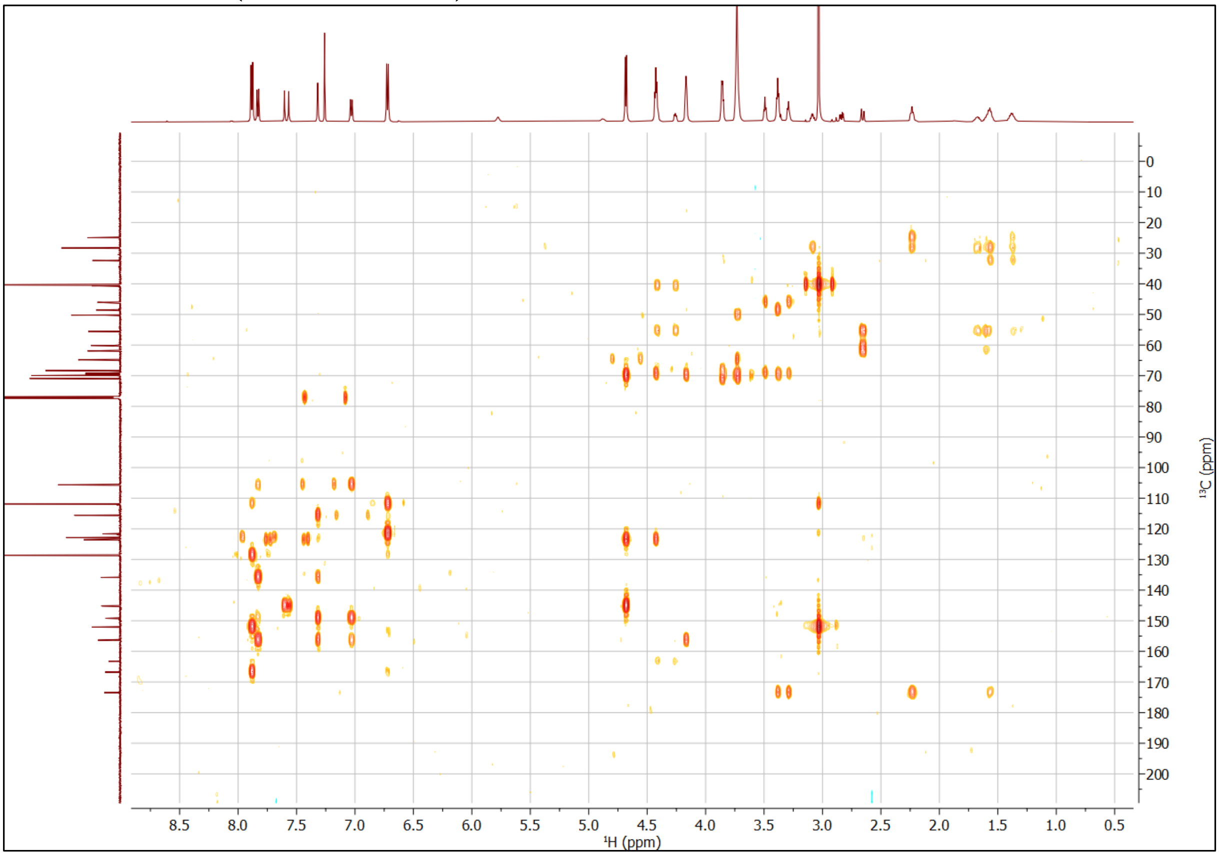

^{1}H-^{1}H-ROESY NMR ($CDCl_3$, **18**, cCAP-1)

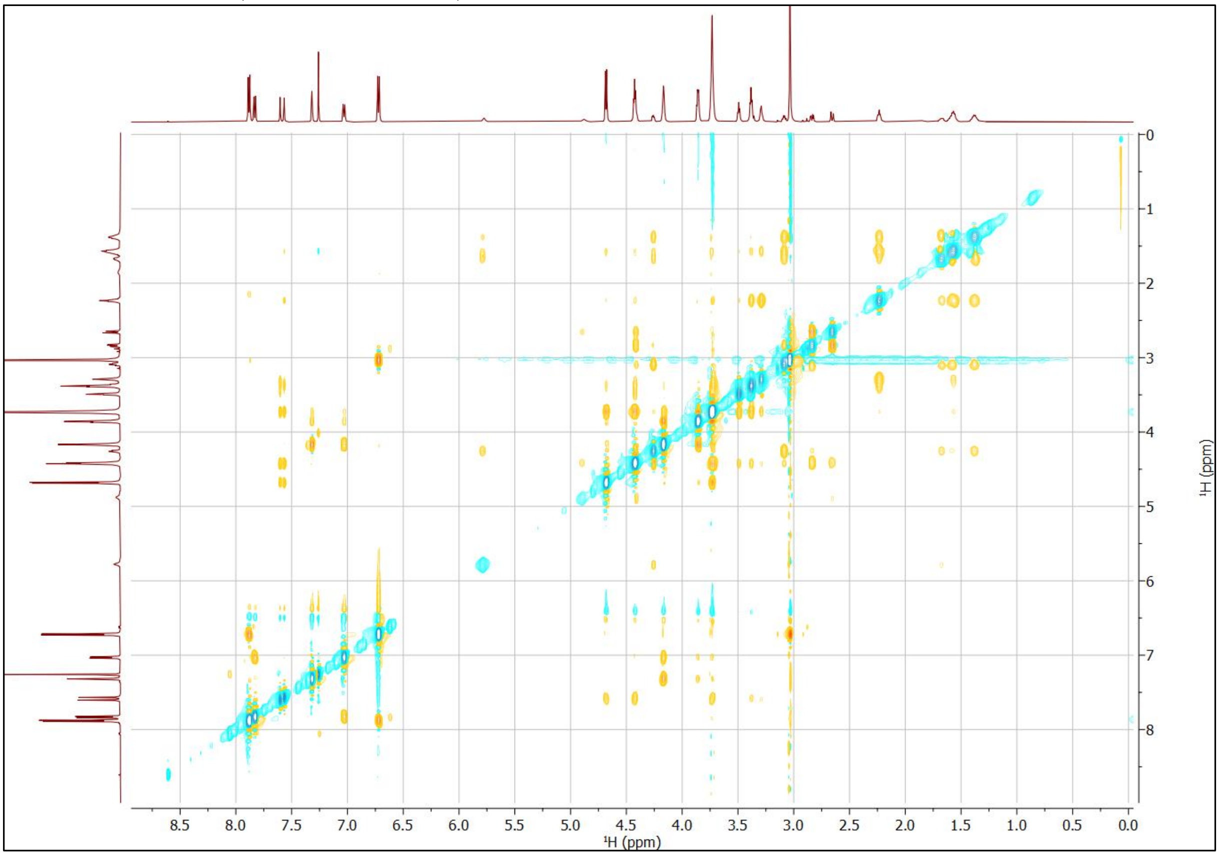

^{1}H-^{1}H-TOCSY NMR ($CDCl_3$, **18**, cCAP-1)

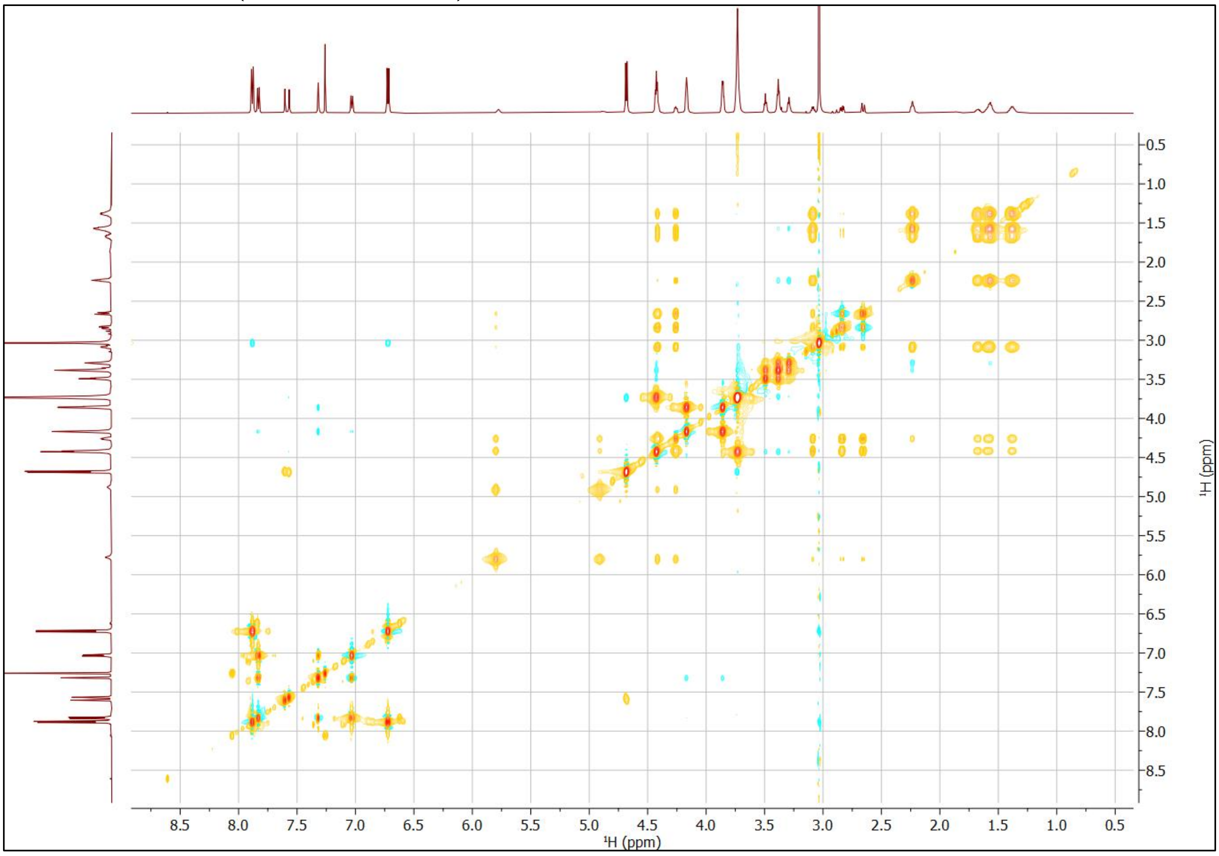

6. References

[1] D. R. Burfield, R. H. Smithers, *J. Org. Chem.* **1978**, *43*, 3966-3968; 'Desiccant Efficiency in Solvent Drying. 3. Dipolar Aprotic Solvents'.

[2] T. M. Weber, J. Pietruszka, *Synthesis* **2023**, *55*, 2128-2133; 'Synthesis of a Water-Soluble Tridentate (Dimethylamino)ethyl Cu(I)/Cu(II)-Ligand'.

[3] A. V. Bordunov, P. C. Hellier, J. S. Bradshaw, N. K. Dalley, X. Kou, X. X. Zhang, R. M. Izatt, *J. Org. Chem.* **1995**, *60*, 6097-6102; 'Synthesis of New Pyridinoazacrown Ethers Containing Aromatic and Heteroaromatic Proton Ionizable Substituents'.

[4] B. Siewert, M. Langerman, A. Pannwitz, S. Bonnet, *Eur. J. Inorg. Chem.* **2018**, *2018*, 4117-4124; 'Synthesis and Avidin Binding of Ruthenium Complexes Functionalized with a Light-Cleavable Free Biotin Moiety'.

[5] C. A. Mathis, Y. Wang, D. P. Holt, G.-F. Huang, M. L. Debnath, W. E. Klunk, *J. Med. Chem.* **2003**, *46*, 2740-2754; 'Synthesis and Evaluation of ^{11}C-Labeled 6-Substituted 2-Arylbenzothiazoles as Amyloid Imaging Agents'.

[6] Y. Huang, H.-J. Cho, N. Bandara, L. Sun, D. Tran, B. E. Rogers, L. M. Mirica, *Chem. Sci.* **2020**, *11*, 7789-7799; 'Metal-chelating benzothiazole multifunctional compounds for the modulation and ^{64}Cu PET imaging of Aβ aggregation'.
